# Supplementary material for: Comparative Analysis of Type 1 and Type Z Protein Phosphatases Reveals D615 as a Key Residue for Ppz1 Regulation
Source: Int J Mol Sci. 2022 Jan 25;23(3):1327. doi: 10.3390/ijms23031327 (PMC8836105; doi:10.3390/ijms23031327)
Supplement: Supplementary file 1 [file ijms-23-01327-s001.zip › Figure S1 global consensus.pdf]

Figure S1. Alignment and consensus sequence from fungal PP1 and PPZ phosphatases. Alignment was performed with Clustal Omega. Consensus sequences and shading of residues was performed with pyBoxshade (https://github.com/mdbaron42/pyBoxshade). The threshold fraction for consensus was set to 0.8. For a given phosphatase **residue** denotes that all residues are the same, **residue** denotes that it is identical to the consensus, **residue** that it is similar to the consensus**,** and **residue** that it is different from the consensus**.** A snapshot of the pyBoxshade preferences setting is presented at the end of the alignments

```
                 ....:...10....:...20....:...30....:...40....:...50....:....
2YaLi_Glc7       ---------madqkeVDLDsVIDRLLEVRG-sRPGKqVqLmEhEIRYLCtKAREIFIqQP
2Mgui_PP1        ---------madhqeaDIDsIvDRLLEVRG-sRPGKqVtLmEhEIRYLCtKAREIFIqQP
2DEHA_Glc7       ---------madhqeaDIDsIvDRLLEVRG-sRPGKqVtLlEhEIRYLCtKAREIFIqQP
2Mfar_PP1        ---------maehqeVDIDsIvDRLLEVRG-sRPGKqVtLlEhEIRYLCtKAREIFIqQP
2Ct_Glc7         -----msnieddssIsVDsIvDRLLEVRG-sRPGKqVaLsEnEIRYLCtKAREIFIqQP
2Cgla_Glc7       ----------mesqqVDVDsIIDRLLEVRG-sKPGqqVdLeEhEIRhLCsKARsIFIkQP
2Ca_Glc7         -----msnnieddsIsVDnIvDRLLEVRG-sRPGKqVtLtEnEIRFLCtKAREIFIqQP
2Cdub_PP1        -----msnniddddIsVDnIvDRLLEVRG-sRPGKqVtLtEnEIRFLCtKAREIFIqQP
2Klac_PP1        ----------mdqqsVDVDdIIDRLLEVRG-lKPGHqVdLeEhEIRYLCsKARsIFIkQP
2Lther_Glc7      ----------metpaVDLDnIIDRLLEVRG-sKPGqqVdLeEhEIRFLCsKARsIFIkQP
2Ag_PP1          --------msmetppVDIDnIIDRLLEVRG-sKPGqqVdLeEhEIRYLCsKARsIFIkQP
2Ecym_Glc7       ----------metppVDIDnIIDRLLEVRG-sKPGqqVdLeEhEIRYLCsKARsIFIkQP
2Tbla_PP1        ----------meatqVDVDnIIDRLLEVRG-sKPGqqVdLeEhEIRYLCsKARsIFIkQP
2Tdel_PP1        ----------metpaVDVDnIIDRLLEVRG-sKPGqqVdLeEhEIRYLCsKARsIFIkQP
2Sc_GLC7         ----------mdsqpVDVDnIIDRLLEVRG-sKPGqqVdLeEnEIRYLCsKARsIFIkQP
1Tmel_Glc7       ---------madqteVDLDsIIDRLLEVRG-sRPGKqVqLlEaEIRYLCtKAREIFIsQP
1Bcin_PP1-A      ----------maepeVDLDsIIDRLLEVRG-sRPGKqVqLlEqEIRYLCtKAREIFIsQP
1Nc_PP1          ---------madhteVDLDsIIDRLLEVRG-sRPGKqVqLlEaEIRYLCtKAREIFIsQP
1Mthe_PP1        ---------madqhdVDLDsIIDRLLEVRG-sRPGKqVqLlEaEIRYLCtKAREIFIsQP
1Bbas_PP1        ---------madqhdVDLDsIIDRLLEVRG-sRPGKqVqLlEsEIRYLCtKAREIFIsQP
1Anam_PP1        ---------madqqdVDLDsIIDRLLEVRG-sRPGKqVqLlEtEIRYLCtRAREIFIsQP
1Npar_PP1        ----------maeqeVDLDsIIDRLLEVRG-sRPGKqVqLlEtEIRYLCtKAREIFIsQP
1Bory_Glc7       ----------maeteVDLDsIIDRLLEVRG-sRPGKqVqLlEtEIRYLCtKAREIFIsQP
1Uree_PP1        ---------madqneIDLDsIIDRLLEVRG-sRPGKqVqLlEsEIRhLCtKAREIFIsQP
1Tver_PP1        ---------madqneIDLDsIIDRLLEVRG-sRPGKqVqLlEsEIRhLCtKAREIFIsQP
1Anid_Glc7       ----------madqdVDLDsIIDRLLEVRG-sRPGKqVqLlEsEIRYLCtKAREIFIsQP
1Aory_Glc7       ---------msekeeVDLDsIInRLLEVRG-sRPGKhVrLqDsEIqYLChKAREvFIsQP
1Af_PP1          ----------madqeVDLDnIIDRLLEVRG-sRPGKqVqLlEaEIRYLCtKAREIFIsQP
3Sp_PP1-1        ----------msnpdVDLDsIIDRLLEVRG-sRPGRqVqLsEdEIRFLCnKAREIFIsQP
3Sp_PP1-2        -------------mdyDIDaIIEKLvkaRn-gKPsKqVqLsDaEIRYLCttsRsIFLsQP
3Sj_PP1          ----------mskqdVDLDsVIDRLLEVRG-sRPGKqVqLaEhEIRFLCnKAREIFIsQP
3Sj_Sds21        -------------mepDVDtIIEKLLkVRn-nKPGKqVqLaDsEIRFLCsKAREIFLeQP
3Scot_PP1        ----------msnqdVDLDsIIDRLLEVRv-nRPGKqVqLaEnEIRFLCnKAREIFIsQP
3Scot_Sds21      -------------mdyDIDnIIEKLIkVqh-vKPGRqtqLsEaEIRYLCktARsLFLdQP
3Scry_PP1        ----------msnqdVDLDsIIDRLLEVRG-nRPGKqVqLaEnEIRFLCnKAREIFIsQP
3Scry_Sds21      -------------mdyDIDtIIEKLIkVqh-vKPGRqtqLtEaEIRYLCttsRsLFLnQP
3Plac_PP1        --------measnteIDIDqVIaRLLEVRG-sRPGKqVqLpEyEIKFLCtKAREIFInQP
3Pjir_PP1        ---------madqheIDLDsVIERLLEVRG-sRPGRqVqLaEcEIRFLCiKAREIFvnQP
3Nirr_PP1        ---------madqneIDLDnIIERLLEVRG-nRPGKqVtLgEyEIRYLCtKAREIFInQP
4GALMADRAFT_Glc7 --------madtateIDLDsVIDRLLEVRG-nRPGKpVqLaEyEIKYLCtKAREIFInQP
4PILCRDRAFT_Glc7 --------madstteIDLDsVIDRLLEVRG-nRPGKpVqLqEyEIKYLCtKARELFInQP
4Shir_Glc7       --------madsateIDLDsVIDRLLEVRG-nRPGKpVqLqEyEIKYLCtRAREIFInQP
4Sver_PP1        --------maestteIDLDsVIDRLLEVRG-nRPGKpVqLqEyEIKYLCtRAREIFInQP
4Pgig_PP1        --------madttteIDLDsVIDRLLEVRG-nRPGKpVqLqEyEIKYLCtKAREIFInQP
4Bbot_PP1        --------maesspeIDLDsVIDRLLEVRG-nRPGKpVqLqEyEIKhLCmKAREIFInQP
4Cvis_PP1-A      --------maeaqaeIDLDsVIDRLLEVRG-nRPGKpVqLqEyEIKYLCtKAREIFInQP
4Cvis_PP1-B      -----mnlndiqkeVDIDsVIDRLLEVRG-nRPGRnVnLhEyEIKYLCaKAREIlIaQP
4Cgat_PP1        ----------mgeqpeIDLDsVIDRLLEVRG-nRPGKaVqLaEyEIKYLCtKAREIFIsQP
4Cneo_PP1        ----------mgeqpeIDLDsVIDRLLEVRG-nRPGKaVqLaEyEIKYLCtKAREIFIsQP
4Tmes_PP1        --------madqtneIDLDsVIDRLLEVRG-nRPGKaVqLqEyEIKYLCtKAREIFIsQP
4Umay_Glc7       -------madnttleVDLDnVIDRLLEVRG-sRPGKpVhLeEyEIKYLClKARDIFInQP
4Phub_PP1        -------madnstpeVDLDnVIDRLLEVRG-sRPGKpVhLeEyEIKYLClKARDIFInQP
5Wich_Glc7       ----------mseaeVDLDsVIDRLLEVRG-nRPGKpVqLqEyEIKYLCtKAREIFIsQP
5Rgra_PP1        --------maaegqdIDLDsIIDRLLEVRG-nRPGKqVqLqEyEIKFLCtKAREIFInQP
5Pgra_PP1        -------madqggqdIDLDsVIDRLLEVRG-nRPGKqVqLqEyEIKFLCtRAREIFInQP
6Rdel_PP1        ----------mtevpeVNVDsIIERLLEVRG-nRPGKqVqLgEqEIRYLCltAREIFMsQP
6Ccor_PP1        msqilnpnaggseeVDINsIIDRLLEVRG-sRPGKhVlLaEhEIKYLCttAREIFLgQP
6Crev_PP1        ----------maddgVNVDsIIqRLLEVRG-sRPGKeVqLhEaEIHWLCvtAREIFInQP
6Spun_PP1        --------mmaenqeVDVDsIIERLLEVRG-sRPGKqVqLaEhEIRYLCtKsREIFInQP
6Gpro_PP1-A      --------mgepsqdVDVDsIIERLLEVRG-sRPGKqVqLaEhEIRYLCtKsREIFInQP
6Gpro_PP1-B      ---------maeqqvVDIDsIIDRLLEVRG-nRPGKqVqLaEhEIRYLClKAREIFvnQP
6AMAG_PP1-A      ----------mapprrat----hRwh-rar-vRvGKqrpv-----Rsq--vrpsrqrvsP
6Rallo_PP1       ---------msaepeVDVDsIIERLLEVRG-sRPGKqVqLaEhEIRhLCvKsREIFMsQP


                 ....:...10....:...20....:...30....:...40....:...50....:...60
2YALI_Ppz1       -------------mtlnIDevIqRLLDaGYsgKiTKaVcLKNsEIalICrrARsIlLsQP-
2Mgui_Ppz1       -------------ntidIEdlIqRLLDaGYsgKkSKnLcLKNsEIqlVCakAREIFLsQP-
2DEHA_Ppz1       -------------gsidIEnlIqRLLDaGYsgKkTKsVcLKNhEIqlICakARNIlLsQP-
2Mfar_Ppz        -------------nsidIEnlIqRLLDaGYsgKkTKsLcLKNqEIqlVCatAREIFLqQP-
2Ct_Ppz1         ------------tnssqIDvwIdKLInaAYs-KrTKnVcLKNsEIeiICssAREIFLsQP-
2Cgla_Ppz1       -------------kqvnIDeaIqRLLDvGYsgKknKafcLKNsEIiqICiqAREVFLnQP-
```

```
2Cgla_Ppz2        -------------kpidIDdtIqKLLDaGYaaKrTKnVcLKNsEIaqICqkAREIFLaQP-
2Ca_Ppz1          -------------ntidIDslIdKLLnaGFsgKrTKnVcLKNtEIelICasAREIFLsQP-
2Cdub_Ppz         -------------ntidIDslIdKLLnaGFsgKrTKnVcLKNsEIelICasAREIFLsQP-
2Klac_PpzA        -------------kpidIDetIqKLLDaGYsaKkTRsVaLKNsEItqICqmAREIFLsQP-
2Klac_PpzB        -------------kpidIDetIqKLLDaGYsaKkTRsVaLKNsEItqICqmAREIFLsQP-
2Lther_Ppz1       -------------rpldIDetIqKLLDaGYsgKrTKnVcLKNsEIaqICnqvREIFLsQP-
2Ag_Ppz           -------------kpldIDemIqKLLDaGYlgKrTRnVcLKNsEIaqIChlAREIFLsQP-
2Ecym_Ppz         -------------kpldIDemIqKLLDaGYlgKrTRnVcLKNsEvtqIChqAREIFLsQP-
2Tbla_PpzA        -------------kpidIDltIqKLLDaGYaaKkTKnVcLKNsEIaqIChlvREIFLsQP-
2Tdel_PpzA        -------------rpinIDetIqKLLDaGYagKrTKnVcLKNsEIarIChmAREMFLsQP-
2Sc_PPZ1          -------------kpidIDetIqKLLDaGYaaKrTKnVcLKNnEIlqICikAREIFLsQP-
2Sc_PPZ2          -------------rpvdIDeiIqRLLDaGYaaKrTKnVcLKNsEIiqIChkARELFLaQP-
1Tmel_Ppz1        --------------------mItRLLDaGYagKvTKaVcLKNaEItaIChaAREVFLsQs-
1Bcin_Ppz         -------------kdkdLDdyIsRLLDaGYqgKsTKgVcLKsaEInsIClrAREIvLeQP-
1Nc_PP-Z          -------------kdidLDdfIkRLLDaAYagKvTKsVcLKNaEIvaIChrARELFLsQP-
1Mthe_Ppz         -------------kdidLDdfIkRLLDaAYagKvTKsVcLKNaEItaIChrAREVlLtQP-
1Bbas_Ppz         -------------kemdLDdyIkRLLDaGYagKvTKsVcLKNaEIvaICqrAREVlLsQP-
1Anam_Ppz         -------------ktsdLDdmIkRLIDaGYagKvTKtVcLKNaEIfaICqaAREVFLsQP-
1Npar_Ppz1        -------------ksidIDdmIsRLLDaGYsaKvTKtVcLKNaEIfaICqaARELFLsQP-
1Bory_Ppz1        -------------ktmdLDdmIqRLLDaAYagKvTKtVsLKNaEIfaICsaAREVFLsQP-
1Uree_Ppz         -------------ksidVDdmItRLLDaGYsaKvTKaVcLKNaEIiaICaaARELlLsQP-
1Tver_Ppz         -------------ksidVDdmItRLLDaGYstKvTKaVcLKNaEIiaVCtaARELlLtQP-
1Anid_Ppz1        -------------ksidLDdmItRLLDaGYstKvTKtVcLKNaEItaICtaARELFLsQP-
1Aory_Ppz1        ------------lksidLDdmItRLLDaGYstKvTKtVcLKNaEItaICtaARELFLsQP-
1Af_PPZ           ------------lksidLDdmIsRLLDaGYstKvTKtVcLKNaEImaICsaARELFLsQP-
3Sp_PPZ           -------------vslnVDemIqRLIhvGYsrKsSKsVcLKNaEItsICmavREIFLsQP-
3Sj_Pzh1          -------------gglnVDemIqRLIhaGYsrKsTKsVcLKNaEItsICmavsEIFLsQP-
3Soct_Ppz         -------------vslnVDemIqRLIhaGYsrKsSKsVcLKNaEIvsICmavREIFLsQP-
3Scry_Ppz         -------------vslnVDemIqRLIhaGYsrKsSKsVcLKNaEIvsICtavREIFLsQP-
3Plac_Ppz         -------------knidVDdlIqRLLDaGYtgKaTKsVvLKNaEIvaICaavREIlLsQP-
3Pjir_Ppz-A       ----------------------------------------------------------QP-
3Nirr_PP-Z        -------------aglaLDdyIsRLLDaGYsaKvTKtVcLKNaEIfqIClaARDtFLaQs-
4GALMADRAFT_Ppz1  -------------kqfdVDdmIqRLLDvGYtgKvSKsLcLKNtEItaIClaARDVFLsQP-
4PILCRDRAFT_Ppz1  -------------ksldVDdmIqRLLDvGYtgKvSKsLcLKNaEIagICqaAREVFLnQP-
4Shir_Ppz         -------------ksfdIDdmIqRLLDvGYtgKvSKsLcLKNvEIaaICqaAREVFLsQP-
4Sver_PPZ         -------------rtldIDdmIaRLLEvGYsgKvSKhVcLKNtEItsLClaAREVFLsQP-
4Pgig_PPZ         -------------apldIDdmIqRLLDvGYtgKvSKsLcLKNtEItaICqaAREVFLnQP-
4Bbot_PPZ         -------------rtfdIDdmIsRLLEvGYsgKaSKaVcLKNnEItaICqaAREVFLsQP-
4Cvis_PPZ         -------------ntidIDslIsRLIatAYtgKvSKsLpLKtsEItsIClaARDIFLsQP-
4Cgat_PPZ         --------------------mIqRLLEaGYsgKvTKsppLKNaEIvsVCaaAREVFLsQP-
4Cneo_PPZ         -------------qildVDnmIqRLLEaGYsgKvTKsppLKNaEItsVCaaAREVFLsQP-
4Tmes_PPZ         -------------gamdLDnmIsRLLEaGYsgKvTKsppLKNaEIanVCaaAREVFLsQP-
4Umay_PPZ         ------------vrtfdIDdmIsRLLEaGYsgKipK-paLKNaEItaVCqaAREIFLsQP-
4Phub_PPZ         ------------vrtfdIDdmIsRLLEaGYsgKipK-paLKNaEItaVCqaAREIFLsQP-
5Wich_PP-Z        -------------gsidIDdmIqRLLniGYsaKySKsLpIKpeELtqLlqlsRNLlLnQP-
5Rgra_PPZ1        --------------------mIsRLLDaGYsgKvTKsVcLKNaEItaICqaAREVFLsQP-
5Pgra_PPZ         -------------qafdIDeiInRLLDvGYstKiTKsVcLKNsEItaIslaAREVlLsQP-
6Rdel_PPZ-A       -------------sttnIDeyInRLLEaGYssKvSKqLcLRhsEvtaICraAmDIFLsQP-
6Rdel_PPZ-B       -------------wnldIDecIhRLLDiGlktKvpKsIcfRNsEIvaICraAqQVFLsQP-
Rdel_PPZ-C        -------------sttnIDdyInRLLEaGYssKvSKqLcLRssEvtaICraAmDIFLeQP-
6Rdel_PPZ-D       -------------dglqIDdyIrRLLDaGYasKvpKqLcLKNaEvtlICrtAmEIFLsQPy
6Rdel_PPZ-E       ----------------MNscIkRLIqvGmnkqigKsVcLnpsEItaICrsvyEIFMnQP-
6Rdel_PPZ-G       -------------sttnIDeyInRLLEaGYssKvSKqLcLRhsEvtaICraAmDIFLsQP-
6Ccor_PPZ         -------------gginIDdcIsRLLEaGstaKtpKaLdIeawEIdsICalsREIFLsQP-
6Crev_PPZ         -------------kqfsVDdmIfRLLDaGFsgRvSKsVcLRNsEIvaVChaAREVFLaQP-
6Spun_PPZ         ------------kdkydLDdiIaRLLDarn-hKvSKsLcLKNsEvlsVChkAREVFMsQP-
6Gpro_PPZ         ------------aaeisIDdiIqRLLgqr-gaKpgKaLpvKNqEImfVCqrAREVFLdQPi
6AMAG_PPZ-A       ------------aktldIDdvIhRLInaGs-gKvgRnLpLKNsEIitIlhaARDIFLsQP-
6AMAG_PPZ-B       -----------------MgsgqsKfLDaGr-gKngvaapn---------------------
6Rallo_Ppz1       -------------vfvdIDeiIqRLLEiknqpKlqRyMvvKtnEIlsICsiAREIlLaEP-

consensus PP1                        vdld iidrllevrg  rpgk v l e eirylc kareifi qP
consensus PPZ                          id  i rlld gy  k tk v lkn ei  ic  areifl qp
```

```
                 ....:....10....:....20....:....30....:....40....:....50....:....60
2YaLi_Glc7       -ILLELEAPIKICGDIHGQYYDLLRLFEYGGFPPEANYLFLGDYVDRGKQSLETICLLLA
2Mgui_PP1        -ILLELEAPIKICGDIHGQYYDLLRLFEYGGFPPEANYLFLGDYVDRGKQSLETICLLLA
2DEHA_Glc7       -ILLELEAPIKICGDIHGQYYDLLRLFEYGGFPPEANYLFLGDYVDRGKQSLETICLLLA
2Mfar_PP1        -ILLELEAPIKICGDIHGQYYDLLRLFEYGGFPPEANYLFLGDYVDRGKQSLETICLLLA
2Ct_Glc7         -ILLELEAPIKICGDIHGQYYDLLRLFEYGGFPPEANYLFLGDYVDRGKQSLETICLLLA
2Cgla_Glc7       -ILLELEAPIKICGDIHGQYYDLLRLFEYGGFPPEsNYLFLGDYVDRGKQSLETICLLLA
2Ca_Glc7         -ILLELEAPIKICGDIHGQYYDLLRLFEYGGFPPEANYLFLGDYVDRGKQSLETICLLLA
2Cdub_PP1        -ILLELEAPIKICGDIHGQYYDLLRLFEYGGFPPEANYLFLGDYVDRGKQSLETICLLLA
2Klac_PP1        -ILLELEAPIKICGDIHGQYYDLLRLFEYGGFPPEsNYLFLGDYVDRGKQSLETICLLLA
2Lther_Glc7      -ILLELEAPIKICGDIHGQYYDLLRLFEYGGFPPEsNYLFLGDYVDRGKQSLETICLLLA
2Ag_PP1          -ILLELEAPIKICGDIHGQYYDLLRLFEYGGFPPEsNYLFLGDYVDRGKQSLETICLLLA
2Ecym_Glc7       -ILLELEAPIKICGDIHGQYYDLLRLFEYGGFPPEsNYLFLGDYVDRGKQSLETICLLLA
2Tbla_PP1        -ILLELEAPIKICGDIHGQYYDLLRLFEYGGFPPEsNYLFLGDYVDRGKQSLETICLLLA
2Tdel_PP1        -ILLELEAPIKICGDIHGQYYDLLRLFEYGGFPPEsNYLFLGDYVDRGKQSLETICLLLA
2Sc_GLC7         -ILLELEAPIKICGDIHGQYYDLLRLFEYGGFPPEsNYLFLGDYVDRGKQSLETICLLLA
1Tmel_Glc7       -ILLELEAPIKICGDIHGQYYDLLRLFEYGGFPPEANYLFLGDYVDRGKQSLETICLLLA
1Bcin_PP1-A      -ILLELEAPIKICGDIHGQYYDLLRLFEYGGFPPEANYLFLGDYVDRGKQSLETICLLLA
1Nc_PP1          -ILLELEAPIKICGDIHGQYYDLLRLFEYGGFPPEANYLFLGDYVDRGKQSLETICLLLA
1Mthe_PP1        -ILLELEAPIKICGDIHGQYYDLLRLFEYGGFPPEANYLFLGDYVDRGKQSLETICLLLA
1Bbas_PP1        -ILLELEAPIKICGDIHGQYYDLLRLFEYGGFPPEANYLFLGDYVDRGKQSLETICLLLA
1Anam_PP1        -ILLELEAPIKICGDIHGQYYDLLRLFEYGGFPPEANYLFLGDYVDRGKQSLETICLLLA
1Npar_PP1        -ILLELEAPIKICGDIHGQYYDLLRLFEYGGFPPEANYLFLGDYVDRGKQSLETICLLLA
1Bory_Glc7       -ILLELEAPIKICGDIHGQYYDLLRLFEYGGFPPEANYLFLGDYVDRGKQSLETICLLLA
1Uree_PP1        -ILLELEAPIKICGDIHGQYYDLLRLFEYGGFPPEANYLFLGDYVDRGKQSLETICLLLA
1Tver_PP1        -ILLELEAPIK--------YYDLLRLFEYGGFPPEANYLFLGDYVDRGKQSLETICLLLA
1Anid_Glc7       -ILLELEAPIKICGDIHGQYYDLLRLFEYGGFPPEANYLFLGDYVDRGKQSLETICLLLA
1Aory_Glc7       -ILLELEAPIqICGDvHGQYYDLLRLFEYGGYPPDANYLFLGDYVDRGKQSvEcICLLLA
1Af_PP1          -ILLELEAPIKICGDIHGQYYDLLRLFEYGGFPPEANYLFLGDYVDRGKQSLETICLLLA
3Sp_PP1-1        -ILLELEAPLKICGDIHGQYYDLLRLFEYGGFPPEANYLFLGDYVDRGKQSLEvICLLLA
3Sp_PP1-2        -MLLELEAPLKICGDIHGQYsDLLRLFEYGGYPPDANYLFLGDYVDRGKQSLEvICLLfA
3Sj_PP1          -ILLELEAPLKICGDIHGQYYDLLRLFEYGGFPPEANYLFLGDYVDRGKQSLETICLLLA
3Sj_Sds21        -ILLELEAPLKICGDIHGQYhDLLRLFEYGGFPPDANYLFLGDYVDRGKQSLETICLLfA
3Scot_PP1        -ILLELEAPLKICGDIHGQYYDLLRLFEYGGFPPEANYLFLGDYVDRGKQSLEvICLLLA
3Scot_Sds21      -MLLELEAPLKICGDIHGQYsDLLRLFEYGGFPPDANYLFLGDYVDRGKQSLEvICLLfA
3Scry_PP1        -ILLELEAPLKICGDIHGQYYDLLRLFEYGGFPPEANYLFLGDYVDRGKQSLEvICLLLA
3Scry_Sds21      -MLLELEAPLKvCGDIHGQYsDLLRLFEYGGFPPDtNYLFLGDYVDRGKQSLEvICLLfA
3Plac_PP1        -ILLELEAPIKICGDIHGQYYDLLRLFEYGGFPPEANYLFLGDYVDRGKQSLETICLLLA
3Pjir_PP1        -ILLELEAPIKICGDIHGQYYDLLRLFEYGGFPPEANYLFLGDYVDRGKQSLETICLLLA
3Nirr_PP1        -ILLELEAPIKICGDIHGQYYDLLRLFEYGGFPPEANYLFLGDYVDRGKQSLETICLLLA
4GALMADRAFT_Glc7 -ILLELEAPIKICGDIHGQYYDLLRLFEYGGFPPEANYLFLGDYVDRGKQSLETICLLLA
4PILCRDRAFT_Glc7 -ILLELEAPIKICGDIHGQYYDLLRLFEYGGFPPEANYLFLGDYVDRGKQSLETICLLLA
4Shir_Glc7       -ILLELEAPIKICGDIHGQYYDLLRLFEYGGFPPEANYLFLGDYVDRGKQSLETICLLLA
4Sver_PP1        -ILLELEAPIKICGDIHGQYYDLLRLFEYGGFPPEANYLFLGDYVDRGKQSLETICLLLA
4Pgig_PP1        -ILLELEAPIKICGDIHGQYYDLLRLFEYGGFPPEANYLFLGDYVDRGKQSLETICLLLA
4Bbot_PP1        -ILLELEAPIKICGDIHGQYYDLLRLFEYGGFPPEANYLFLGDYVDRGKQSLETICLLLA
4Cvis_PP1-A      -ILLELEAPIKICGDIHGQYYDLLRLFEYGGFPPEANYLFLGDYVDRGKQSLETICLLLA
4Cvis_PP1-B      -ILLELEAPIKICGDIHGQYYDLLRLFEYGGFPPEANYLFLGDYVDRGKQSLETICLLfA
4Cgat_PP1        -ILLELEAPIKICGDIHGQYYDLLRLFEYGGFPPEANYLFLGDYVDRGKQSLETICLLLA
4Cneo_PP1        -ILLELEAPIKICGDIHGQYYDLLRLFEYGGFPPEANYLFLGDYVDRGKQSLETICLLLA
4Tmes_PP1        -ILLELEAPIKICGDIHGQYYDLLRLFEYGGFPPEANYLFLGDYVDRGKQSLETICLLLA
4Umay_Glc7       -ILLELEAPIKICGDIHGQYYDLLRLFEYGGFPPEANYLFLGDYVDRGKQSLETICLLLA
4Phub_PP1        -ILLELEAPIKICGDIHGQYYDLLRLFEYGGFPPEANYLFLGDYVDRGKQSLETICLLLA
5Wich_Glc7       -ILLELEAPIKICGDIHGQYYDLLRLFEYGGFPPEANYLFLGDYVDRGKQSLETICLLLA
5Rgra_PP1        -ILLELEAPIKICGDIHGQYYDLLRLFEYGGFPPEANYLFLGDYVDRGKQSLETICLLLA
5Pgra_PP1        -ILLELEAPIKICGDIHGQYYDLLRLFEYGGFPPEANYLFLGDYVDRGKQSLETICLLLA
6Rdel_PP1        -ILLDLEAPIKICGDIHGQYYDLLRLFEYGGFPPEsNYLFMGDYVDRGKQSLETICLLLA
6Ccor_PP1        -ILLELEAPIKICGDIHGQYYDLLRLFEYGGFPPEANYLFLGDYVDRGKQSLETICLLLA
6Crev_PP1        -vLLELEAPIKICGDIHGQYYDLLRLFEYGGFPPEANYLFLGDYVDRGKQSLETICLLLA
6Spun_PP1        -ILLELEAPIKICGDIHGQYYDLLRLFEYGGFPPEANYLFLGDYVDRGKQSLETICLLLA
6Gpro_PP1-A      -ILLELEAPIKICGDIHGQYYDLLRLFEYGGFPPEANYLFLGDYVDRGKQSLETICLLLA
6Gpro_PP1-B      -vLLDLEAPIKICGDIHGQYYDLLRLFEYGGFPPEsNYLFLGDYVDRGKQSLETIsLLLA
6AMAG_PP1-A      -ILLELEAPIKICGDIHGQYYDLLRLFEYGGFPPEANYLFLGDYVDRGKQSLETICLLLA
6Rallo_PP1       -tLLELDAPIKICGDIHGQYFDLLRLFEYGGFPPEANYLFLGDYVDRGKQSLETICLLLA
```

```
                  ....:...10....:...20....:...30....:...40....:...50....:...60
2YALI_Ppz1        -tLLELlpPVKIVGDVHGQFgDLIRMFdmCGFPPaaNYLFLGDYVDRGKQSLETILLLLC
2Mgui_Ppz1        -sLLELspPVKVVGDVHGQYgDLIRVFtkCGFPPltNYLFLGDYVDRGKQSLETILLLLC
2DEHA_Ppz1        -sLLELspPVKVVGDVHGQYgDLIRIFtkCGFPPqtNYLFLGDYVDRGKQSLETILLLLC
2Mfar_Ppz         -sLLELspPVKVVGDVHGQYgDLIRVFtkCGFPPqtNYLFLGDYVDRGKQSLETILLLLC
2Ct_Ppz1          -sLLELspPVKVVGDVHGQYlDLIRIFskCGFPPktNYLFLGDYVDRGKQSLETILLLLC
2Cgla_Ppz1        -aLLELsaPVKIVGDIHGQFqDLLRLFtkCGFPPssNFLFLGDYVDRGKQSLETILLLLC
2Cgla_Ppz2        -aLLELspsVKIVGDVHGQYgDLLRLFtkCGFPPmaNYLFLGDYVDRGKQSLETILLLLC
2Ca_Ppz1          -sLLELapPVKVVGDVHGQYhDLIRIFskCGFPPktNYLFLGDYVDRGKQSLETILLLLC
2Cdub_Ppz         -sLLELapPVKVVGDVHGQYhDLIRIFskCGFPPktNYLFLGDYVDRGKQSLETILLLLC
2Klac_PpzA        -sLLELspPVKIVGDVHGQYaDLLRLFtkCGFPPsaNYLFLGDYVDRGKQSLETILLLLC
2Klac_PpzB        -sLLELspPVKIVGDVHGQYaDLLRLFtkCGFPPsaNYLFLGDYVDRGKQSLETILLLLC
2Lther_Ppz1       -sLLELspPVKIVGDVHGQYaDLLRLFtkCGFPPsaNYLFLGDYVDRGKQSLETILLLLC
2Ag_Ppz           -sLLELspPVKIVGDVHGQYaDLLRLFtkCGFPPssNYLFLGDYVDRGKQSLETILLLLC
2Ecym_Ppz         -sLLELspPVKIVGDVHGQYaDLLRLFtkCGFPPssNYLFLGDYVDRGKQSLETILLLLC
2Tbla_PpzA        -sLLELssPVKIVGDVHGQYgDLLRLFtkCGFPPaaNYLFLGDYVDRGKQSLETILLLfC
2Tdel_PpzA        -sLLELspPVKIVGDVHGQYaDLLRLFtkCGFPPsaNFLFLGDYVDRGKQSLETILLLLC
2Sc_PPZ1          -sLLELspPVKIVGDVHGQYgDLLRLFtkCGFPPssNYLFLGDYVDRGKQSLETILLLfC
2Sc_PPZ2          -aLLELspsVKIVGDVHGQYaDLLRLFtkCGFPPmaNYLFLGDYVDRGKQSLETILLLLC
1Tmel_Ppz1        -aLVELspPVKIVGDVHGQYtDLIRMFemCGFPPnaNYLFLGDYVDRGKQSLETILLLLC
1Bcin_Ppz         -pLIELeaPVKIVGDIHGQYsDLIRMFemCGFPPssNFLFLGDYVDRGKQSLETILLLLC
1Nc_PP-Z          -aLLELdaPVKIVGDVHGQYtDLIRMFemCGFPPnsNYLFLGDYVDRGKQSLETILLLMC
1Mthe_Ppz         -aLLELdaPVKIVGDVHGQYtDLIRMFemCGFPPksNYLFLGDYVDRGKQSLETILLLLC
1Bbas_Ppz         -aLLELdaPVKVVGDVHGQYtDLIRMFemCGFPPmaNYLFLGDYVDRGKQSLETILLLLC
1Anam_Ppz         -aLLELapaVKIVGDVHGQYtDLLRMFemCGFPPnsNYLFLGDYVDRGKQSLETILLLLC
1Npar_Ppz1        -aLLELspPVKIVGDVHGQYtDLIRMFemCGFPPssNYLFLGDYVDRGKQSLETILLLLC
1Bory_Ppz1        -aLLELaaPVKIVGDIHGQYtDLIRMFemCGFPPnsNYLFLGDYVDRGKQSLETILLLLC
1Uree_Ppz         -aLVELaaPVKIVGDVHGQYtDLIRLFemCGFPPasNYLFLGDYVDRGKQSLETILLLLC
1Tver_Ppz         -aLLELsaPVKIVGDVHGQYtDLIRLFemCGFPPnsNYLFLGDYVDRGKQSLETILLLLC
1Anid_Ppz1        -aLLELsaPVKIVGDVHGQYtDLIRLFemCGFPPvsNYLFLGDYVDRGKQSLETILLLLC
1Aory_Ppz1        -aLLELsaPVKIVGDVHGQYtDLIRLFemCGFPPssNYLFLGDYVDRGKQSLETILLLLC
1Af_PPZ           -aLLELsaPVKIVGDVHGQYtDLIRLFemCGFPPasNYLFLGDYVDRGKQSLETILLLLC
3Sp_PPZ           -tLLELtpPVKIVGDVHGQYsDLIRLFemCGFPPssNYLFLGDYVDRGKQSLETILLLfl
3Sj_Pzh1          -tLLELmpPVKIVGDVHGQYsDLIRLFemCGFPPssNYLFLGDYVDRGKQSLETILLLLl
3Soct_Ppz         -tfLELmpPVKIVGDVHGQYsDLIRLFemCGFPPssNYLFLGDYVDRGKQSLETILLLfl
3Scry_Ppz         -tfLELmpPVKIVGDVHGQYsDLIRLFemCGFPPssNYLFLGDYVDRGKQSLETILLLfl
3Plac_Ppz         -aLLELsaPVKIVGDIHGQFtDLIRLFemCGFPPsaNYLFLGDYVDRGKQSLETILLLMl
3Pjir_Ppz-A       -aLLELsaPVKIVGDIHGQFtDLIRLFemCGFPPsaNYLFLGDYVDRGKQSLETILLLMl
3Nirr_PP-Z        -aLLELapPlKIVGDVHGQYsDLLRLFemCGYPPsaNYLFLGDYVDRGKQSLETILLLLC
4GALMADRAFT_Ppz1  -tLVELspPVKIVGDVHGQYsDLIRLFemCGFPPaaNYLFLGDYVDRGKQSLETILLLLC
4PILCRDRAFT_Ppz1  -tLVELspPVKIVGDVHGQYsDLIRLFemCGFPPaaNYLFLGDYVDRGKQSLETILLLLC
4Shir_Ppz         -tLIELspPVKIVGDVHGQYsDLIRLFemCGFPPaaNYLFLGDYVDRGKQSLETILLLLC
4Sver_PPZ         -tLIELspPVKIVGDVHGQYsDLIRLFemCGFPPsaNYLFLGDYVDRGKQSLETILLLLC
4Pgig_PPZ         -tLIELspPVKIVGDVHGQYsDLIRLFemCGFPPaaNYLFLGDYVDRGKQSLETILLLLC
4Bbot_PPZ         -tLIELspPVKIVGDVHGQYsDLIRLFemCGFPPssNYLFLGDYVDRGKQSLETILLLLC
4Cvis_PPZ         -tLIELspPVKIVGDVHGQYaDLIRLFemCGFPPaaNYLFLGDYVDRGKQSLETILLLLC
4Cgat_PPZ         -tLIELspPVKIVGDVHGQYaDLLRMFemCGFPPaaNYLFLGDYVDRGKQSLETILLLLC
4Cneo_PPZ         -tLIELspPVKIVGDVHGQYaDLLRMFemCGFPPaaNYLFLGDYVDRGKQSLETILLLLC
4Tmes_PPZ         -tLIELspPVKIVGDVHGQYaDLIRMFemCGFPPqaNYLFLGDYVDRGKQSLETILLLLC
4Umay_PPZ         -tLIELspPVKIVGDtHGQYhDLLRLFemCGFPPsaNYLFLGDYVDRGKQSLETILLLLC
4Phub_PPZ         -tLIELspPVKIVGDtHGQYhDLLRLFemCGFPPsaNYLFLGDYVDRGKQSLETILLLLC
5Wich_PP-Z        -tLIELnpPVKIVGDVHGQYsDLIRLFdmsGFPPqsNYLFLGDYVDRGKQSLETILLLLC
5Rgra_PPZ1        -tLVELspPVKIVGDVHGQYsDLIRLFemCGFPPnaNYLFLGDYVDRGKQSLETILLLLC
5Pgra_PPZ         -tLIELspPVKIVGDVHGQYsDLIRLFemCGFPPmaNYLFLGDYVDRGKQSLETILLLLC
6Rdel_PPZ-A       -sLLELspPVKVVGDtHGQYtDLIRLFemgGFPPtsNYLFLGDYVDRGKQSLETILLLfC
6Rdel_PPZ-B       -----------------------------gGYPPdsNYLFLGDYVDRGKQSLETILLLfC
Rdel_PPZ-C        -vdLy--------------WnfhLlLFemgGFPPssNYLFLGDYVDRGKQSLETILLLfC
6Rdel_PPZ-D       ksLLELsaPVKIVGDtHGQYtDLLRLFdmgGFPPssNYLFLGDYVDRGKQSLETfLLLLC
6Rdel_PPZ-E       -aLLQLnpGVKVaGDIHGQFfDLIRLFqmCGYPPstNYLFLGDYVDRGmySLETILLLLC
6Rdel_PPZ-F       ---------------------------mCGYPPstNYLFLGDYVDRGmySLETILLLLC
6Rdel_PPZ-G       -sLLELspPVKVVGDtHGQYtDLIRLFemgGFPPtsNYLFLGDYVDRGKQSLETILLLfC
6Ccor_PPZ         -tLIEIsaPVKIVGDVHGQYtDLLRLFemCGYPPnaNYLFLGDYVDRGRQSLETILLLLC
6Crev_PPZ         -tLLQLaaPVKItGDIHGQYtDLLRLFdkCGYPPhcNYLFLGDYVDRGKQSLETMLLLMC
6Spun_PPZ         -iLLELlpPVnIVGapcqvifta-ssttfCeYstfaDiL---------------------
6Gpro_PPZ         lleLNphpsltIcGDIHGQFeDLLRIFdlmGYPPttNYLFLGDYVDRGKQSLETILLLfC
6AMAG_PPZ-A       -aLIELlpPVKIVGDIHGQFsDLLRLFamsGFPPqaNYLFLGDYVDRGKQSLETILLLLC
6AMAG_PPZ-B       ----------------HsrFl--ayattkrstsGqaNYLFLGDYVDRGKQSLETILLLLC
6Rallo_Ppz1       -sLLQIepPVKIVGDIHGQYlDLLnIFdqCGhPsvqpYLFLGDYVDRGKQSLETMILLLC

Consensus PP1      iLLeLeAPikicgdihgqYyDLLRLFEYGGfPPeaNYLFlGDYVDRGKQSlEtIcLLlA
consensus Ppz      llel  pvkivgdvhgqy dlirlf  cgfpp  nyLflgdyvdrgkqsletillllc
```

```
                   ....:....10....:....20....:....30....:....40....:....50....:....60
2YaLi_Glc7         YKIKYPENFFILRGNHECASIN----RIYGFYDEC-------------KRRYNIKLWKTF
2Mgui_PP1          YKIKYPENFFILRGNHECASIN----RIYGFYDEC-------------KRRYNIKLWKTF
2DEHA_Glc7         YKIKYPENFFILRGNHECASIN----RIYGFYDEC-------------KRRYNIKLWKTF
2Mfar_PP1          YKIKYPENFFILRGNHECASIN----RIYGFYDEC-------------KRRYNIKLWKTF
2Ct_Glc7           YKIKYPENFFILRGNHECASIN----RIYGFYDEC-------------KRRYNIKLWKTF
2Cgla_Glc7         YKIKYPENFFILRGNHECASIN----RIYGFYDEC-------------KRRYNIKLWKTF
2Ca_Glc7           YKIKYPENFFILRGNHECASIN----RIYGFYDEC-------------KRRFNIKLWKTF
2Cdub_PP1          YKIKYPENFFILRGNHECASIN----RIYGFYDEC-------------KRRFNIKLWKTF
2Klac_PP1          YKIKYPENFFILRGNHECASIN----RIYGFYDEC-------------KRRYNIKLWKTF
2Lther_Glc7        YKIKYPENFFILRGNHECASIN----RIYGFYDEC-------------KRRYNIKLWKTF
2Ag_PP1            YKIKYPENFFILRGNHECASIN----RIYGFYDEC-------------KRRYNIKLWKTF
2Ecym_Glc7         YKIKYPENFFILRGNHECASIN----RIYGFYDEC-------------KRRYNIKLWKTF
2Tbla_PP1          YKIKYPENFFILRGNHECASIN----RIYGFYDEC-------------KRRYNIKLWKTF
2Tdel_PP1          YKIKYPENFFILRGNHECASIN----RIYGFYDEC-------------KRRYNIKLWKTF
2Sc_GLC7           YKIKYPENFFILRGNHECASIN----RIYGFYDEC-------------KRRYNIKLWKTF
1Tmel_Glc7         YKIKYPENFFILRGNHECASIN----RIYGFYDEC-------------KRRYNIKLWKTF
1Bcin_PP1-A        YKIKYPENFFILRGNHECASIN----RIYGFYDEC-------------KRRYNIKLWKTF
1Nc_PP1            YKIKYPENFFILRGNHECASIN----RIYGFYDEC-------------KRRYNIKLWKTF
1Mthe_PP1          YKIKYPENFFILRGNHECASIN----RIYGFYDEC-------------KRRYNIKLWKTF
1Bbas_PP1          YKIqYPENFFILRGNHECASIN----RIYGFYDEC-------------KRRYNIKLWKTF
1Anam_PP1          YKIKYPENFFILRGNHECASIN----RIYGFYDEC-------------KRRYNIKLWKTF
1Npar_PP1          YKIKYPENFFILRGNHECASIN----RIYGFYDEC-------------KRRYNIKLWKTF
1Bory_Glc7         YKIKYPENFFvLRGNHECASIN----RIYGFYDEC-------------KRRYNIKLWKTF
1Uree_PP1          YKIKYPENFFvLRGNHECASIN----RIYGFYDEC-------------KRRYNIKLWKTF
1Tver_PP1          YKIKYPENFFvLRGNHECASIN----RIYGFYDEC-------------KRRYNIKLWKTF
1Anid_Glc7         YKIKYPENFFvLRGNHECASIN----RIYGFYDEC-------------KRRYNIKLWKTF
1Aory_Glc7         YKIKYPENFFILRGNHECASIN----RIYGFYDEC-------------KRRYNvKLWKTF
1Af_PP1            YKIKYPENFFvLRGNHECASIN----RIYGFYDEC-------------KRRYNIKLWKTF
3Sp_PP1-1          YKIKYPENFFILRGNHECASIN----RIYGFYDEC-------------KRRYNIKLWKTF
3Sp_PP1-2          YKIKYPENFFLLRGNHEfASIN----RIYGFYDEC-------------KRRYsIKLWKTF
3Sj_PP1            YKIKYPENFFILRGNHECASIN----RIYGFYDEC-------------KRRYNIKLWKTF
3Sj_Sds21          YKIKFPENFFLLRGNHEsAaIN----RIYGFYDEC-------------KRRYsIKLWKqF
3Scot_PP1          YKIKYPENFFILRGNHECASIN----RIYGFYDEC-------------KRRYNIKLWKTF
3Scot_Sds21        FKIKYPENFFLLRGNHECASIN----RIYGFYDEC-------------KRRYNIKIWKvF
3Scry_PP1          YKIKYPENFFILRGNHECASIN----RIYGFYDEC-------------KRRYNIKLWKTF
3Scry_Sds21        FKIKYPENFFLLRGNHECASIN----RIYGFYDEC-------------KRRYNIKIWKiF
3Plac_PP1          YKIKYPENFFILRGNHECASIN----RIYGFYDEC-------------KRRYNIKLWKTF
3Pjir_PP1          YKIKYPENFFILRGNHECASIN----RIYGFYDEC-------------KRRYNIKLWKTF
3Nirr_PP1          YKIKYPENFFILRGNHECASIN----RIYGFYDECiip-gfldtyvagKRRYNIKLWKTF
4GALMADRAFT_Glc7   YKIKYPENFFILRGNHECASIN----RIYGFYDEC-------------KRRYNIKLWKTF
4PILCRDRAFT_Glc7   YKIKYPENFFILRGNHECASIN----RIYGFYDEC-------------KRRYNIKLWKTF
4Shir_Glc7         YKIKYPENFFILRGNHECASIN----RIYGFYDEC-------------KRRYNIKLWKTF
4Sver_PP1          YKIKYPENFFILRGNHECASIN----RIYGFYDEC-------------KRRYNIKLWKTF
4Pgig_PP1          YKIKYPENFFvLRGNHECASIN----RIYGFYDEC-------------KRRYNIKLWKTF
4Bbot_PP1          YKIKYPENFFILRGNHECASIN----RIYGFYDEC-------------KRRYNIKLWKTF
4Cvis_PP1-A        YKIKYPENFFILRGNHECASIN----RIYGFYDEC-------------KRRYNIKLWKTF
4Cvis_PP1-B        YKIKYPENFFMLRGNHEaAStN----RIYGFYDEC-------------KRRYNIKLWKiF
4Cgat_PP1          YKIKYPENFFILRGNHECASIN----RIYGFYDEC-------------KRRYNIKLWKTF
4Cneo_PP1          YKIKYPENFFILRGNHECASIN----RIYGFYDEC-------------KRRYNIKLWKTF
4Tmes_PP1          YKIKYPENFFILRGNHECASIN----RIYGFYDEC-------------KRRYNIKLWKTF
4Umay_Glc7         YKIKYPENFFILRGNHECASIN----RIYGFYDEC-------------KRRFNIKLWKTF
4Phub_PP1          YKIKYPENFFILRGNHECASIN----RIYGFYDEC-------------KRRFNIKLWKTF
5Wich_Glc7         YKIKYPENFFILRGNHECASIN----RIYGFYDEC-------------KRRYNIKLWKTF
5Rgra_PP1          YKIKYPENFFILRGNHECASIN----RIYGFYDEC-------------KRRYNIKLWKTF
5Pgra_PP1          YKIKYPENFFILRGNHECASIN----RIYGFYDEC-------------KRRYNIKLWKTF
6Rdel_PP1          YKIKYPENFFILRGNHECASIN----RIYGFYDEC-------------KRRYNIKLWKTF
6Ccor_PP1          YKIKYPENFFILRGNHECASIN----RIYGFYDEC-------------KRRYNIKLWKiF
6Crev_PP1          YKIKYPENFFvLRGNHEsAcIN----RIYGFYDEC-------------KRRYNIKLWKTF
6Spun_PP1          YKIKYPENFFvLRGNHECASIN----RIYGFYDEC-------------KRRYNIKLWKTF
6Gpro_PP1-A        YKIKYPENFFILRGNHECASIN----RIYGFYDEC-------------KRRYNIKLWKTF
6Gpro_PP1-B        YKIKYPENFFILRGNHEsASIN----RIYGFYDEC-------------KRRYsIsLWKTF
6AMAG_PP1-A        YKIKYPENFFILRGNHECASIN----RIYGFYDECaftvglrlrrierKRRYNIKLWKTF
6Rallo_PP1         YKIKYPENFFILRGNHECASIN----RIYGFYDEC-------------KRRYNIKLWKTF
```

```
                     ....:...10....:...20....:...30....:...40....:...50....:...60
2YALI_Ppz1           YKIKYPENFFILRGNHECAkVT----RVYGFYDEC-------------KRRcNIKtWKtF
2Mgui_Ppz1           YKIKYPENFFLLRGNHEsANVT----RVYGFYDEC-------------KRRcsIKiWKlF
2DEHA_Ppz1           YKIKYPENFFLLRGNHECANVT----RVYGFYDEC-------------KRRcNIKtWKlF
2Mfar_Ppz            YKIKYPENFFLLRGNHECANVT----RVYGFYDEC-------------KRRcNIKtWKlF
2Ct_Ppz1             YKIKYPENFFLLRGNHECANVT----RVYGFYDEC-------------KRRcNIKtWKlF
2Cgla_Ppz1           YKIKYPENFFLLRGNHECANVT----RVYGFYDEC-------------KRRcNIKiWKyF
2Cgla_Ppz2           YKIKYPENFFLLRGNHECANVT----RVYGFYDEC-------------KRRcNIKtWKtF
2Ca_Ppz1             YKIKYPENFFLLRGNHECANVT----RVYGFYDEC-------------KRRcNIKtWKlF
2Cdub_Ppz            YKIKYPENFFLLRGNHECANVT----RVYGFYDEC-------------KRRcNIKtWKlF
2Klac_PpzA           YKIKYPENFFLLRGNHECANVT----RVYGFYDEC-------------KRRcNIKtWKtF
2Klac_PpzB           YKIKYPENFFLLRGNHECANVT----RVYGFYDEC-------------KRRcNIKtWKtF
2Lther_Ppz1          YKIKYPENFFLLRGNHECANVT----RVYGFYDEC-------------KRRcNIKtWKiF
2Ag_Ppz              YKIKYPENFFLLRGNHECANVT----RVYGFYDEC-------------KRRcNIKtWKll
2Ecym_Ppz            YKIKYPENFFLLRGNHECANVT----RVYGFYDEC-------------KRRcNIKtWKvF
2Tbla_PpzA           YKIKYPENFFLLRGNHECANVT----RVYGFYDEC-------------KRRcNIKtWKvF
2Tdel_PpzA           YKIKYPENFFLLRGNHECANVT----RVYGFYDEC-------------KRRcNIKtWKiF
2Sc_PPZ1             YKIKYPENFFLLRGNHECANVT----RVYGFYDEC-------------KRRcNIKiWKtF
2Sc_PPZ2             YKIKYPENFFLLRGNHECANVT----RVYGFYDEC-------------KRRcNIKiWKtF
1Tmel_Ppz1           YKLKFPENFFLLRGNHECANVT----RVYGFYDEC-------------KRRctIKiWKtF
1Bcin_Ppz            YKIKYPENFFLLRGNHECANVT----RVYGFYDEC-------------KRRaNVKvWKtF
1Nc_PP-Z             YKLKYPENFFLLRGNHECANVT----RVYGFYDEC-------------KRRcNVKiWKtF
1Mthe_Ppz            YKLKYPENFFLLRGNHECANVT----RVYGFYDEC-------------KRRcNVKiWKtF
1Bbas_Ppz            YKLKFPENFFLLRGNHECANVT----RVYGFYDEC-------------KRRcNVKiWKtF
1Anam_Ppz            YKLRYPENFFLLRGNHECANVT----RVYGFYDEC-------------KRRcNIKvWKsF
1Npar_Ppz1           YKLKFPENFFLLRGNHECANVT----RVYGFYDEC-------------KRRcNVKvWKaF
1Bory_Ppz1           YKLKFPENFFLLRGNHECANVT----RVYGFYDEC-------------KRRcNIKvWKaF
1Uree_Ppz            YKLKYPENFFLLRGNHECANVT----RVYGFYDEC-------------KRRcNIKiWKtF
1Tver_Ppz            YKLKYPENFFLLRGNHECANVT----RVYGFYDEC-------------KRRcNIKiWKtF
1Anid_Ppz1           YKLKFPENFFLLRGNHECANVT----RVYGFYDEC-------------KRRcNIKiWKtF
1Aory_Ppz1           YKLKYPENFFLLRGNHECANVT----RVYGFYDEC-------------KRRcNIKiWKtF
1Af_PPZ              YKLKYPENFFLLRGNHECANVT----RVYGFYDEC-------------KRRcNIKiWKtF
3Sp_PPZ              YKIRYPENFFLLRGNHECANiT----RVYGFYDEC-------------KRRcNIKiWKtF
3Sj_Pzh1             YKIRYPENFFLLRGNHECANiT----RVYGFYDEC-------------KRRcNIKiWKtF
3Soct_Ppz            YKIRYPENFFLLRGNHECANiT----RVYGFYDEC-------------KRRcNIKiWKtF
3Scry_Ppz            YKIRYPENFFLLRGNHECANiT----RVYGFYDEC-------------KRRcNIKiWKtF
3Plac_Ppz            YKIKYPENFFLLRGNHECANVT----RVYGFYDEC-------------KRRtNIKvWKtF
3Pjir_Ppz-A          YKIKYPENFFLLRGNHECANVT----RVYGFYDEC-------------KRRtNIKvWKtF
3Nirr_PP-Z           YKLKYPNNFFLLRGNHECANVT----RVYGFYDEC-------------KRRtsIKtWKaF
4GALMADRAFT_Ppz1     YKIKYPENFFLLRGNHECANVT----RVYGFYDEC-------------KRRcNIKtWKtF
4PILCRDRAFT_Ppz1     YKIKYPENFFLLRGNHECANVT----RVYGFYDEC-------------KRRcNIKtWKtF
4Shir_Ppz            YKIKYPENFFLLRGNHECANVT----RVYGFYDEC-------------KRRcNIKtWKtF
4Sver_PPZ            YKVKFPENFFLLRGNHECANVT----RVYGFYDEC-------------KRRcsIKiWKtF
4Pgig_PPZ            YKIKYPENFFLLRGNHECANVT----RVYGFYDEC-------------KRRcNIKtWKtF
4Bbot_PPZ            YKVKFPENFFLLRGNHECANVT----RVYGFYDEC-------------KRRcNIKiWKtF
4Cvis_PPZ            YKIKYPENFFMLRGNHECANVT----RVYGFYDEC-------------KRRasLKvWKtF
4Cgat_PPZ            YKIKYPENFFLLRGNHECANVT----RVYGFYDEC-------------KRRtNIKiWKtF
4Cneo_PPZ            YKIKYPENFFLLRGNHECANVT----RVYGFYDEC-------------KRRtNIKiWKtF
4Tmes_PPZ            YKIKYPENFFLLRGNHECANVT----RVYGFYDEC-------------KRRtNIKtWKtF
4Umay_PPZ            YKIKYPENFFLLRGNHECANVT----RVYGFYDEC-------------KRRvNIKiWKtF
4Phub_PPZ            YKIKYPENFFLLRGNHECANVT----RVYGFYDEC-------------KRRvNIKiWKtF
5Wich_PP-Z           YKLKYPENFFLLRGNHECANVT----RVYGFYDEC-------------KRRsNVKiWKqF
5Rgra_PPZ1           YKVKYPENFFLLRGNHECANVT----RVYGFYDEC-------------KRRcNIKvWKtF
5Pgra_PPZ            YKIKYPENFFLLRGNHECANVT----RVYGFYDEC-------------KRRcNIKiWKtF
6Rdel_PPZ-A          YKIKYPENFFLLRGNHECANVT----KVYGFYDEC-------------KRRlsaKmWRtF
6Rdel_PPZ-B          YKIKYPENFFLLRGNHECANVT----RVYGFYDEC-------------KRRtNtKvWKtF
Rdel_PPZ-C           YKIKYPENFFLLRGNHECANVTrgnytVYGFYDEC-------------KRRlsaKmWRtF
6Rdel_PPZ-D          YKIKYPENFFILRGNHEsANVT----KVYGFYDEC-------------KRRlNpKmWKiF
6Rdel_PPZ-E          FKIKYPENFFLLRGNHEssDVT----RiYGFYDEC-------------KRRlNMKmWKlF
6Rdel_PPZ-F          FKIKYPkNFFLLRGNHEsADVT----qiYGFYDEC-------------KRRtDVRvWKiF
6Rdel_PPZ-G          YKIKYPENFFLLRGNHECANVT----KVYGFYDEC-------------KRRlsaKmWRtF
6Ccor_PPZ            YKIKYPENFFLLRGNHECANVT----RVYGFFDEC-------------KRRasVKtWKtF
6Crev_PPZ            FKIKYPDNFFLLRGNHECAsVT----RVYGFYDEC-------------KRRcsVKvWKaF
6Spun_PPZ            -----P------------------ariiY-----s-------------wRRcNLKvWKvF
6Gpro_PPZ            YKVKYPENFFILRGNHEsAsVn----RVYGFYDEC-------------KRRcNVKvWKaF
6AMAG_PPZ-A          YKIKFPgNFFLLRGNHECAgVn----KVYGFYDEC-------------KRRatIKiWKtF
6AMAG_PPZ-B          YKIKFPgNFFLLRGNHECAgVn----KVYGFYDEC-------------KRRatIKiWKtF
6Rallo_Ppz1          YKIKYPNtFhILRGNHECAsin----RVYGFFEEC-------------KRRvNIKcWKsF
consensus PP1        yKIkyPENFFiLRGNHEcAsiN    RIYGFYDEC             KRRyniklWKtF
```

```
Consensus PPZ     ykikyPenffllrgnhecanvt    rvYgfydec              kRR nik Wk f

                 ....:...10....:...20....:...30....:...40....:...50....:...60
2YaLi_Glc7        TDCFNCLPIAAIIDEKIFaMHGGLSPELNTMEQIRRVMRPTD-----------IPDvGLL
2Mgui_PP1         TDCFNCLPIAAIIDEKIFtMHGGLSPDLNSMEQIRRVMRPTD-----------IPDvGLL
2DEHA_Glc7        TDCFNCLPIAAIIDEKIFtMHGGLSPDLNSMEQIRRVMRPTD-----------IPDvGLL
2Mfar_PP1         TDCFNCLPIAAIIDEKIFtMHGGLSPDLNSMEQIRRVMRPTD-----------IPDvGLL
2Ct_Glc7          TDCFNCLPIAAIIDEKIFtMHGGLSPDLNSMEQIRRVMRPTD-----------IPDvGLL
2Cgla_Glc7        TDCFNCLPIAAIIDEKIFcMHGGLSPDLNSMEQIRRVMRPTD-----------IPDvGLL
2Ca_Glc7          TDCFNCLPIAAIIDEKIFtMHGGLSPDLNSMEQIRRVMRPTD-----------IPDvGLL
2Cdub_PP1         TDCFNCLPIAAIIDEKIFtMHGGLSPDLNSMEQIRRVMRPTD-----------IPDvGLL
2Klac_PP1         TDCFNCLPIAAIvDEKIFcMHGGLSPDLNTMEQIRRVMRPTD-----------IPDvGLL
2Lther_Glc7       TDCFNCLPIAAIIDEKIFcMHGGLSPDLNTMEQIRRVMRPTD-----------IPDvGLL
2Ag_PP1           TDCFNCLPIAAIIDEKIFcMHGGLSPDLNTMEQIRRVMRPTD-----------IPDvGLL
2Ecym_Glc7        TDCFNCLPIAAIIDEKIFcMHGGLSPDLNTMEQIRRVMRPTD-----------IPDvGLL
2Tbla_PP1         TDCFNCLPIAAIIDEKIFcMHGGLSPDLNSMEQIRRVMRPTD-----------IPDvGLL
2Tdel_PP1         TDCFNCLPIAAIIDEKIFcMHGGLSPDLNSMEQIRRVMRPTD-----------IPDvGLL
2Sc_GLC7          TDCFNCLPIAAIIDEKIFcMHGGLSPDLNSMEQIRRVMRPTD-----------IPDvGLL
1Tmel_Glc7        TDCFNCLPIAAIIDEKIFtMHGGLSPDLNSMEQIRRVMRPTD-----------IPDcGLL
1Bcin_PP1-A       TDCFNCLPIAAIIDEKIFtMHGGLSPDLNSMEQIRRVMRPTD-----------IPDcGLL
1Nc_PP1           TDCFNCLPIAAIIDEKIFtMHGGLSPDLNSMEQIRRVMRPTD-----------IPDcGLL
1Mthe_PP1         TDCFNCLPIAAIIDEKIFtMHGGLSPDLNSMEQIRRVMRPTD-----------IPDcGLL
1Bbas_PP1         TDCFNCLPIAAIIDEKIFtMHGGLSPDLNSMEQIRRVMRPTD-----------IPDcGLL
1Anam_PP1         TDCFNCLPIAAIIDEKIFtMHGGLSPDLNSMEQIRRVMRPTD-----------IPDcGLL
1Npar_PP1         TDCFNCLPIAAIIDEKIFtMHGGLSPDLNSMEQIRRVMRPTD-----------IPDcGLL
1Bory_Glc7        TDCFNCLPIAAIIDEKIFtMHGGLSPDLNSMEQIRRVMRPTD-----------IPDcGLL
1Uree_PP1         TDCFNCLPIAAIIDEKIFtMHGGLSPDLNSMEQIRRVMRPTD-----------IPDcGLL
1Tver_PP1         TDCFNCLPIAAIIDEKIFtMHGGLSPDLNSMEQIRRVMRPTDldylllsfmhgIPDcGLL
1Anid_Glc7        TDCFNCLPIAAIIDEKIFtMHGGLSPDLNSMEQIRRVMRPTD-----------IPDcGLL
1Aory_Glc7        iDCFNCLPIAAIvEDKIFcMHGGLSPDLNSMEQIRRiMRPTD-----------IPDcGLL
1Af_PP1           TDCFNCLPIAAIIDEKIFtMHGGLSPDLNSMEQIRRVMRPTD-----------IPDcGLL
3Sp_PP1-1         TDCFNCLPIAAIIDEKIFtMHGGLSPDLNSMDQIqRiMRPTD-----------VPDtGLL
3Sp_PP1-2         TDCFNCMPvAAvIDEKIFcMHGGLSPDLNSLDQIqRiIRPTD-----------IPDtGLL
3Sj_PP1           TDCFNCLPIAAIIDEKIFtMHGGLSPDLNSMDQIqRiMRPTD-----------VPDtGLL
3Sj_Sds21         TDCFNCLPIvAvIDEKIicMHGGLSPDLrSfDQIqqiIRPTD-----------VPDsGLM
3Scot_PP1         TDCFNCLPIAAIIDEKIFtMHGGLSPDLNSMDQIqRiMRPTD-----------VPDtGLL
3Scot_Sds21       TDCFNCMPvAAIvDEKIFcMHGGLSPDLNTMEQIqRiVRPTD-----------IPDtGLL
3Scry_PP1         TDCFNCLPIAAIIDEKIFtMHGGLSPDLNSMDQIqRiMRPTD-----------VPDtGLL
3Scry_Sds21       TDCFNCMPvAAIvDEKIFcMHGGLSPDLNaMEQIqRiIRPTD-----------IPDtGLL
3Plac_PP1         TDCFNCLPIAAIIDEKIFtMHGGLSPDLNSMEQIRRVMRPTD-----------VPDtGLL
3Pjir_PP1         TDCFNCLPIAAIIDEKIFtMHGGLSPDLtSMEQIRRVMRPTD-----------VPDtGLL
3Nirr_PP1         TDCFNCLPIAAIIDEKIFtMHGGLSPDLNSMEQIRRVMRPTD-----------VPDtGLL
4GALMADRAFT_Glc7  TDCFNCLPIAAIIDEKIFtMHGGLSPDLQSMEQIRRVMRPTD-----------VPDtGLL
4PILCRDRAFT_Glc7  TDCFNCLPIAAIIDEKIFtMHGGLSPDLQSMEQIRRVMRPTD-----------VPDtGLL
4Shir_Glc7        TDCFNCLPIAAIIDEKIFtMHGGLSPDLQSMEQIRRVMRPTD-----------VPDtGLL
4Sver_PP1         TDCFNCLPIAAIIDEKIFtMHGGLSPDLQSMEQIRRVMRPTD-----------VPDtGLL
4Pgig_PP1         TDCFNCLPIAAIIDEKIFtMHGGLSPDLQSMEQIRRVMRPTD-----------VPDtGLL
4Bbot_PP1         TDCFNCLPIAAIIDEKIFtMHGGLSPDLQSMEQIRRVMRPTD-----------VPDtGLL
4Cvis_PP1-A       TDCFNCLPIAAIIDEKIFtMHGGLSPDLQSMEQIRRVMRPTD-----------VPDtGLL
4Cvis_PP1-B       TDCFNCLPIAALIDDKIFcMHGGLSPDLQSMEQvRRVMRPTD-----------LPDeGLL
4Cgat_PP1         TDCFNCLPIAAIIDEKIFtMHGGLSPDLQSMEQIRRVMRPTD-----------VPDtGLL
4Cneo_PP1         TDCFNCLPIAAIIDEKIFtMHGGLSPDLQSMEQIRRVMRPTD-----------VPDtGLL
4Tmes_PP1         TDCFNCLPIAAIIDEKIFtMHGGLSPDLQTMEQIRRVMRPTD-----------VPDtGLL
4Umay_Glc7        TDCFNCLPIAAIIDEKIFtMHGGLSPDLQSMEQIRRVMRPTD-----------VPDtGLL
4Phub_PP1         TDCFNCLPIAAIIDEKIFtMHGGLSPDLQSMEQIRRVMRPTD-----------VPDtGLL
5Wich_Glc7        TDCFNCLPIAAIIDEKIFtMHGGLSPDLQSMDQIRRVMRPTD-----------VPDtGLL
5Rgra_PP1         TDCFNCLPIAAIIDEKIFtMHGGLSPDLQSMEQIRRVMRPTD-----------VPDtGLL
5Pgra_PP1         TDCFNCLPIAAIIDEKIFtMHGGLSPDLQSMEQIRRVMRPTD-----------VPDtGLL
6Rdel_PP1         TDCFNCLPIAALvDEKIFcMHGGLSPDLQSfEQIRRVMRPTD-----------VPDtGLL
6Ccor_PP1         TDCFNCLPIAAIIDEKIFcMHGGLSPDLeTfDQIRRiMRPTD-----------VPDmGLL
6Crev_PP1         TDCFNCLPIAAIIDEKIFsMHGGLSPDLiSMDQIKRiMRPTD-----------VPDtGLL
6Spun_PP1         TDCFNCLPIAAIIDEKIFtMHGGLSPDLQSMDQIRRVMRPTD-----------VPDtGLL
6Gpro_PP1-A       TDCFNCLPIAAIIDEKIFtMHGGLSPDLQSMEQIRRVMRPTD-----------VPDtGLL
6Gpro_PP1-B       TDCFNCLPvAALIEDRIllMHGGLSPDLQnLDQIRRiMRPTE-----------VPDaGLL
6AMAG_PP1-A       TDCFNCLPIAAIIDEKIFtMHGGLSPDLQSMEQIRRVMRPTD-----------VPDtGLL
6Rallo_PP1        TDCFNCLPIAAIIDEKIFtMHGGLSPELQSMEQIRRVMRPTD-----------VPDtGLL
```

```
                    ....:...10....:...20....:...30....:...40....:...50....:...60
2YALI_Ppz1          VDtFNtLPIAaVVAqKIFCVHGGLSPsLssMsEIRsIvRPTD-----------VPDYGLI
2Mgui_Ppz1          IDtFNtLPIAaIVAgKIFCVHGGLSPvLnsMDEIRtIaRPTD-----------VPDYGLL
2DEHA_Ppz1          IDtFNtLPIAaIVAgKIFCVHGGLSPvLnsMEEIRnIaRPTD-----------VPDFGLL
2Mfar_Ppz           IDtFNtLPIAaIVArKIFCVHGGLSPvLnsMDEIRnIaRPTD-----------VPDFGLL
2Ct_Ppz1            IDtFNtLPIAaIVAgKIFCVHGGLSPvLnsMDEIRnIaRPTD-----------VPDFGLL
2Cgla_Ppz1          IDtFNtLPLtaIVAgKIFCVHGGLSPvLntMDEIRnMsRPTD-----------VPDFGLI
2Cgla_Ppz2          IDtFNtLPLAaIVtgKIFCVHGGLSPvLnsMDEIRhVsRPTD-----------VPDFGLI
2Ca_Ppz1            IDtFNtLPIAaIVAgKIFCVHGGLSPvLnsMDEIRnIaRPTD-----------VPDFGLL
2Cdub_Ppz           IDtFNtLPIAaIVAgKIFCVHGGLSPvLnsMDEIRnIaRPTD-----------VPDFGLL
2Klac_PpzA          IDtFNaLPLAaIVAgKIFCVHGGLSPvLnsMDEIRhVsRPTD-----------VPDFGLI
2Klac_PpzB          IDtFNaLPLAaIVAgKIFCVHGGLSPvLnsMDEIRhVsRPTD-----------VPDFGLI
2Lther_Ppz1         IDtFNtLPLAaIVAgKIFCVHGGLSPvLnsMDEIRhVsRPTD-----------VPDFGLI
2Ag_Ppz             IDtFNtLPLAaIVAgKIFCVHGGLSPvLnsMDEIRhVsRPTD-----------VPDFGLI
2Ecym_Ppz           IDtFNtLPLAaIVAgKIFCVHGGLSPvLnsMDEIRhVsRPTD-----------VPDFGLI
2Tbla_PpzA          IDtFNtLPLAaIVAgKIFCVHGGLSPiLnsMDEIRhVsRPTD-----------VPDFGLI
2Tdel_PpzA          VDtFNtLPLAaIVAgKfFCVHGGLSPvLnsMDEIRhVsRPTD-----------VPDFGLI
2Sc_PPZ1            IDtFNtLPLAaIVAgKIFCVHGGLSPvLnsMDEIRhVvRPTD-----------VPDFGLI
2Sc_PPZ2            VDtFNtLPLAaIVtgKIFCVHGGLSPvLnsMDEIRhVsRPTD-----------VPDFGLI
1Tmel_Ppz1          IDtFNcLPIAsIVAgKIFCVHGGLSPsLshMDDIRqIaRPTD-----------VPDYGLL
1Bcin_Ppz           VDvFNcLPIAaIVAgKIFCVHGGLSPaLshMDDIRnIaRPTD-----------VPDYGLL
1Nc_PP-Z            VDtFNcLPIAaIVAgKIFCVHGGLSPaLghMDDIRnIaRPTD-----------VPDYGLL
1Mthe_Ppz           IDtFNcLPIAaIVAaKIFCVHGGLSPaLshMDDIRnIaRPTD-----------VPDYGLL
1Bbas_Ppz           IDcFNtLPIAaIVAgKIFCVHGGLSPaLshMDDIRnIaRPTD-----------VPDYGLL
1Anam_Ppz           VDtFNcLPIAsVVAnKIFCVHGGLSPsLshMDDIRnIaRPTD-----------VPDYGLL
1Npar_Ppz1          VDtFNtLPIAaIVAgKIFCVHGGLSPsLshMDDIRqIaRPTD-----------VPDYGLL
1Bory_Ppz1          VDtFNtLPIAaIVAqKIFCVHGGLSPsLshMDDIRqIaRPTD-----------VPDYGLL
1Uree_Ppz           VDtFNcLPIAsIVAgKIFCVHGGLSPsLshMDDIRgIaRPTD-----------VPDYGLL
1Tver_Ppz           VDtFNcLPIAaIVAgKIFCVHGGLSPsLshMDDIRgIaRPTD-----------VPDYGLL
1Anid_Ppz1          VDtFNcLPIAatVAgKIFCVHGGLSPsLshMDDIRnIaRPTD-----------VPDYGLL
1Aory_Ppz1          IDtFNcLPIAsIVAgKIFCVHGGLSPsLshMDDIRgIaRPTD-----------VPDYGLL
1Af_PPZ             IDtFNcLPIAatVAgKIFCVHGGLSPsLshMDDIRgIaRPTD-----------VPDYGLL
3Sp_PPZ             INtFNcLPIAsVVAgKIFCVHGGLSPsLshMDDIReIpRPTD-----------VPDYGLL
3Sj_Pzh1            IDtFNcLPIAsVVAgKIFCVHGGLSPcLnsMNDIReIvRPTD-----------VPDcGLL
3Soct_Ppz           INtFNcLPIAsVVAgKIFCVHGGLSPaLssMDDIKeItRPTD-----------VPDYGLL
3Scry_Ppz           INtFNcLPIAsVVAgKIFCVHGGLSPaLssMDDIKeItRPTD-----------VPDYGLL
3Plac_Ppz           IDaFNtLPIAaVVAnKIFCVHGGLSPsLahMDEIRaItRPTD-----------VPDYGLL
3Pjir_Ppz-A         IDaFNtLPIAaVVAnKIFCVHGGLSPsLahMDEIRaItRPTD-----------VPDYGLL
3Nirr_PP-Z          IDtFNcLPIAsIVAnKIFCVHGGLSPsLhaMsEIHtInRPTD-----------VPDYGLL
4GALMADRAFT_Ppz1    IDvFNcLPIAaIVAsKIFCVHGGLSPsLhtMEDIKrIqRPTD-----------VPDYGLL
4PILCRDRAFT_Ppz1    IDvFNcLPIAaIVAsKIFCVHGGLSPsLhsMDDIKrIqRPTD-----------VPDYGLL
4Shir_Ppz           IDvFNcLPIAaIVAsKIFCVHGGLSPsLqsMDDIKaLqRPTD-----------VPDYGLL
4Sver_PPZ           IDvFNcLPIAsIVAsKIFCVHGGLSPnLqsMEDIKrIaRPTD-----------VPDYGLL
4Pgig_PPZ           IDvFNcLPIAaIVAsKIFCVHGGLSPsLhhMDDIKrIqRPTD-----------VPDYGLL
4Bbot_PPZ           IDvFNcLPIAaVVAsKIFCVHGGLSPsMhsMEDIKrInRPTD-----------VPDFGLL
4Cvis_PPZ           IDvFNaLPIAsIVAsKIFCVHGGLSPsLnnMDDIRrIaRPTD-----------VPDYGLL
4Cgat_PPZ           IDvFNtLPIAsIVAsKIFCVHGGLSPsLksMDDIRrIqRPTD-----------VPDYGLL
4Cneo_PPZ           IDvFNtLPIAsIVAsKIFCVHGGLSPsLksMDDIRrIqRPTD-----------VPDYGLL
4Tmes_PPZ           IDvFNtLPIAsIVAsKIFCVHGGLSPsLksMDDIRrIqRPTD-----------VPDFGLL
4Umay_PPZ           IDvFNtLPIAaVVAsKIFCVHGGLSPsLsnMDDIRrIeRPTD-----------VPDYGLL
4Phub_PPZ           IDvFNtLPIAaVVAsKIFCVHGGLSPsLsnMDDIRrIeRPTD-----------VPDYGLL
5Wich_PP-Z          VDvFNsfPIAaVVAsKIFCVHGGLSPnLttMDDIRrIqRPlD-----------VPDYGLL
5Rgra_PPZ1          IDvFNcLPIAaVVAsKIFCVHGGLSPsLssMDDIRrIsRPTD-----------VPDYGLL
5Pgra_PPZ           IDvFNcLPIsaVVAsKIFCVHGGLSPsLstMDDIRrIqRPTD-----------VPDFGLL
6Rdel_PPZ-A         VDvFNtLPIAaLVAgKIFCVHGGLSPsLhsMDEIRnIqRPTD-----------VPDYGLL
6Rdel_PPZ-B         IDaFNtLPIAaLVAsKIFCVHGGLSPsLhsMDDvRnImRPTD-----------VPEYGLL
Rdel_PPZ-C          VDvFNtLPIAaLVAgKIFCVHGGLSPsLhsMDEIRnIqRPTD-----------VPDYGLL
6Rdel_PPZ-D         VDvFNtLPIAaLVAgKIFCVHGGLSPsLssMDDIRnIqRPTD-----------VPDYGLL
6Rdel_PPZ-E         cDvFNtMPIAaIiGnRIFCVHGGLSPfMksMDEInaLpRPTE-----------VPEYGLL
6Rdel_PPZ-F         cDvFNaMPIAaIVGgKIFCVHGGLSPwMksMDqIRqLpRPTE-----------VPDqGLL
6Rdel_PPZ-G         VDvFNtLPIAaLVAgKIFCVHGGLSPsLhsMDEIRnIqRPTD-----------VPDYGLL
6Ccor_PPZ           VsvFNcLPVAaVVAnKIFCVHGGLSPsLhdMDDIRqIeRPSD-----------VPDYGLL
6Crev_PPZ           VNtFNtLPVAaVVAnKIFCVHGGLSPdLlnMEqIRqLpRPcD-----------VPDhGIL
6Spun_PPZ           tDvFNcLPIAaLVAgKIFCVHGGLSPsLhsMDDIRaVaRPSD-----------VPDFGLL
6Gpro_PPZ           tDvFNcLPVAaIVAgKIFCVHGGLSPnLntMDDIRnLgRPTD-----------VPDvGIL
6AMAG_PPZ-A         IDvFDcLPIAaIVAgKIFCVHGGLSPsLdtMEEvRnLpRPTD-----------VPDYGLL
6AMAG_PPZ-B         IDvFDcLPIAaIVAgKIFCVHGGLSPsLdtMEDvRnLpRPTD-----------VPDYGLL
6Rallo_Ppz1         tDvFNvIPVAaIVAeKIFCVHGGLSPgLysMkqIemIkRPTD-----------VPDFGLL
consensus PP1       tDCFNClPiaAiidekIf MHGGLSPdLnsmeQirrvmRPTd            iPD GLl
```

```
consensus PPZ      id Fn lPia iva kiFCVHGGLSP l  Mddir i RPtd          VPdyGll

                   ....:...10....:...20....:...30....:...40....:...50....:...60
2YaLi_Glc7         CDLLWSDPDKDITGWnENDRGVSFTFGPDVVlRFLQKHDMDLICRAHQVVEDGYEFFsKR
2Mgui_PP1          CDLLWSDPDKDITGWSENDRGVSFTFGPDVVSRFLQKHDMDLICRAHQVVEDGYEFFsKR
2DEHA_Glc7         CDLLWSDPDKDITGWSENDRGVSFTFGPDVVSRFLQKHDMDLICRAHQVVEDGYEFFsKR
2Mfar_PP1          CDLLWSDPDKDITGWSENDRGVSFTFGPDVVSRFLQKHDMDLvCRAHQVVEDGYEFFsKR
2Ct_Glc7           CDLLWSDPDKDITGWSENDRGVSFTFGPDVVSRFLQKHDMDLICRAHQVVEDGYEFFsKR
2Cgla_Glc7         CDLLWSDPDKDIvGWSENDRGVSFTFGPDVVnRFLQKqDMELICRAHQVVEDGYEFFsKR
2Ca_Glc7           CDLLWSDPDKDITGWSENDRGVSFTFGPDVVSRFLQKHDMDLICRAHQVVEDGYEFFsKR
2Cdub_PP1          CDLLWSDPDKDITGWSENDRGVSFTFGPDVVSRFLQKHDMDLICRAHQVVEDGYEFFsKR
2Klac_PP1          CDLLWSDPDKDIvGWSENDRGVSFTFGPDVVSRFLQKqDMELICRAHQVVEDGYEFFsKR
2Lther_Glc7        CDLLWSDPDKDIvGWSENDRGVSFTFGPDVVSRFLQKqDMELICRAHQVVEDGYEFFsKR
2Ag_PP1            CDLLWSDPDKDIvGWSENDRGVSFTFGPDVVSRFLQKqDMELICRAHQVVEDGYEFFsKR
2Ecym_Glc7         CDLLWSDPDKDIvGWSENDRGVSFTFGPDVVSRFLQKqDMELICRAHQVVEDGYEFFsKR
2Tbla_PP1          CDLLWSDPDKDIvGWSENDRGVSFTFGPDVVnRFLQKqDMELICRAHQVVEDGYEFFsKR
2Tdel_PP1          CDLLWSDPDKDIvGWSENDRGVSFTFGPDVVnRFLQKqDMELICRAHQVVEDGYEFFsKR
2Sc_GLC7           CDLLWSDPDKDIvGWSENDRGVSFTFGPDVVnRFLQKqDMELICRAHQVVEDGYEFFsKR
1Tmel_Glc7         CDLLWSDPDKDITGWSENDRGVSFTFGPDVVSRFLQKHDMDLICRAHQVVEDGYEFFsKR
1Bcin_PP1-A        CDLLWSDPDKDITGWSENDRGVSFTFGPDVVSRFLQKHDMDLICRAHQVVEDGYEFFsKR
1Nc_PP1            CDLLWSDPDKDITGWSENDRGVSFTFGPDVVSRFLQKHDMDLICRAHQVVEDGYEFFsKR
1Mthe_PP1          CDLLWSDPDKDITGWSENDRGVSFTFGPDVVSRFLQKHDMDLICRAHQVVEDGYEFFsKR
1Bbas_PP1          CDLLWSDPDKDITGWSENDRGVSFTFGPDVVSRFLQKHDMDLICRAHQVVEDGYEFFsKR
1Anam_PP1          CDLLWSDPDKDITGWSENDRGVSFTFGPDVVSRFLQKHDMDLICRAHQVVEDGYEFFsKR
1Npar_PP1          CDLLWSDPDKDITGWSENDRGVSFTFGPDVVSRFLQKHDMDLICRAHQVVEDGYEFFsKR
1Bory_Glc7         CDLLWSDPDKDITGWSENDRGVSFTFGPDVVSRFLQKHDMDLICRAHQVVEDGYEFFsKR
1Uree_PP1          CDLLWSDPDKDITGWSENDRGVSFTFGPDVVSRFLQKHDMDLICRAHQVVEDGYEFFsKR
1Tver_PP1          CDLLWSDPDKDITGWSENDRGVSFTFGPDVVSRFLQKHDMDLICRAHQVVEDGYEFFsKR
1Anid_Glc7         CDLLWSDPDKDITGWSENDRGVSFTFGPDVVSRFLQKHDMDLICRAHQVVEDGYEFFsKR
1Aory_Glc7         CDLLWaDPEKEITGWgENDRGVSFTFGPDVVhRFvQKHDIDLICRAHQcVEDGYEFFaKR
1Af_PP1            CDLLWSDPDKDITGWSENDRGVSFTFGPDVVSRFLQKHDMDLICRAHQVVEDGYEFFsKR
3Sp_PP1-1          CDLLWSDPDKDLTGWgDNDRGVSFTFGPDVVSRFLhKHDMDLvCRAHQVVEDGYEFFsKR
3Sp_PP1-2          CDLvWSDPEKDLTGWgENDRGVSYTFGaDVVSRFLQKHDLDLICRAHQVVEDGYEFFgKR
3Sj_PP1            CDLLWSDPDKDLTGWgENDRGVSFTFGPDVVSRFLhKHDMDLvCRAHQVVEDGYEFFsKR
3Sj_Sds21          CDLLWSDPEegvTGWgENDRGiSYvFGaDVVSRFLQKHDMDLvCRAHQVVEDGYEFFcKR
3Scot_PP1          CDLLWSDPDnDLTGWgDNDRGVSFTFGPDVVSRFLhKHDMDLvCRAHQVVEDGYQFFsKR
3Scot_Sds21        CDLLWSDPEKDvkGWgDNDRGVSYTFGaDVVnRFLQKqDMDLvCRAHQVVEDGYEFFaKR
3Scry_PP1          CDLLWSDPDKDLTGWgDNDRGVSFTFGPDVVSRFLhKHDMDLvCRAHQVVEDGYEFFsKR
3Scry_Sds21        CDLLWSDPEKDvTGWgDNDRGVSYTFGaDVVnRFLQKHDMDLICRAHQVVEDGYEFFaKR
3Plac_PP1          CDLLWSDPDKDITGWSENDRGVSFTFGPDVVSRFLQKHDMDLICRAHQVVEDGYEFFsKR
3Pjir_PP1          CDLLWSDPDKDITGWSENDRGVSFTFGPDVVSRFLQKHDMDLICRAHQVVEDGYEFFsKR
3Nirr_PP1          CDLLWSDPDKEITGWSENDRGVSFTFGPDVVSRFLaKHDMDLICRAHQVVEDGYEFFsKR
4GALMADRAFT_Glc7   CDLLWSDPDKDITGWSENDRGVSFTFGPDVVSRFLQKHDMDLICRAHQVVEDGYEFFaKR
4PILCRDRAFT_Glc7   CDLLWSDPDKDITGWSENDRGVSFTFGPDVVTRFLQKHDMDLICRAHQVVEDGYEFFaKR
4Shir_Glc7         CDLLWSDPDKDITGWSENDRGVSFTFGPDVVSRFLQKHDMDLICRAHQVVEDGYEFFaKR
4Sver_PP1          CDLLWSDPDKDITGWSENDRGVSFTFGPDVVSRFLQKHDMDLICRAHQVVEDGYEFFaKR
4Pgig_PP1          CDLLWSDPDKEITGWSENDRGVSFTFGPDVVSRFLQKHDMDLICRAHQVVEDGYEFFaKR
4Bbot_PP1          CDLLWSDPDKDISGWSENDRGVSFTFGPDVVSRFLQKHDMDLICRAHQVVEDGYEFFaKR
4Cvis_PP1-A        CDLLWSDPDKDITGWSENDRGVSFTFGPDVVSRFLQKHDMDLICRAHQVVEDGYEFFaKR
4Cvis_PP1-B        CDLLWaDPDKEvTGWSpNDRGVSYTFGaDVVlKFLdKHDLqLIvRAHQVVEDGYEFFaKR
4Cgat_PP1          CDLLWSDPDKDITGWSENDRGVSFTFGPDVVSRFLQKHDMDLICRAHQVVEDGYEFFaKR
4Cneo_PP1          CDLLWSDPDKDITGWSENDRGVSFTFGPDVVSRFLQKHDMDLICRAHQVVEDGYEFFaKR
4Tmes_PP1          CDLLWSDPDKDITGWSENDRGVSFTFGPDVVSRFLQKHDMDLICRAHQVVEDGYEFFaKR
4Umay_Glc7         CDLLWSDPDKDISGWSENDRGVSFTFGPDVVSRFLQKHDMDLICRAHQVVEDGYEFFaKR
4Phub_PP1          CDLLWSDPDKDISGWSENDRGVSFTFGPDVVSRFLQKHDMDLICRAHQVVEDGYEFFaKR
5Wich_Glc7         CDLLWSDPDKDISGWSENDRGVSFTFGPDVVTRFLQKHDMDLICRAHQVVEDGYEFFaKR
5Rgra_PP1          CDLLWSDPDKDITGWSENDRGVSFTFGPDVVSRFLQKHDMDLICRAHQVVEDGYEFFaKR
5Pgra_PP1          CDLLWSDPDKDITGWSENDRGVSFTFGPDVVTRFLQKHDMDLICRAHQVVEDGYEFFaKR
6Rdel_PP1          CDLLWSDPDKDIvGWSENDRGVSFTFGPDiVSKFLQKHDIDLICRAHQVVEDGYEFFsKR
6Ccor_PP1          CDLLWSDPDKDvTGWnENDRGVSYTFGPDVVTKFLkKHDMDLICRAHQVVEDGYEFFaKR
6Crev_PP1          CDLLWSDPDKEIqGWSENDRGVSFTFGPDiVfKFLQKHDLDLvCRAHQVVEDGYEFFaKR
6Spun_PP1          CDLLWSDPDKDITGWSENDRGVSFTFGPDVVTRFLQKHDLDLICRAHQVVEDGYEFFaKR
6Gpro_PP1-A        CDLLWSDPDKDITGWSENDRGVSFTFGPDVVSRFLQKHDLDLICRAHQVVEDGYEFFaKR
6Gpro_PP1-B        CDLLWSDPEaDtTGWnENDRGaSFTFGPDVVaRFLrKHDLDLICRAHQVVEDGYEFFaKK
6AMAG_PP1-A        CDLLWSDPDKDITGWSENDRGVSFTFGPDVVTRFLQKHDMDLICRAHQVVEDGYEFFaKR
6Rallo_PP1         CDLLWSDPDKEvTGWSENDRGVSFvFGPDVVTRFLQKHDMDLICRAHQVVEDGYEFYaKR
```

```
                    ....:....10....:....20....:....30....:....40....:....50....:....60
2YALI_Ppz1          NDLLWSDPaDtqnEWEDNERGVSYcFsKvaInkFLtKfsfDLVCRAHMVVEDGYEFFnDR
2Mgui_Ppz1          NDLLWSDPaDtqnEWEDNERGVSYvFsRtaInkFLqKfgfDLVCRAHMVVEDGYEFFnDR
2DEHA_Ppz1          NDLLWSDPaDtmnEWEDNERGVSYvFsRvaInkFLqKfgfDLVCRAHMVVEDGYEFFnDR
2Mfar_Ppz           NDLLWSDPaDtinEWEDNERGVSYvFsKvaInkFLsKfgfDLVCRAHMVVEDGYEFFnDR
2Ct_Ppz1            NDLLWSDPaDtinEWEDNERGVSYvFsKvaInkFLsKfnfDLVCRAHMVVEDGYEFFnDR
2Cgla_Ppz1          NDLLWSDPiDspnEWEDNDRGVSYcYnKvaInkFInKfnfDLVCRAHMVVEDGYEFFnDR
2Cgla_Ppz2          NDLLWSDPtDspnEWEDNERGVSYcYnKvaInkFLnKfgfDLVCRAHMVVEDGYEFFnDR
2Ca_Ppz1            NDLLWSDPaDtinEWEDNERGVSYvFsKvaInkFLsKfnfDLVCRAHMVVEDGYEFFnDR
2Cdub_Ppz           NDLLWSDPaDtinEWEDNERGVSYvFsKvaInkFLsKfnfDLVCRAHMVVEDGYEFFnDR
2Klac_PpzA          NDLLWSDPtDspnEWEDNERGVSYcYnKvaInkFLnKfgfDLVCRAHMVVEDGYEFFnDR
2Klac_PpzB          NDLLWSDPtDspnEWEDNERGVSYcYnKvaInkFLnKfgfDLVCRAHMVVEDGYEFFnDR
2Lther_Ppz1         NDLLWSDPtDsphEWEDNERGVSYcYnKvaInkFLnKfgfDLVCRAHMVVEDGYEFFnDR
2Ag_Ppz             NDLLWSDPtDspnEWEDNERGVSYcYnKvaInkFLnKfgfDLVCRAHMVVEDGYEFFnDR
2Ecym_Ppz           NDLLWSDPtDspnEWEDNERGVSYcYnKvaInkFLnKfgfDLVCRAHMVVEDGYEFFnDR
2Tbla_PpzA          NDLLWSDPtDspnEWEDNERGVSYcYnKvaInkFLnRfgfDLVCRAHMVVEDGYEFFnDR
2Tdel_PpzA          NDLLWSDPtDspnEWEDNERGVSYcYnKvaInkFLnKfgfDLVCRAHMVVEDGYEFFnDR
2Sc_PPZ1            NDLLWSDPtDspnEWEDNERGVSYcYnKvaInkFLnKfgfDLVCRAHMVVEDGYEFFnDR
2Sc_PPZ2            NDLLWSDPtDssnEWEDNERGVSFcYnKvaInkFLnKfgfDLVCRAHMVVEDGYEFFnDR
1Tmel_Ppz1          sDLLWSDPaDmqtDWEENERGVSYcFgKkvIleFLaRhdfDLVCRAHMVVEDGYEFFnDR
1Bcin_Ppz           NDLLWSDPaDqeaDWEqsERGVSYsFgKkvIqeFLlRhdfDLVCRAHMVVEDGYEFFegR
1Nc_PP-Z            NDLLWSDPaDmdqDWEaNERGVSYcFgKkvIsdFLathdfDLVCRAHMVVEDGYEFYtDR
1Mthe_Ppz           NDLLWSDPaDmdqDWEaNERGVSYcFgKkvItdFLagndfDLVCRAHMVVEDGYEFFtDR
1Bbas_Ppz           NDLLWSDPaDmeqDWEaNERGVSYcFgKrvItdFLavhdfDLICRAHMVVEDGYEFFnDR
1Anam_Ppz           NDLLWSDPaDmenDWEaNERGVSYcFgKkvImeFLaKhdfDLVCRAHMVVEDGYEFFtDR
1Npar_Ppz1          NDLLWSDPaDmdnDWEaNERGVSYcFgKkvImeFLqRhdfDLVCRAHMVVEDGYEFFnDR
1Bory_Ppz1          NDLLWSDPaDmenDWEsNERGVSYcFgKkvImeFLaRhdfDLVCRAHMVVEDGYEFFtDR
1Uree_Ppz           NDLLWSDPaDmeeDWEpNERGVSYcFgKkvImdFLqRhdfDLVCRAHMVVEDGYEFFnDR
1Tver_Ppz           NDLLWSDPaEmeeDWEpNERGVSYcFgKkvImeFLqRhdfDLVCRAHMVVEDGYEFFnDR
1Anid_Ppz1          NDLLWSDPaDmdeDWEpNERGVSYcFnKnvImnFLqRhdfDLVCRAHMVVEDGYEFYqDR
1Aory_Ppz1          NDLLWSDPaDmeeDWEpNERGVSYcFgKkvImnFLqRhdfDLVCRAHMVVEDGYEFYqDR
1Af_PPZ             NDLLWSDPaDmeeDWEpNERGVSYcFgKkvImnFLqRhdfDLVCRAHMVVEDGYEFYqDR
3Sp_PPZ             NDLLWSDPaDtenDWEDNERGVSFvFnKnvIrqFLaKhdfDLICRAHMVVEDGYEFFnDR
3Sj_Pzh1            NDLLWSDPaDnekDWEDNERGVSYcFnKnvIrqFLaKfefDLICRAHMVVEDGYEFFnDR
3Soct_Ppz           NDLLWSDPaDmenDWEDNERGVSFvFnKniIrqFLaRhdfDLICRAHMVVEDGYEFFnER
3Scry_Ppz           NDLLWSDPaDlenDWEDNERGVSFvFnKnvIrqFLaRhdfDLICRAHMVVEDGYEFFnER
3Plac_Ppz           NDLLWSDPaDihnDWEDsERGVSYcFsKnvIhqFLaKhdfDLVCRAHMVVEDGYEFFnDR
3Pjir_Ppz-A         NDLLWSDPaDihnDWEDsERGVSYcFsKnvIhqFLaKhdfDLVCRAHMVVEDGYEFFnDR
3Nirr_PP-Z          NDLLWSDPaEtkdDWEpNERGVSYcFgRnvIdsFLrRfdfDLVCRAHMVVEDGYEFFnER
4GALMADRAFT_Ppz1    NDLLWSDPsDtalDWEDNERGVSYcFgKaiIneFLvRydmDLICRAHMVVEDGYEFWnDR
4PILCRDRAFT_Ppz1    NDLLWSDPsDtalDWEDNERGVSYcFgKaiIndFLvRydmDLICRAHMVVEDGYEFWnDR
4Shir_Ppz           NDLLWSDPsDtavEWEDNERGVSYcFgKaiIndFLvRydmDLICRAHMVVEDGYEFWnDR
4Sver_PPZ           NDLLWSDPsEtaiDWEDNERGVSYcFgKsvIneFLaKwdmDLVCRAHMVVEDGYEFWnDR
4Pgig_PPZ           NDLLWSDPsDtaqDWEDNERGVSYcFgKsiIndFLvRydmDLICRAHMVVEDGYEFWnDR
4Bbot_PPZ           NDLLWSDPsDtatEWEDNERGVSYcFgKsiIneFLgRydmDLICRAHMVVEDGYEFWnDR
4Cvis_PPZ           NDLLWSDPsDtaqDWEDNERGVSFcFgKrvIqdFLeRydmDLVCRAHMVVEDGYEFWnDR
4Cgat_PPZ           NDLvWSDPsDtalDWEDNERGVSFcYgKsvInaFLathdmDLICRAHMVVEDGYEFYnDR
4Cneo_PPZ           NDLvWSDPsDtalDWEDNERGVSFcYgKsvInaFLathdmDLICRAHMVVEDGYEFYnDR
4Tmes_PPZ           NDLvWSDPsDttmDWEDNERGVSYcYgKsvInaFLathdmDLICRAHMVVEDGYEFYnDR
4Umay_PPZ           NDLLWSDPsDtalDWEDNERGVSYcFgKavIqqFLaqydfDLICRAHMVVEDGYEFWnER
4Phub_PPZ           NDLLWSDPsDtalDWEDNERGVSYcFgKavIqqFLaqydfDLICRAHMVVEDGYEFWnER
5Wich_PP-Z          NDLLWSDPsDtafDWEENERGVSYcFgKsvIndFLsRyefDLICRAHMVVEDGYEFWnDR
5Rgra_PPZ1          NDLLWSDPsDtalDWEDNERGVSYcFgKtvIgtFLaRydfDLICRAHMVVEDGYEFWcDR
5Pgra_PPZ           NDLLWSDPsDtatDWEDNERGVSYcFgKsvIaeFLaRydfDLICRAHMVVEDGYEFWnER
6Rdel_PPZ-A         NDLLWSDPaDidgDWEDNERGVSYvFgKkvIneFLsKfdlDLVCRAHMVVEDGYEFFgnR
6Rdel_PPZ-B         NDLvWSDPsDtavEWEDNERGVSYcFgKkivneFLaKhdlDLVCRAHMVVEDGYQFFnER
Rdel_PPZ-C          NDLLWSDPaDidgDWEDNERGVSYvFgKkvIneFLsKfdlDLVCRAHMVVEDGYEFFgnR
6Rdel_PPZ-D         NDLLWaDPaDitanWEENERGVSYvFgKkvIndFLsKfdlDLVCRAHMVVEDGYQFFgDR
6Rdel_PPZ-E         NDLLWSDPsDlsdDWsENDRGVSvcFgRnnvneFLqRfdlDLICRAHMVVEDGYEFFsDR
6Rdel_PPZ-F         NDLLWSDPsDlssDWsENDRGVSstYgKkmIdll-----svLIC-plkVVEgGYEFFnnR
6Rdel_PPZ-G         NDLLWSDPaDidgDWEDNERGVSYvFgKkvIneFLsKfdlDLVCRAHMVVEDGYEFFgnR
6Ccor_PPZ           NDLLWSDPsDttaEWEDNERGVSYcFgKqiIseFLqKyefDLVCRAHMVVEDGYEFFngR
6Crev_PPZ           NDLLWSDPsDtaaDWEDNERGVSYcFgKsiIteFLrRmdfDLICRAHMVVEDGYEFFhgR
6Spun_PPZ           NDLLWSDPsDlatDWEDNERGVSYcFsKtivseFLsRhdlDLVCRAHMVVEEGYEFFnnR
6Gpro_PPZ           NDLLWSDPsEtavEWEDsERGVSYcFgKqvvqeFLqKndfDLVCRAHMiVEDGYEFFgnR
6AMAG_PPZ-A         NDLLWSDPsEtaiDWEDNERGVSYcFgasvIqqFLlKhdfDLVaRAHMVVEDGYEFFgDR
6AMAG_PPZ-B         NDLLWSDPsEtaiDWEDNERGVSYcFgasvIqqFLlKhdfDLVaRAHMVVEDGYEFFgDR
6Rallo_Ppz1         NDLLWaDPnEnvaEWEENDRGVSYlFgKsiIdqFLqKhdlDLICRAHtVVEDGYEFFnDR
```

```
consensus PP1    CDLlWsDPdkditGWseNDRGvSftFGpDvVsrFlqKhDmdLicRAHQvVEDGYeFf Kr
consensus PPZ    nDLlWsDP d   dWedneRGVSy f k  i  fl k   dLvcrahmvVEdGYeFf dR
                 ....:...10...:...20...:...30...:...40...:...50...:...60
2YaLi_Glc7       hLVTLFSAPNYCGEFDNAGA---MMSVDESLLCSFQILKPAEKKqKfvys-gmg--ggrp
2Mgui_PP1        QLVTLFSAPNYCGEFDNAGA---MMSVDESLLCSFQILKPADKKpRypa--n-nvgnnrp
2DEHA_Glc7       QLVTLFSAPNYCGEFDNAGA---MMSVDESLLCSFQILKPADKKpRypqs-n-nisnnrp
2Mfar_PP1        QLVTLFSAPNYCGEFDNAGA---MMSVDESLLCSFQILKPADKKpRypqa-n-nignnrp
2Ct_Glc7         QLVTLFSAPNYCGEFDNAGA---MMSVDESLLCSFQILKPADKKpRyapa-nvann--rp
2Cgla_Glc7       QLVTLFSAPNYCGEFDNAGA---MMSVDESLLCSFQILKPAqKslprqqagr--------
2Ca_Glc7         QLVTLFSAPNYCGEFDNAGA---MMSVDESLLCSFQILKPADKKpRyapa-nvann--rp
2Cdub_PP1        QLVTLFSAPNYCGEFDNAGA---MMSVDESLLCSFQILKPADKKpRyapa-nvann--rp
2Klac_PP1        QLVTLFSAPNYCGEFDNAGA---MMSVDESLLCSFQILKPAsKllernvvrr--------
2Lther_Glc7      QLVTLFSAPNYCGEFDNAGA---MMSVDESLLCSFQILKPAqKtlprpqgkk--------
2Ag_PP1          QLVTLFSAPNYCGEFDNAGA---MMSVDESLLCSFQILKPAvKpsagrgqpk--------
2Ecym_Glc7       QLVTLFSAPNYCGEFDNAGA---MMSVDESLLCSFQILKPAvKpsagrgkpk--------
2Tbla_PP1        QLVTLFSAPNYCGEFDNAGA---MMSVDESLLCSFQILKPAqKslprqqgkk--------
2Tdel_PP1        QLVTLFSAPNYCGEFDNAGA---MMSVDESLLCSFQILKPAqKslprqqggr--------
2Sc_GLC7         QLVTLFSAPNYCGEFDNAGA---MMSVDESLLCSFQILKPAqKslprqaggr--------
1Tmel_Glc7       QLVTLFSAPNYCGEFDNAGA---MMSVDESLLCSFQILKPAEKKqKyvyg-gmgt--grp
1Bcin_PP1-A      QLVTLFSAPNYCGEFDNAGA---MMSVDESLLCSFQILKPAEKKqKyvtt-gglgassrp
1Nc_PP1          QLVTLFSAPNYCGEFDNAGA---MMSVDESLLCSFQILKPAEKKqKfgrr----------
1Mthe_PP1        QLVTLFSAPNYCGEFDNAGA---MMSVDESLLCSFQILKPAEKKqKfgrg-r--------
1Bbas_PP1        QLVTLFSAPNYCGEFDNAGA---MMSVDESLLCSFQILKPAEKKqKfgrr----------
1Anam_PP1        QLVTLFSAPNYCGEFDNAGA---MMSVDESLLCSFQILKPAEKKqnrfgr-r--------
1Npar_PP1        QLVTLFSAPNYCGEFDNAGA---MMSVDESLLCSFQILKPAEKKqKyvsm-gs----akp
1Bory_Glc7       QLVTLFSAPNYCGEFDNAGA---MMSVDESLLCSFQILKPAEKKqKyvpn-lgrpasg--
1Uree_PP1        QLVTLFSAPNYCGEFDNAGA---MMSVDESLLCSFQILKPAEKKqKyvyg-gmst--grp
1Tver_PP1        QLVTLFSAPNYCGEFDNAGA---MMSVDESLLCSFQILKPAEKKqKyvyg-gmap--nrp
1Anid_Glc7       QLVTLFSAPNYCGEFDNAGA---MMSVDESLLCSFQILKPAEKKqKyvyg-amss--grp
1Aory_Glc7       kLVTLFSAPNYCGEFDNAGA---MMSVDESLLCSFQILKPAEKKkanitr-gfksdrsnp
1Af_PP1          QLVTLFSAPNYCGEFDNAGA---MMSVDESLLCSFQILKPAEKKqKyvyg-gmss--gap
3Sp_PP1-1        QLVTLFSAPNYCGEFDNAGA---MMSVDESLLCSFQILKPAEKKqRyg-y-qgssqn-wh
3Sp_PP1-2        QLVTIFSAPNYCGEFDNvGA---MMSVNEdLLCSFQILKPAEKRqRvsqssikesks---
3Sj_PP1          QLVTLFSAPNYCGEFDNAGA---MMSVDESLLCSFQILKPAEKKqRygh-----------
3Sj_Sds21        hLVTIFSAPNYCGEFDNvGA---MMSVNDnLLCSFQILKPAEKRqRnsqvlahearr---
3Scot_PP1        QLVTLFSAPNYCGEFDNAGA---MMSVDESLLCSFQILKPAEKKqRyggy-qgmgqn-wh
3Scot_Sds21      lLVTIFSAPNYCGEFDNvGA---MMSVNEdLLCSFQILKPAEKRaRnhplagkepkp---
3Scry_PP1        QLVTLFSAPNYCGEFDNAGA---MMSVDESLLCSFQILKPAEKKqRyggy-qgmgqn-wh
3Scry_Sds21      lLVTIFSAPNYCGEFDNvGA---MMSVNEdLLCSFQILKPAEKRaRnhplsgkepks---
3Plac_PP1        QLVTLFSAPNYCGEFDNAGA---MMSVDESLLCSFQILKPAEKKqKyqyg-gmgs--grp
3Pjir_PP1        QLVTLFSAPNYCGEFDNAGA---MMSVDESLLCSFQILKPAEKKqKylyg-gmgs--grp
3Nirr_PP1        QLVTLFSAPNYCGEFDNAGA---MMSVDESLLCSFQILKPAEKKqKyvyg-gmgt--grp
4GALMADRAFT_Glc7 hLVTLFSAPNYCGEFDNAGA---MMSVDETLLCSFQILKPAEKKaKyp-y--ggmnmgrp
4PILCRDRAFT_Glc7 hLVTIFSAPNYCGEFDNAGA---MMSVDETLLCSFQILKPAEKKaKyp-y--ggmnmgrp
4Shir_Glc7       QLVTLFSAPNYCGEFDNAGA---MMSVDETLLCSFQILKPAEKKaKyp-y--ggmnmgrp
4Sver_PP1        QLVTLFSAPNYCGEFDNAGA---MMSVDETLLCSFQILKPAEKKaKya-y--namggk-n
4Pgig_PP1        hLVTLFSAPNYCGEFDNAGA---MMSVDETLLCSFQILKPAEKKpRlp-y--gsgntgrp
4Bbot_PP1        QLVTLFSAPNYCGEFDNAGA---MMSVDETLLCSFQILKPAEKK-Kyp-y--ggmnmgrp
4Cvis_PP1-A      QLVTLFSAPNYCGEFDNAGA---MMSVDETLLCSFQILKPAEKKsKypgyggmtmggnrp
4Cvis_PP1-B      QLVTIFSAPNYCGEFDNAGA---MMSVDESLLCSFQILKPAEKKpsklttmkkkgkgtrf
4Cgat_PP1        QLVTLFSAPNYCGEFDNAGA---MMSVDDTLLCSFQILKPAEKKpKyggyggs----arp
4Cneo_PP1        QLVTLFSAPNYCGEFDNAGA---MMSVDDTLLCSFQILKPAEKKpKyggyggs----arp
4Tmes_PP1        QLVTLFSAPNYCGEFDNAGA---MMSVDDTLLCSFQILKPAEKKtnrnpygap----grr
4Umay_Glc7       QLVTLFSAPNYCGEFDNAGA---MMSVDETLLCSFQILKPAEKKqKya-y--gginmgrp
4Phub_PP1        QLVTLFSAPNYCGEFDNAGA---MMSVDETLLCSFQILKPAEKKqKya-y--gginmgrp
5Wich_Glc7       QLVTLFSAPNYCGEFDNAGA---MMSVDETLLCSFQILKPAEKKqKfpas----ygigrp
5Rgra_PP1        QLVTLFSAPNYCGEFDNAGA---MMSVDETLLCSFQILKPAEKKktfa-y--ggagqgrp
5Pgra_PP1        QLVTLFSAPNYCGEFDNAGA---MMSVDETLLCSFQILKPAEKKqKfm-y--ggmntgrp
6Rdel_PP1        QLVTIFSAPNYCGEFDNAGA---MMSVDDSLMCSFQILKPAEKRsKytgytgsknasrgk
6Ccor_PP1        QLVTLFSAPNYCGEFDNAGA---MMSVDDTLMCSFQILKPAEKKqKfayg---gintgkp
6Crev_PP1        kLVTLFSAPNYCGEFDNAGA---MMSVDDTLMCSFQILKPAEKKpRggrrr---------
6Spun_PP1        QLVTLFSAPNYCGEFDNAGA---MMSVDETLMCSFQILKPAEKKtKyayg---gmnsgrp
6Gpro_PP1-A      QLVTLFSAPNYCGEFDNAGA---MMSVDETLMCSFQILKPAEKKqKyayg---gissgrp
6Gpro_PP1-B      QLVTIFSAPNYCGEFDNAGA---IMSVDESLMCSFQILKPAEKKqKyvyggpggsssgrg
6AMAG_PP1-A      QLVTLFSAPNYCGEFDNAGA---MMSVDETLMCSFQILKPAEKKqKhnygagnrskfa--
6Rallo_PP1       QLVTLFSAPNYCGEFDNAGA---MMSVDETLMCSFQILKPAEKKqKyayg---gmntgrp
```

```
                    ....:...10....:...20....:...30....:...40....:...50....:...60
2YALI_Ppz1          sLVTVFSAPNYCGEFDNWGA---VMSVseELLCSFELLkPLDsaaLKqeMkKg-----kh
2Mgui_Ppz1          tLVTVFSAPNYCGEFDNWGA---VMSVsdELLCSFELLdPLDsvaLKqvMkKg-----rq
2DEHA_Ppz1          tLVTVFSAPNYCGEFDNWGA---VMSVsdELLCSFELLdPLDsiaLKqvMkKg-----ke
2Mfar_Ppz           tLVTVFSAPNYCGEFDNWGA---VMSVseELLCSFELLdPLDssaLKqvMkKg-----ri
2Ct_Ppz1            tLVTVFSAPNYCGEFDNWGA---VMgVseDLLCSFELLdPLDsvaLKqvMkKe-----kq
2Cgla_Ppz1          tLVTVFSAPNYCGEFNNWGA---VMSVsqELLCSFELLdPLDsnsLKqvMkKg-----kq
2Cgla_Ppz2          sLVTVFSAPNYCGEFDNWGA---VMSVsegLMCSFELLdPLDsaaLKqvMkKg-----rq
2Ca_Ppz1            tLVTVFSAPNYCGEFDNWGA---VMgVseDLLCSFELLdPLDsaaLKqvMkKe-----kq
2Cdub_Ppz           tLVTVFSAPNYCGEFDNWGA---VMgVseDLLCSFELLdPLDstaLKqvMkKe-----kq
2Klac_PpzA          sLVTVFSAPNYCGEFDNWGA---VMSVsegLLCSFELLePLDstaLKqvMkKg-----rq
2Klac_PpzB          sLVTVFSAPNYCGEFDNWGA---VMSVsegLLCSFELLePLDstaLKqvMkKg-----rq
2Lther_Ppz1         sLVTVFSAPNYCGEFDNWGA---VMSVsegLLCSFELLdPLDsaaLKqvMkKg-----rq
2Ag_Ppz             sLVTVFSAPNYCGEFDNWGA---VMSVsegLLCSFELLdPLDsaaLKqvMkKg-----rq
2Ecym_Ppz           sLVTVFSAPNYCGEFDNWGA---VMSVsegLLCSFELLdPLDsaaLKqvMkKg-----rq
2Tbla_PpzA          sLVTVFSAPNYCGEFDNWGA---VMSVsegLLCSFELLdPLDsaaLKqvMkKg-----rq
2Tdel_PpzA          sLVTVFSAPNYCGEFDNWGA---VMSVsegLLCSFELLdPLDgaaLKqvMkKg-----rq
2Sc_PPZ1            sLVTVFSAPNYCGEFDNWGA---VMSVsegLLCSFELLdPLDsaaLKqvMkKg-----rq
2Sc_PPZ2            sLVTVFSAPNYCGEFDNWGA---VMTVsegLLCSFELLdPLDstaLKqvMkKg-----rq
1Tmel_Ppz1          tLVTVFSAPNYCGEFDNWGA---VMSVsgELLCSFELLkPLDsaaLKqqMkKg-----rg
1Bcin_Ppz           vLVTVFSAPNYCGEFDNYGA---VMSVstELLCSFELLkPLDssaLKsqMkKs-----rn
1Nc_PP-Z            iLVTiFSAPNYCGEFDNWGA---VMaVstELLCSFELLkPLDsaaLKthIkKg-----rh
1Mthe_Ppz           vLVTVFSAPNYCGEFDNWGA---VMSVsaELLCSFELLkPLDsnaLKshIkKg-----rn
1Bbas_Ppz           vLVTVFSAPNYCGEFDNWGA---VMSVnaELLCSFELLkPLDssaLKshIkKg-----rn
1Anam_Ppz           vLVTVFSAPNYCGEFDNWGA---VMSVsgELLCSFELLkPLDssaLKrhIkKg-----rn
1Npar_Ppz1          iLVTVFSAPNYCGEFDNWGA---VMSVssELLCSFELLkPLDssaLKshIkKg-----rn
1Bory_Ppz1          vLVTVFSAPNYCGEFDNWGA---VMSVsgELLCSFELLkPLDssaLKshIkKs-----rn
1Uree_Ppz           iLVTVFSAPNYCGEFDNWGA---IMSVsaELLCSFELLkPLDssaLKshIkKg-----rn
1Tver_Ppz           iLVTVFSAPNYCGaFDNWGA---IMSVsdELLCSFELLkPLDssaLKnhIkKg-----rn
1Anid_Ppz1          iLVTVFSAPNYCGEFDNWGA---VMSVsgELLCSFELLkPLDsaaLKshIkKg-----rk
1Aory_Ppz1          iLVTVFSAPNYCGEFDNWGA---IMSVsdELLCSFELLkPLDstaLKnhIkKg-----rn
1Af_PPZ             iLVTVFSAPNYCGEFDNWGA---IMSVsgELLCSFELLkPLDstaLKnhIkKg-----rk
3Sp_PPZ             tLcTVFSAPNYCGEFDNWGA---VMSVnsELLCSFELIkPLDqaaIRreLkKs-----kr
3Sj_Pzh1            tLcTVFSAPNYCGEFDNWGA---VMSVnpELLCSFELLkPLDqaaIRreLkKs-----kl
3Soct_Ppz           tLcTVFSAPNYCGEFDNWGA---VMSVnsELLCSFELIkPLDqaaIRreLkKs-----kh
3Scry_Ppz           tLcTVFSAPNYCGEFDNWGA---VMSVnsELLCSFELIkPLDqaaIRreLkKs-----kr
3Plac_Ppz           tLVTVFSAPNYCGEFDNFGmytvVMcVseELLCSFELLkPLDasaLKqhMkKg-----ds
3Pjir_Ppz-A         tLVTVFSAPNYCGEFDNFGmytvVMcVseELLCSFELLkPLDasaLKqhMkKg-----ds
3Nirr_PP-Z          tLVTVFSAPNYCGEFDNFGA---VMSVseELLCSFELLkPLDqiaLKqeMkKr-----rr
4GALMADRAFT_Ppz1    tLVTVFSAPNYCGEFDNYGA---cMSVseDLLCaFELLkPLDgaaLRkeMtKa-----kr
4PILCRDRAFT_Ppz1    tLVTVFSAPNYCGEFDNYGA---cMSVseDLLCaFELLkPLDgpaLRkeMtKa-----kr
4Shir_Ppz           tLVTVFSAPNYCGEFDNYGA---cMSVseDLLCaFELLkPLDgpaLRkeMtKa-----kr
4Sver_PPZ           tLVTVFSAPNYCGEFDNYGA---cMSVseDLLCaFELLkPLDpavIKkeLsKa-----kr
4Pgig_PPZ           tLVTVFSAPNYCGEFDNYGA---cMSVseELLCaFELLkPLDgpaLRkeMtKa-----kr
4Bbot_PPZ           tLVTVFSAPNYCGEFDNYGA---cMSVseDLLCaFELLkPLDgpaLRkeMtKa-----rr
4Cvis_PPZ           tLVTVFSAPNYCGEFDNYGA---cMSVseDLLCaFELLkPLDgpaLRkeMtKa-----kr
4Cgat_PPZ           tLVTVFSAPNYCGEFDNFGA---VMSVseDLLCSFELLkPLDgaaLKkeMtKs-----kr
4Cneo_PPZ           tLVTVFSAPNYCGEFDNFGA---VMSVseDLLCSFELLkPLDgaaLKkeMtKs-----kr
4Tmes_PPZ           tLVTVFSAPNYCGEFDNFGA---VMSVseDLLCSFELLkPLDgaaLKkeMtKs-----kr
4Umay_PPZ           tLVTiFSAPNYCGEFDNFGA---VMSVseDLLCaFELLkPLDgaaLKkeMaKn-----kr
4Phub_PPZ           tLVTiFSAPNYCGEFDNFGA---VMSVseDLLCaFELLkPLDgaaLKkeMaKn-----kr
5Wich_PP-Z          tLVTiFSAPNYCGEFDNFGA---IMSVsqDLLCaFELLkPLDsqaLKkeMhKs-----rk
5Rgra_PPZ1          aLVTiFSAPNYCGEFDNFGA---VMSVslDLLCSFELLkPLDtaqLKreLaKt-----rr
5Pgra_PPZ           tLVTiFSAPNYCGgtssmon---okaryo-tICfuNgI----------------------
6Rdel_PPZ-A         sLVTVFSAPNYCGEFDNFGA---IMSVneELLCSFELLtPaDhplaKerLkqnkansnn-
6Rdel_PPZ-B         tLVTVFSAPNYCGEFDNFGA---IMSineELLCSFELLtPLEhpstaklqeaqrn-rgrr
Rdel_PPZ-C          tLVTVFSAPNYCGEFDNFGA---IMSVneELLCSFELLtPaDhplaKerLkqnkinnkr-
6Rdel_PPZ-D         tLVTiFSAPNYCGEFDNFAA---IMSVneELLCSFELLtPMDhpltvksr----------
6Rdel_PPZ-E         sLVTiFSAPNYCGEFDNYGA---VMTiskDLLCSFELLpPaEtvisHeknlKksssslql
6Rdel_PPZ-F         sLVTVFSAPNYCGEFDNYGA---VMTiskDLLCSFEMLpPIqavtpqqqenK--------
6Rdel_PPZ-G         sLVTVFSAPNYCGEFDNFGA---IMSVneELLCSFELLtPaDhplaKerLkqnkansnn-
6Ccor_PPZ           tLVTVFSAPNYCGEFDNYGA---VMSVneELLCSFELLkPqDlsapqrrkrns-----pp
6Crev_PPZ           qLVTVFSAPNYCGEFDNsGA---VMnVneELLCSFEILkPLvenlhtcmsrveasfwsrr
6Spun_PPZ           tLVTVFSAPNYCGEFDNkGA---VMcVneDLLCSFEILqPqthpkhslkgrRr-------
6Gpro_PPZ           iLVTVFSAPNYCGEFDNnGA---VMSVneELLCSFEILtPvtvtkkHnmrrRr-------
6AMAG_PPZ-A         sLVTiFSAPNYCGEFNNlGA---VMcVneDLMCTFEILkPegsvrIvngrrRis------
6AMAG_PPZ-B         sLVTiFSAPNYCGEFNNlGA---VMcVneDLMCTFEILkPegsvrIvngrrRis------
6Rallo_Ppz1         tLVTVFSAPNYCGEFENcGA---ILqVsqnLLCSFQIIknssplykissedink------
```

```
Consensus PP1     qLVTlFSAPNYCGEFDNaGA   mMSVdesLlCSFQILKPAeKk k
consensus PPZ      LvTvFSAPNYCGefdnwga   vmsv  ellCsfell pld   lk  m k
```

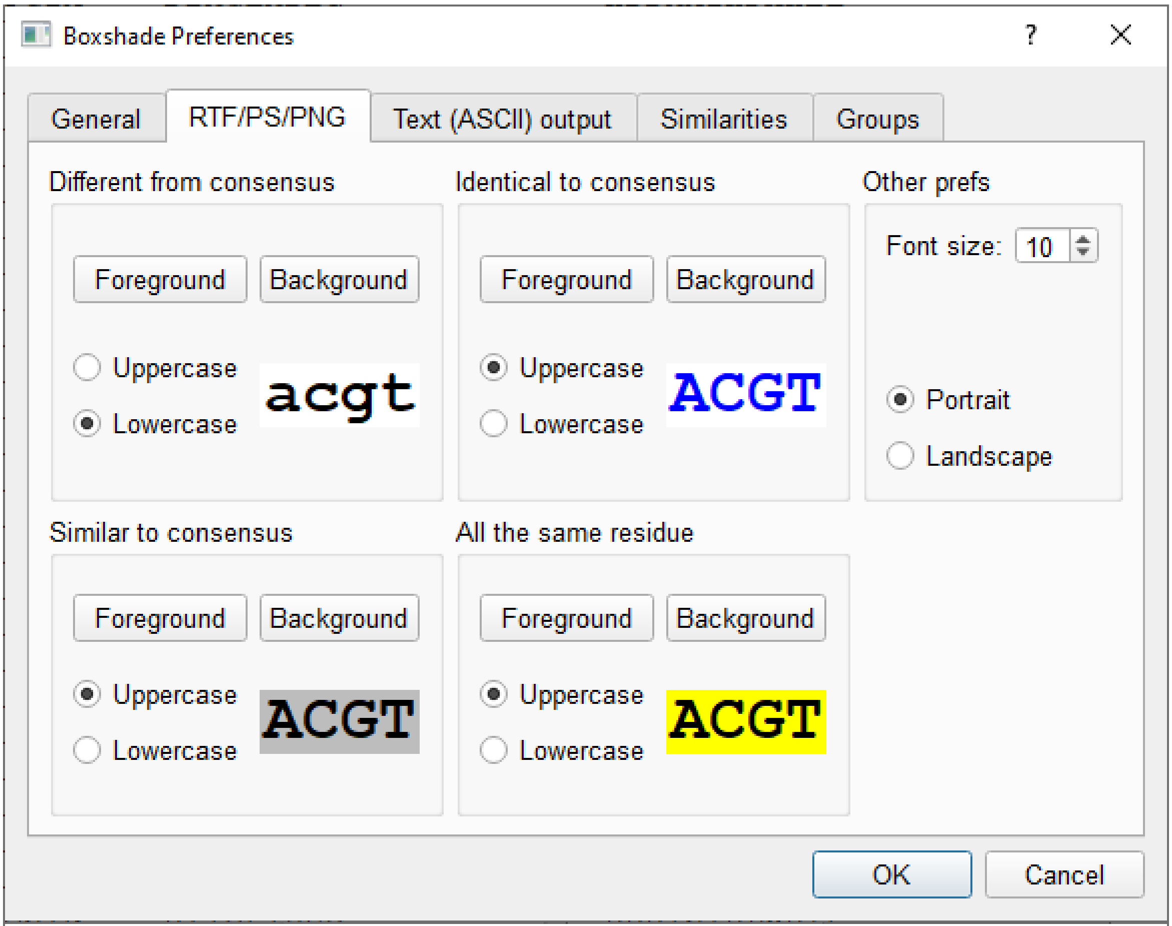
Boxshade Preferences
General
RTF/PS/PNG
Text (ASCII) output
Similarities
Groups
Different from consensus
Foreground
Background
Uppercase
Lowercase
acgt
Identical to consensus
Foreground
Background
Uppercase
Lowercase
ACGT
Other prefs
Font size: 10
Portrait
Landscape
Similar to consensus
Foreground
Background
Uppercase
Lowercase
ACGT
All the same residue
Foreground
Background
Uppercase
Lowercase
ACGT
OK
Cancel

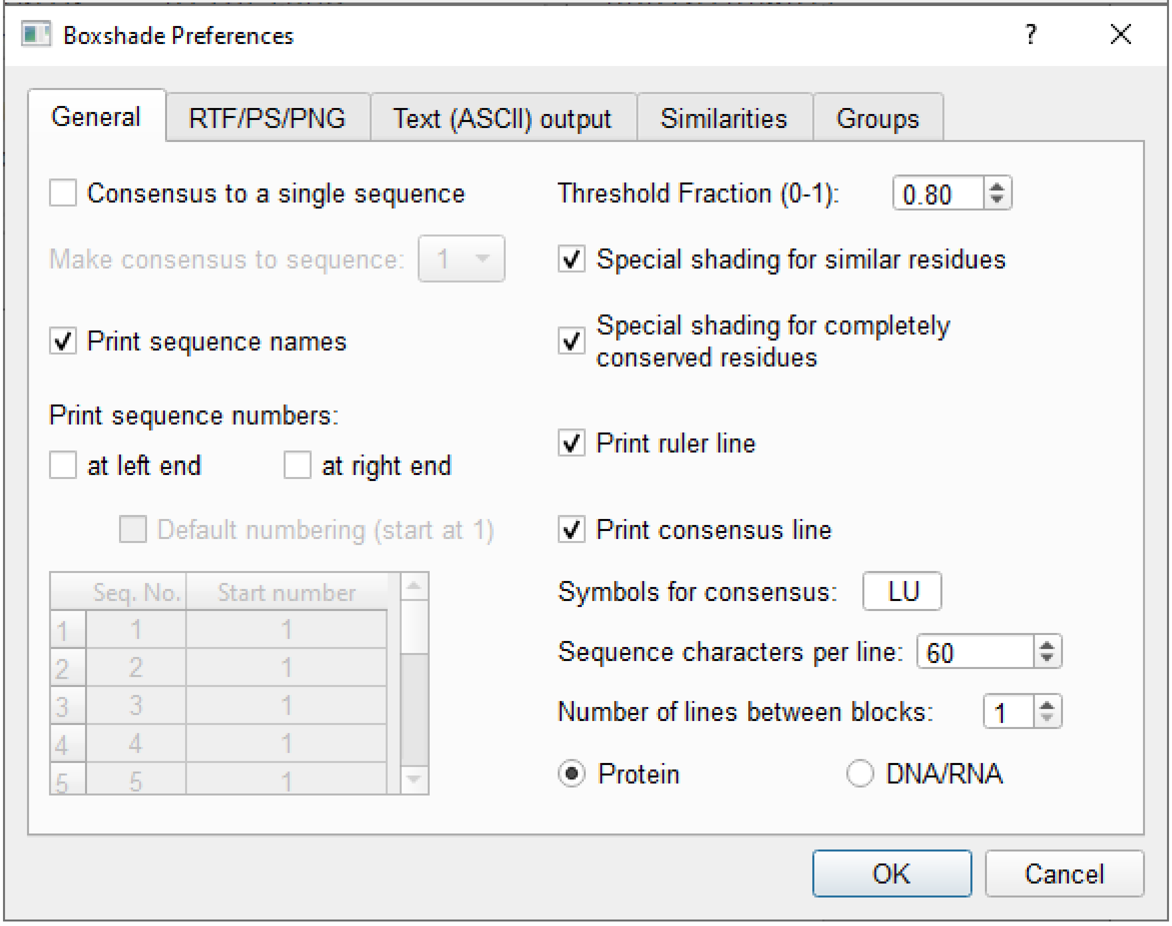
Boxshade Preferences
General
RTF/PS/PNG
Text (ASCII) output
Similarities
Groups
Consensus to a single sequence
Make consensus to sequence: 1
Print sequence names
Print sequence numbers:
at left end
at right end
Default numbering (start at 1)
Seq. No.
Start number
1 1 1
2 2 1
3 3 1
4 4 1
5 5 1
Threshold Fraction (0-1): 0.80
Special shading for similar residues
Special shading for completely conserved residues
Print ruler line
Print consensus line
Symbols for consensus: LU
Sequence characters per line: 60
Number of lines between blocks: 1
Protein
DNA/RNA
OK
Cancel
