# Supplementary material for: Comparative Analysis of Type 1 and Type Z Protein Phosphatases Reveals D615 as a Key Residue for Ppz1 Regulation
Source: Int J Mol Sci. 2022 Jan 25;23(3):1327. doi: 10.3390/ijms23031327 (PMC8836105; doi:10.3390/ijms23031327)

Figure S2. Conservation and divergence of structural features required for modulation by regulatory subunits

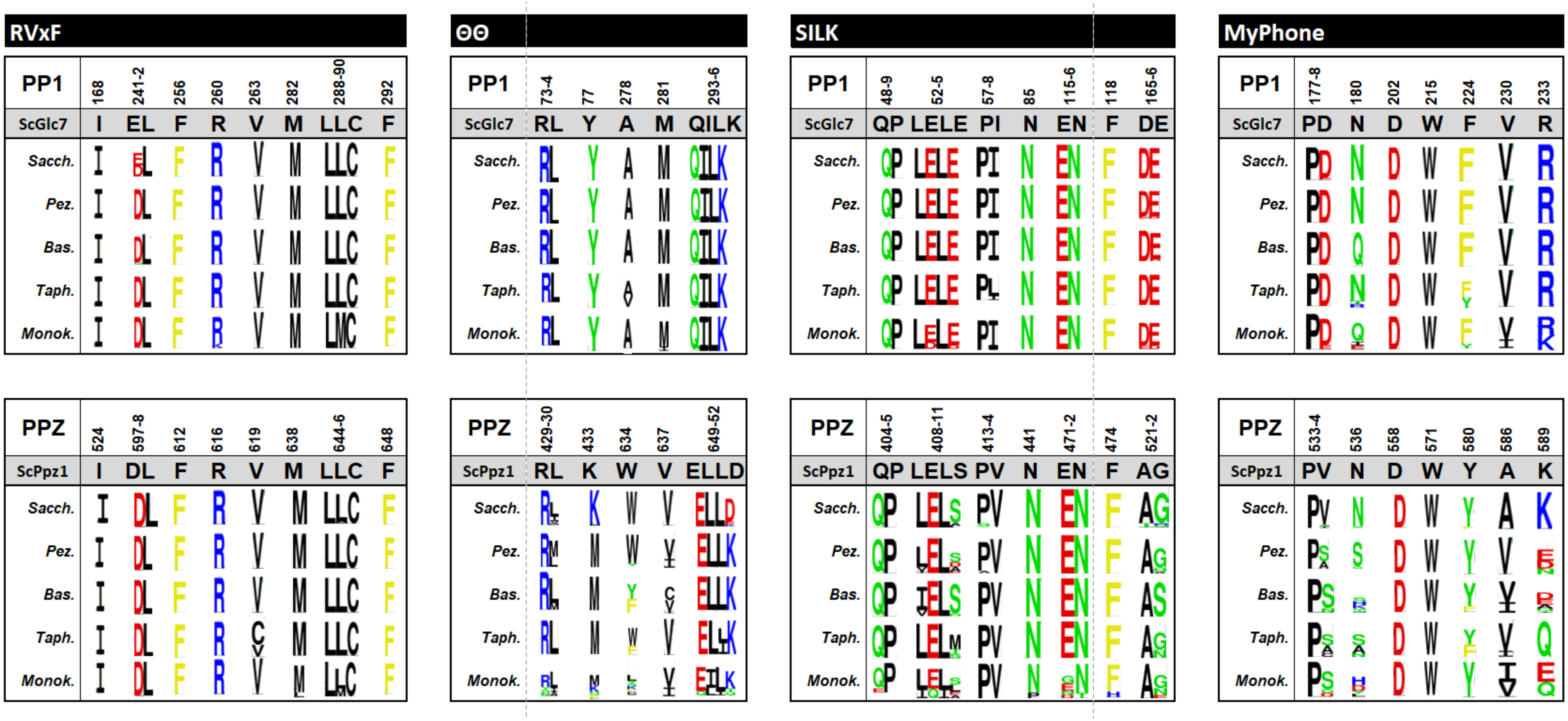

Figure S2. (cont.)

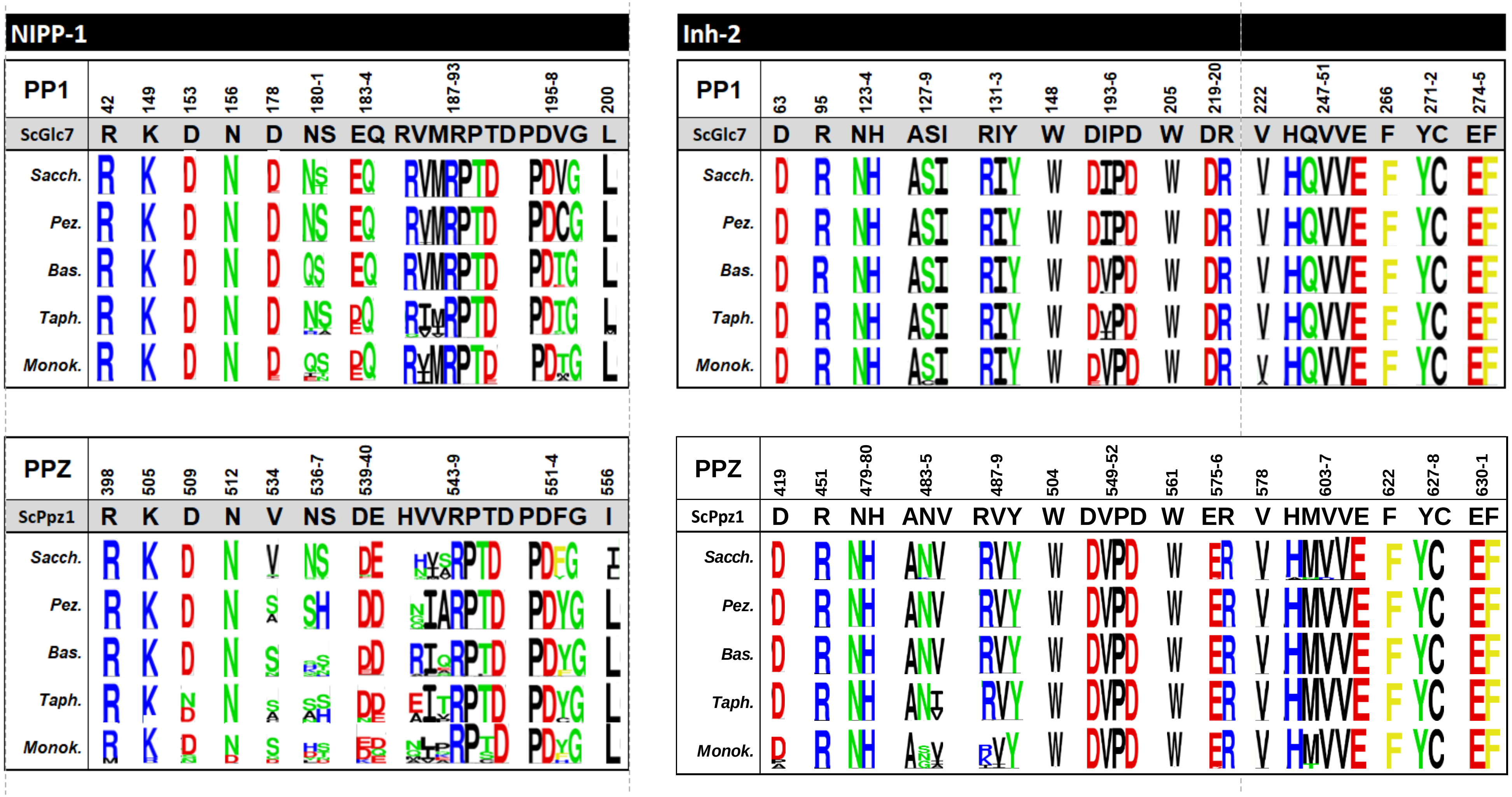

Figure S2. (cont.)

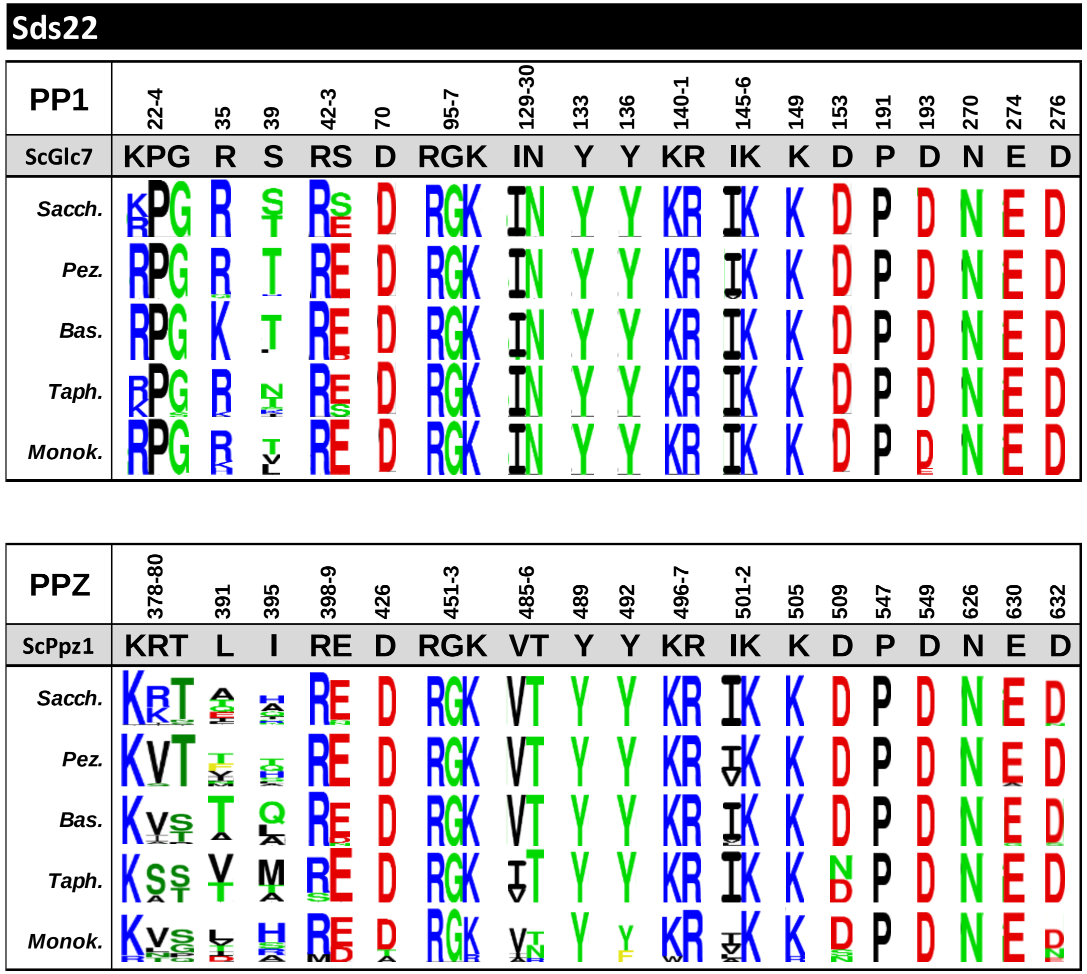

Supplement: Supplementary file 1 [file ijms-23-01327-s001.zip › Figure S2.pdf]
