# Supplementary material for: Comparative Analysis of Type 1 and Type Z Protein Phosphatases Reveals D615 as a Key Residue for Ppz1 Regulation
Source: Int J Mol Sci. 2022 Jan 25;23(3):1327. doi: 10.3390/ijms23031327 (PMC8836105; doi:10.3390/ijms23031327)
Supplement: Supplementary file 1 [file ijms-23-01327-s001.zip › Figure S3 (xlinks-figure_combined) revised.pdf]

Figure S3.

Representative cross-linking scans.

Ppz1 and Hal3 were crosslinked with BS3 and analyzed by mass spectrometry as described in Materials and Methods.

Identified b-ions are shown as green, y-ions are shown as blue, purple peaks are fragment loss (denoted '*' for NH_3 and 'o' for H_2O), and light blue peaks represent internal fragments. Red peaks represent non-identified peaks. In the sequence lines, red ‘K’ are the cross-linked lysine residues and ‘:’ shows preferred cleavage positions.

378 x 113

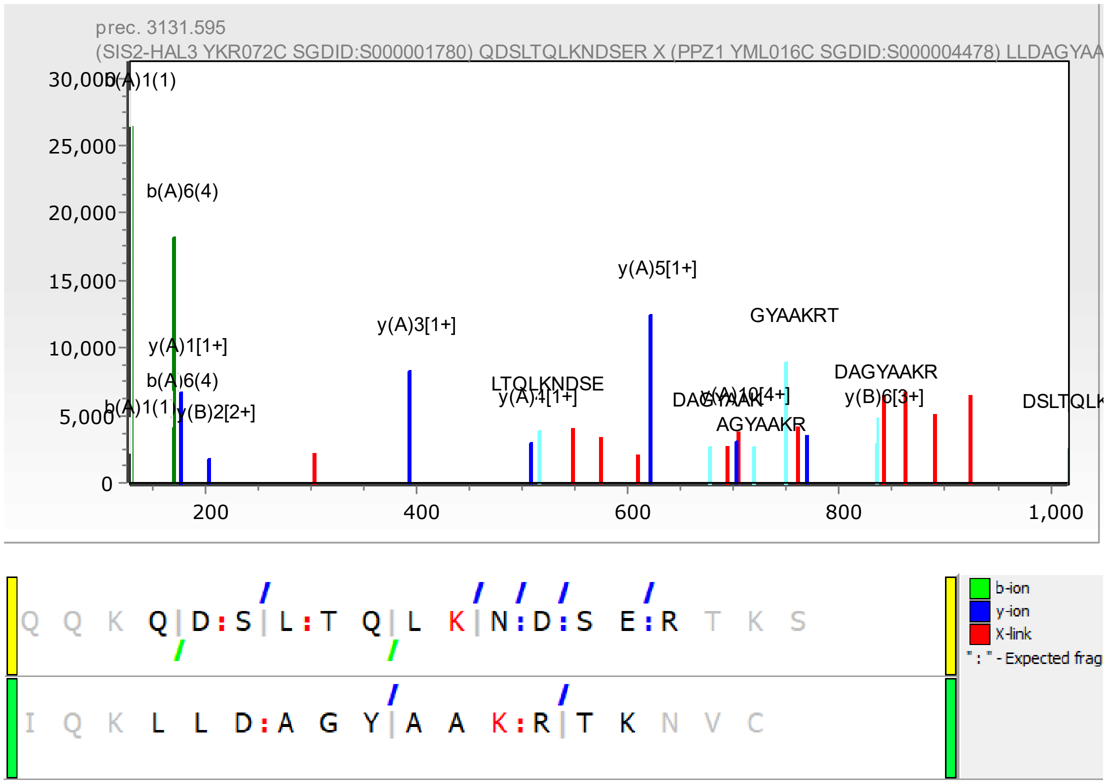

378 x 197

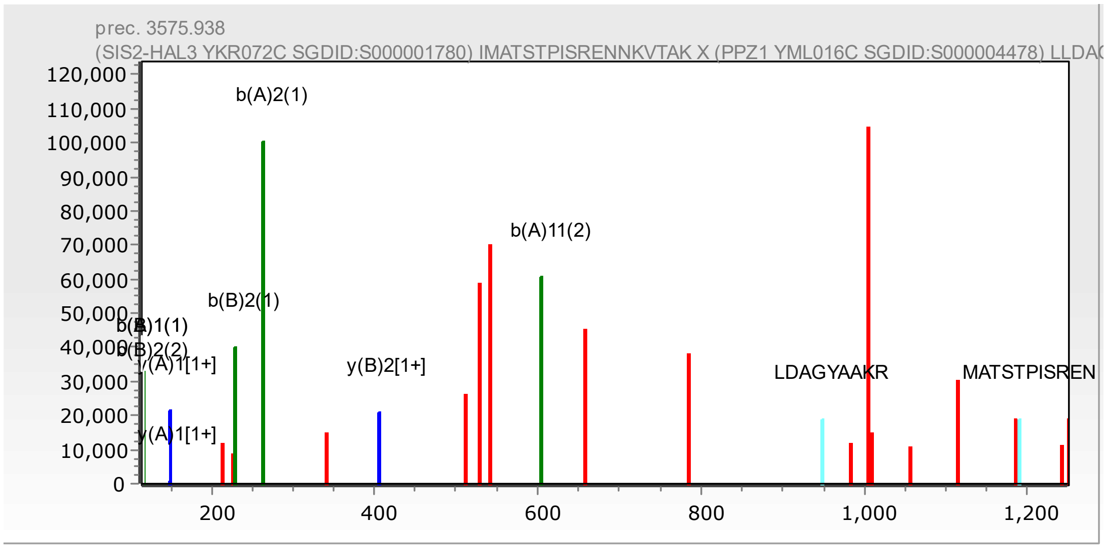
prec. 3575.938
(SIS2-HAL3 YKR072C SGDID:S000001780) IMATSTPISRENNKVTAK X (PPZ1 YML016C SGDID:S000004478) LLDA
b(A)2(1)
b(A)11(2)
b(B)2(1)
y(B)2[1+]
LDAGYAAKR
MATSTPISREN

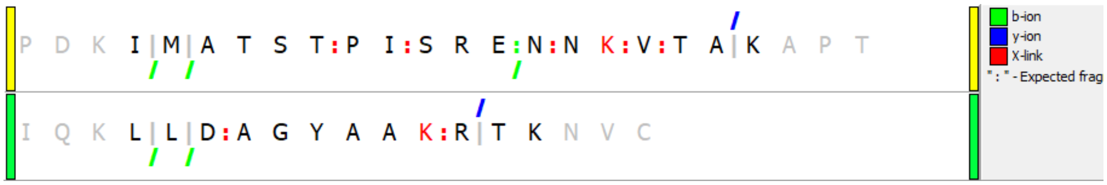
P D K I M A T S T P I S R E N N K V T A K A P T
I Q K L L D A G Y A A K R T K N V C
b-ion
y-ion
X-link
": " - Expected frag

381x 210

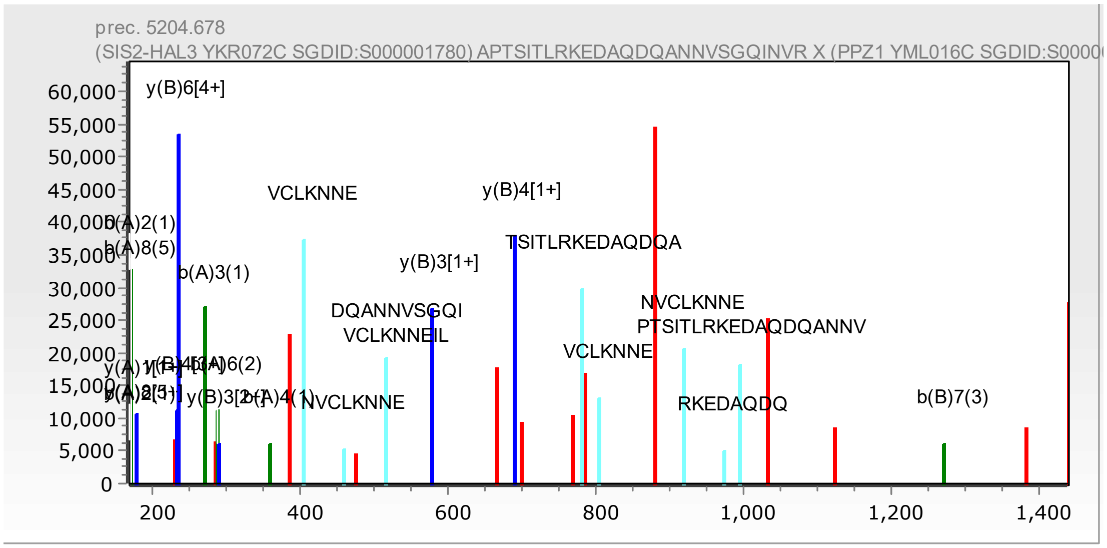

prec. 5204.678
(SIS2-HAL3 YKR072C SGDID:S000001780) APTSITLRKEDAQDQANNVSGQINVR X (PPZ1 YML016C SGDID:S0000
y(B)6[4+]
b(A)2(1)
b(A)8(5)
b(A)3(1)
VCLKNNE
y(B)4[1+]
TSITLRKEDAQDQA
y(B)3[1+]
DQANNVSGQI
VCLKNNEIL
NVCLKNNE
PTSITLRKEDAQDQANNV
VCLKNNE
NVCLKNNE
RKEDAQDQ
b(B)7(3)
60,000
55,000
50,000
45,000
40,000
35,000
30,000
25,000
20,000
15,000
10,000
5,000
0
200
400
600
800
1,000
1,200
1,400

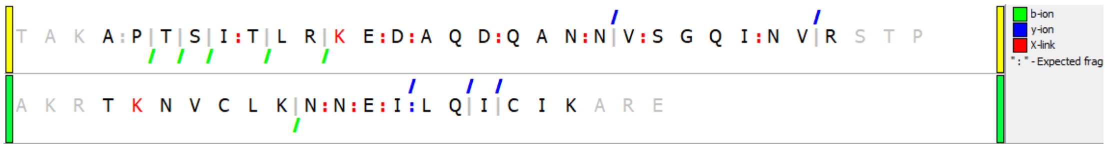

T A K A:P|T|S|I:T|L R|K E:D:A Q D:Q A N:N|V:S G Q I:N V|R S T P
A K R T K N V C L K|N:N:E:I:L Q|I|C I K A R E
b-ion
y-ion
X-link
":" - Expected frag

396x236

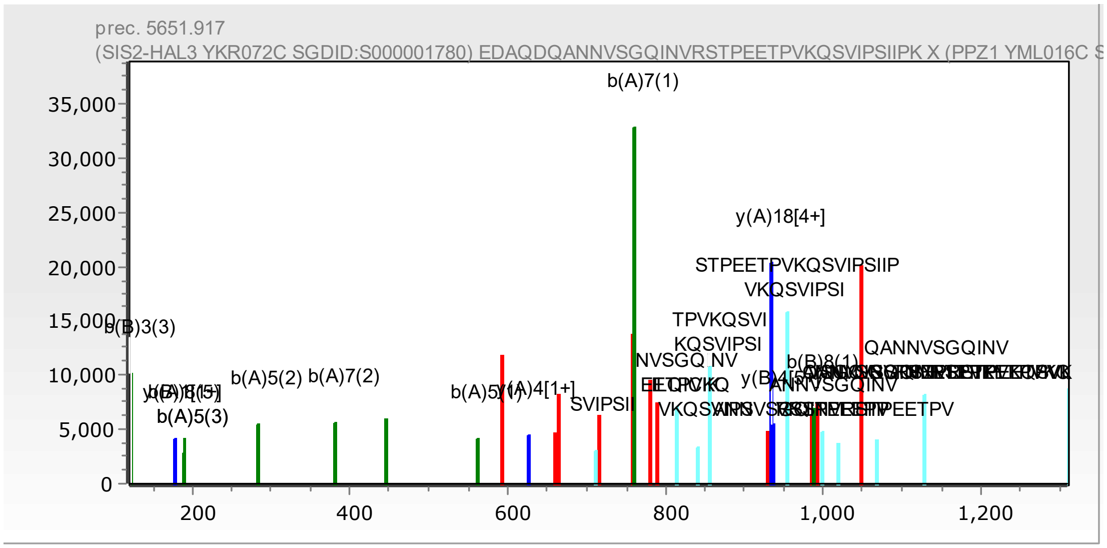
prec. 5651.917
(SIS2-HAL3 YKR072C SGDID:S000001780) EDAQDQANNVSGQINVRSTPEETPVKQSVIPSIIPK X (PPZ1 YML016C S
b(A)7(1)
y(A)18[4+]
STPEETPVKQSVIPSIIP
VKQSVIPSI
TPVKQSVI
KQSVIPSI
NVSGQ NV
QANNVSGQINV
ANNVSGQINV
SVIPSII
VKQS
PEETPV
b(B)3(3)
b(A)5(3)
b(A)5(2)
b(A)7(2)
b(B)8(1)
35,000
30,000
25,000
20,000
15,000
10,000
5,000
0
200
400
600
800
1,000
1,200

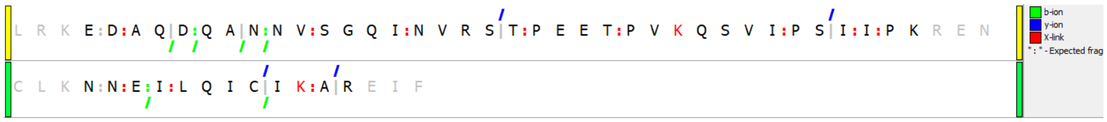
L R K E:D:A Q|D:Q A|N:N V:S G Q I:N V R S|T:P E E T:P V K Q S V I:P S|I:I:P K R E N
C L K N:N:E:I:L Q I C|I K:A|R E I F
b-ion
y-ion
X-link

664 x 113

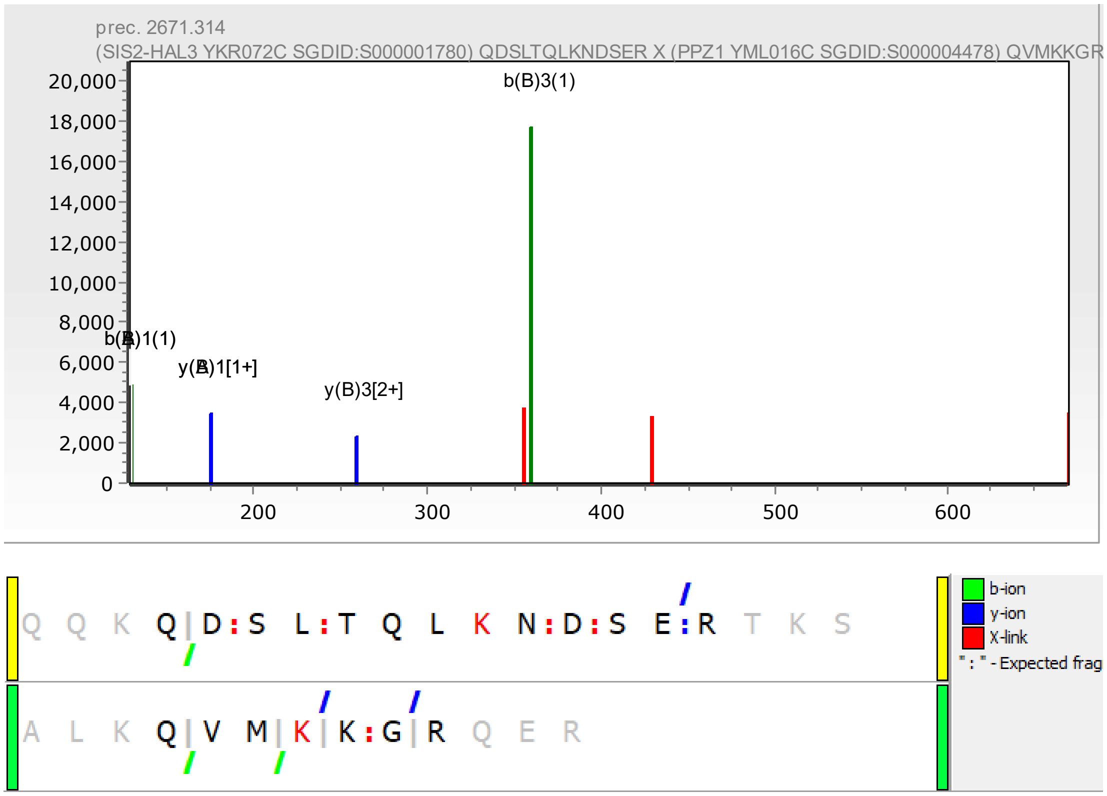
prec. 2671.314
(SIS2-HAL3 YKR072C SGDID:S000001780) QDSLTQLKNDSER X (PPZ1 YML016C SGDID:S000004478) QVMKKGR
b(B)3(1)
y(B)3[2+]
b-ion
y-ion
X-link
" : " - Expected frag

664x210

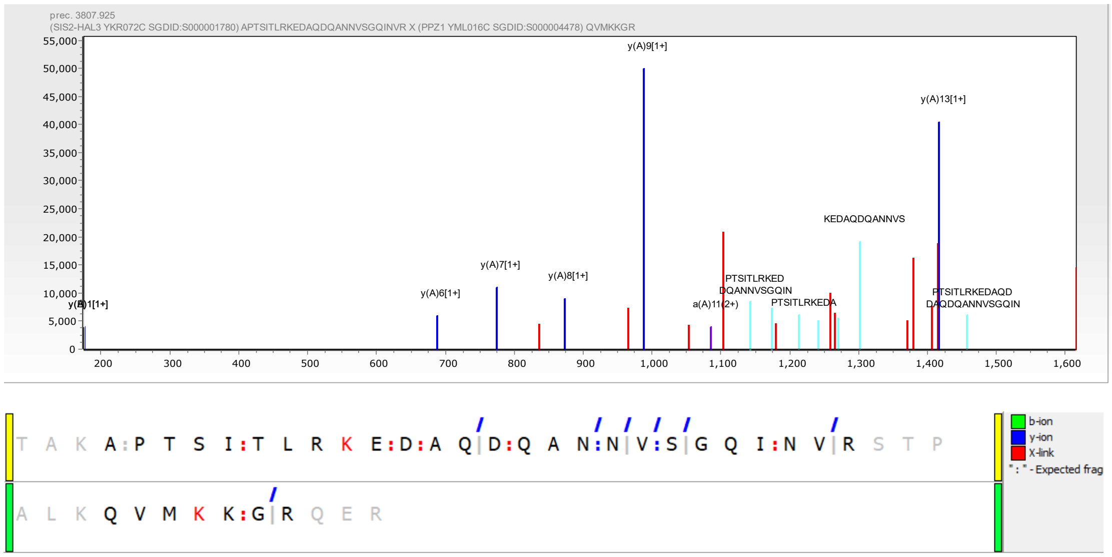
prec. 3807.925
(SIS2-HAL3 YKR072C SGDID:S000001780) APTSITLRKEDAQDQANNVSGQINVR X (PPZ1 YML016C SGDID:S000004478) QVMKKGR
y(A)9[1+]
y(A)13[1+]
KEDAQDQANNVS
y(A)7[1+]
y(A)8[1+]
y(A)6[1+]
PTSITLRKED
DQANNVSGQIN
PTSITLRKEDA
a(A)11(2+)
PTSITLRKEDAQD
DAQDQANNVSGQIN
b-ion
y-ion
X-link
" : " - Expected frag

664x113

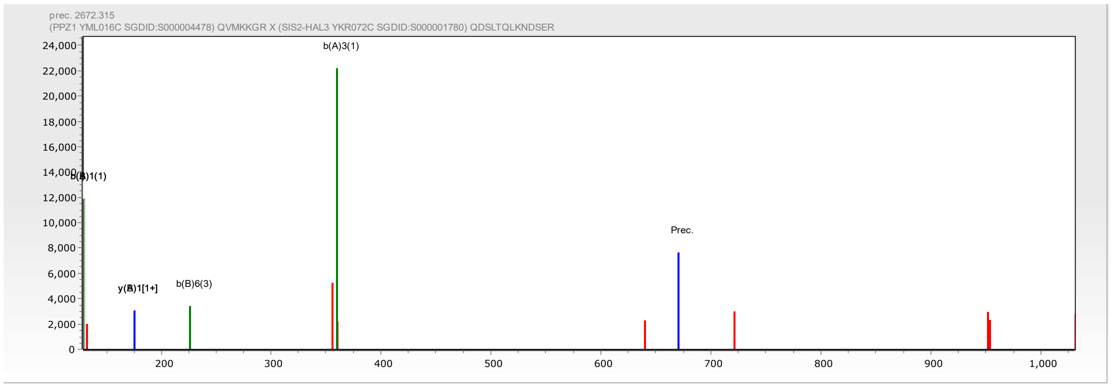
prec. 2672.315
(PPZ1 YML016C SGDID:S000004478) QVMKKGR X (SIS2-HAL3 YKR072C SGDID:S000001780) QDSLTQLKNDSER
b(A)3(1)
Prec.
b(B)6(3)

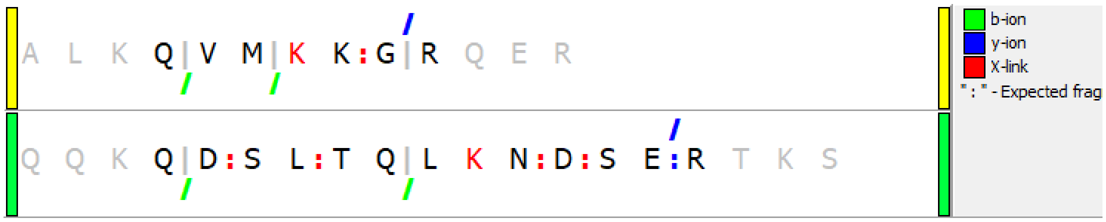
A L K Q V M K K G R Q E R
Q Q K Q D S L T Q L K N D S E R T K S
b-ion
y-ion
X-link
" : " - Expected frag

359 x 41

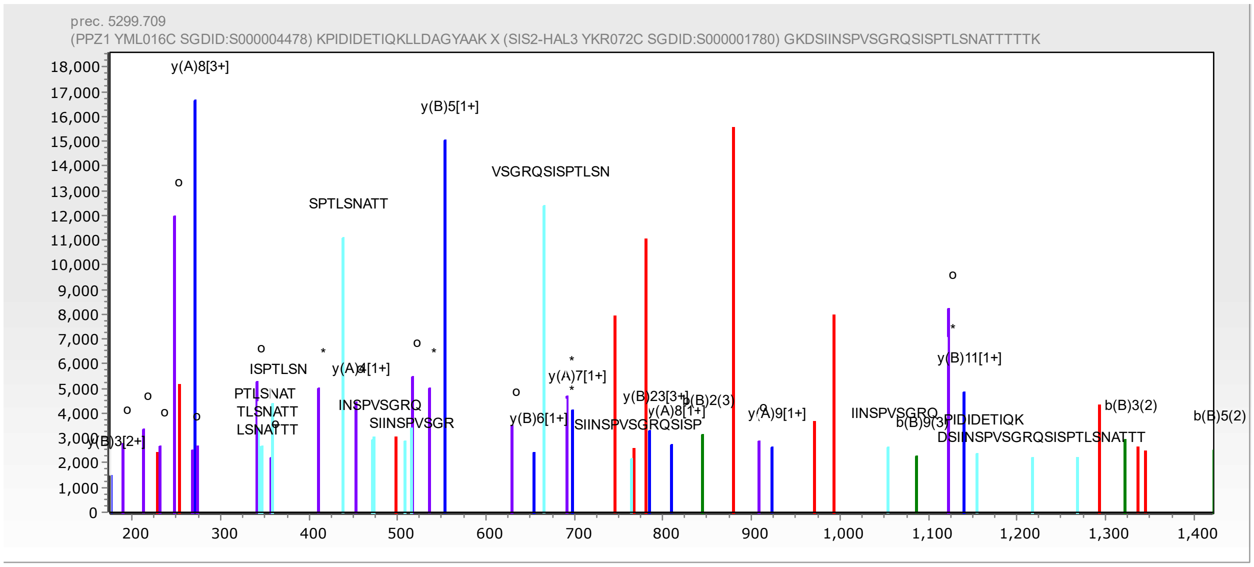
prec. 5299.709
(PPZ1 YML016C SGDID:S000004478) KPIDIDETIQKLLDAGYAAK X (SIS2-HAL3 YKR072C SGDID:S000001780) GKDSIINSPVSGRQSISPTLSNATTTTTK
y(A)8[3+]
y(B)5[1+]
VSGRQSISPTLSN
SPTLSNATT
ISPTLSN
PTLSNAT
TLSNATT
LSNATTT
y(A)4[1+]
INSPVSGRQ
SIINSPVSGR
y(B)3[2+]
y(B)6[1+]
y(A)7[1+]
SIINSPVSGRQSISP
y(B)23[3+]
y(A)8[1+]
b(B)2(3)
y(A)9[1+]
IINSPVSGRQ
b(B)9(3)
PIDIDETIQK
DSIINSPVSGRQSISPTLSNATTT
y(B)11[1+]
b(B)3(2)
b(B)5(2)

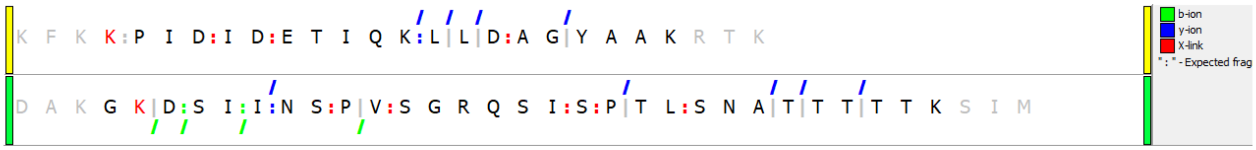
K F K K : P I D : I D : E T I Q K : L L L D : A G Y A A K R T K
D A K G K D : S I : I : N S : P V : S G R Q S I : S : P T L : S N A T T T T T K S I M
b-ion
y-ion
X-link
" : " - Expected frag

378 x 315 (316)

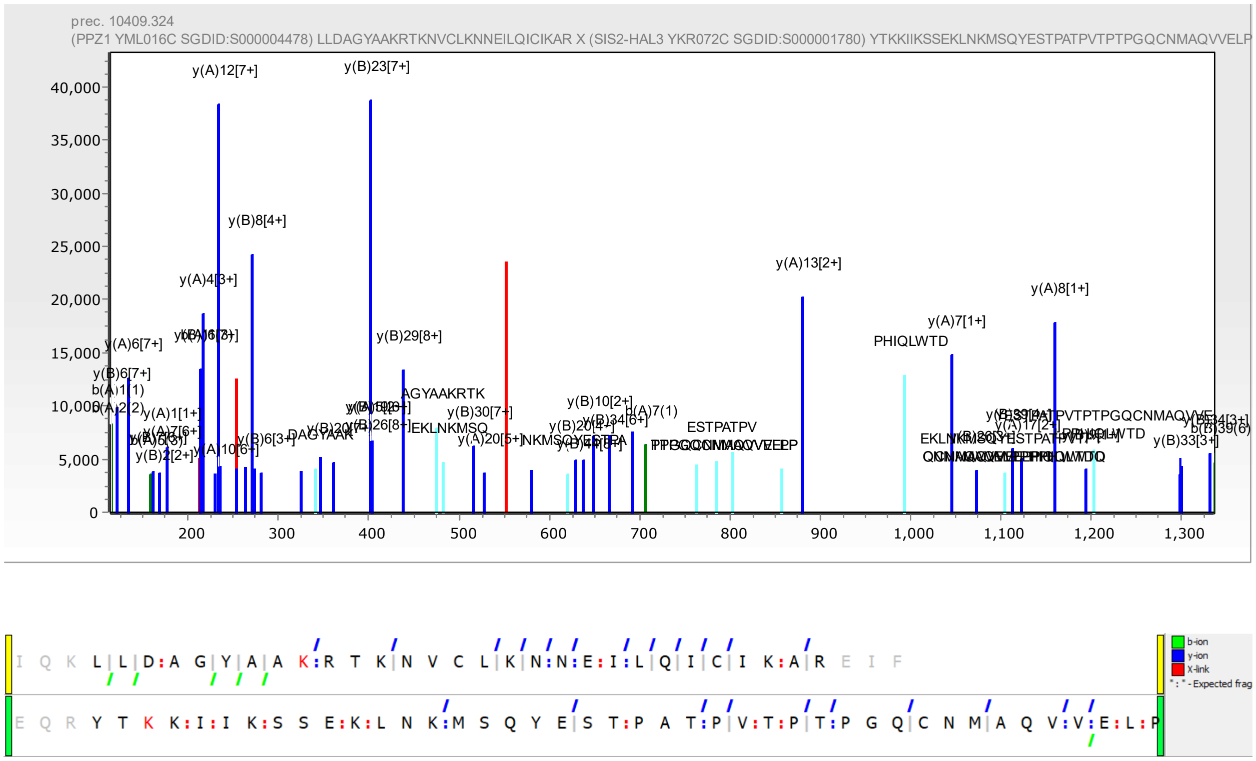
prec. 10409.324
(PPZ1 YML016C SGDID:S000004478) LLDAGYAAKRTKNVCLKNNEILQICIKAR X (SIS2-HAL3 YKR072C SGDID:S000001780) YTKKIIKSSEKLNKMSQYESTPATPVTPTPGQCNMAQVVELP
y(A)12[7+]
y(B)23[7+]
y(B)8[4+]
y(A)4[3+]
y(B)29[8+]
y(A)13[2+]
y(A)8[1+]
y(A)7[1+]
PHIQLWTD
AGYAAKRTK
y(B)30[7+]
y(B)10[2+]
b(A)7(1)
ESTPATPV
y(A)6[7+]
y(B)6[7+]
y(A)1[1+]
y(B)2[2+]
y(B)6[3+]
DAGYAAK
EKLNKMSQ
y(A)20[5+]
y(B)33[3+]
b-ion
y-ion
X-link
":" - Expected frag

381 x 483

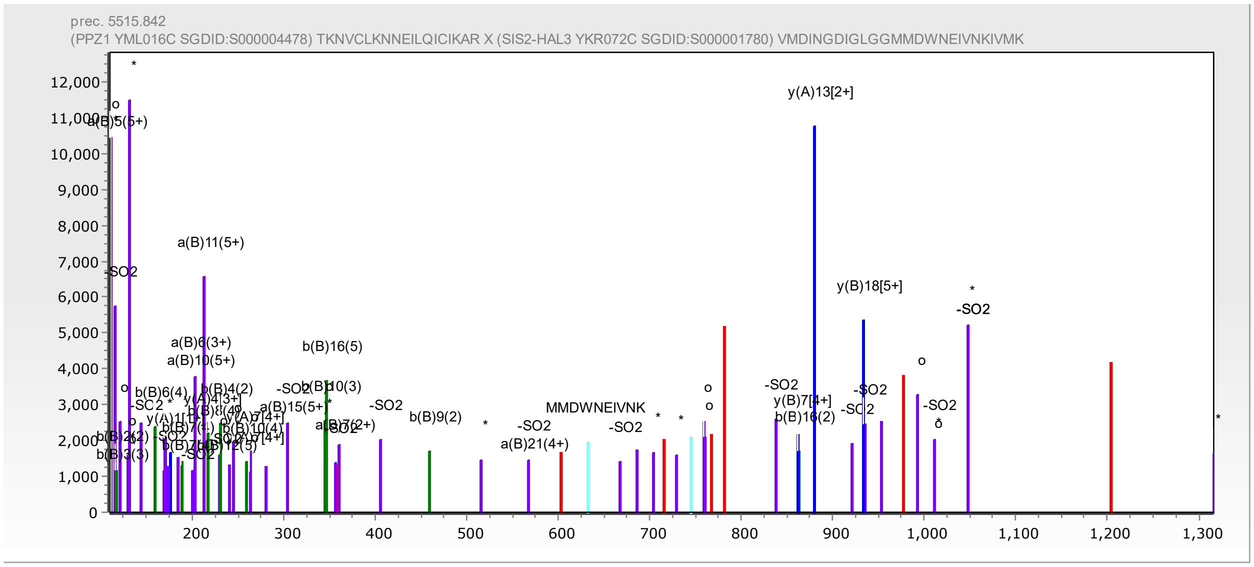
prec. 5515.842
(PPZ1 YML016C SGDID:S000004478) TKNVCLKNNEILQICIKAR X (SIS2-HAL3 YKR072C SGDID:S000001780) VMDINGDIGLGGMMDWNEIVNKIVMK
y(A)13[2+]
a(B)11(5+)
y(B)18[5+]
a(B)6(3+)
a(B)10(5+)
b(B)16(5)
b(B)10(3)
b(B)9(2)
a(B)21(4+)
MMDWNEIVNK
y(B)7[4+]
b(B)16(2)
-SO2
12,000
11,000
10,000
9,000
8,000
7,000
6,000
5,000
4,000
3,000
2,000
1,000
0
200
300
400
500
600
700
800
900
1,000
1,100
1,200
1,300

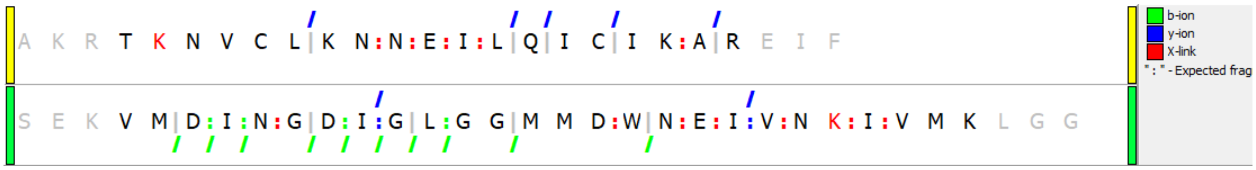
A K R T K N V C L K N:N:E:I:L Q I C I K:A R E I F
S E K V M D:I:N:G D:I:G L:G G M M D:W N:E:I:V:N K:I:V M K L G G
b-ion
y-ion
X-link
" : " - Expected frag

415 x 201

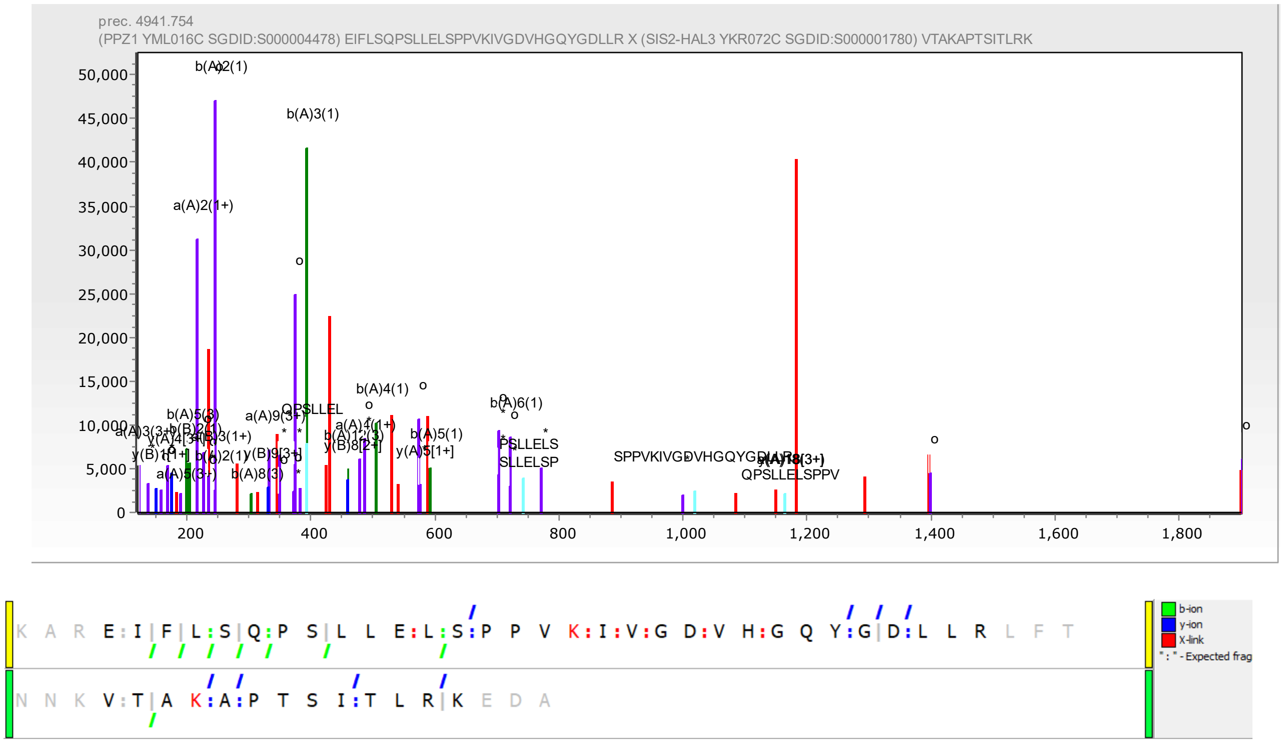

433 x 210

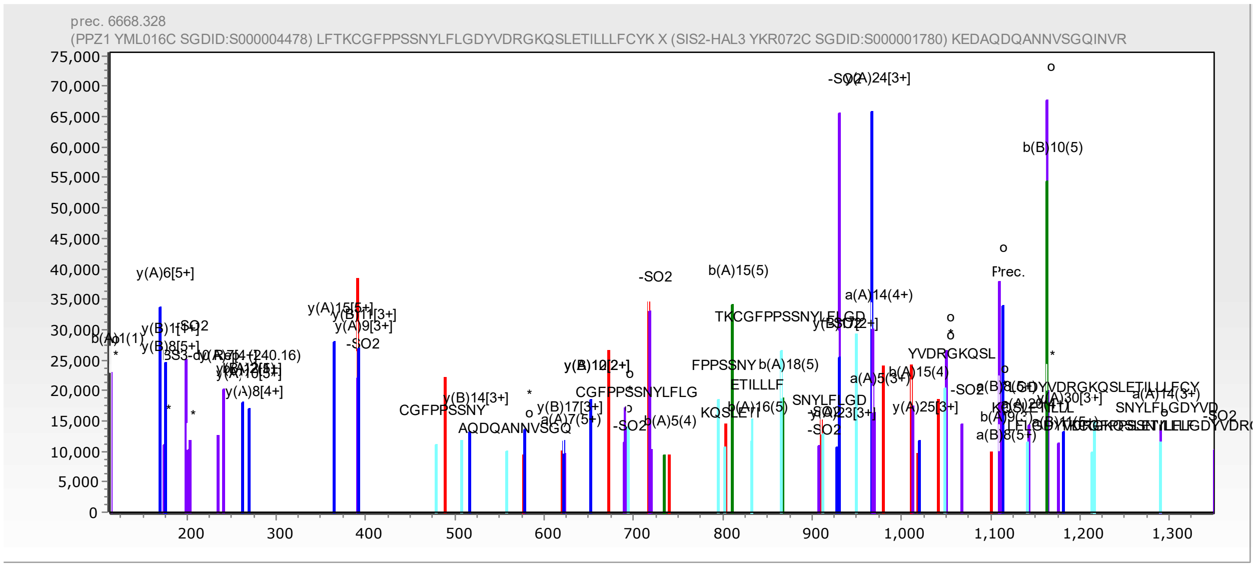
prec. 6668.328
(PPZ1 YML016C SGDID:S000004478) LFTKCGFPPSSNYLFLGDYVDRGKQSLETILLLFCYK X (SIS2-HAL3 YKR072C SGDID:S000001780) KEDAQDQANNVSGQINVR
y(A)6[5+]
-SO2
b(A)15(5)
b(B)10(5)
Prec.

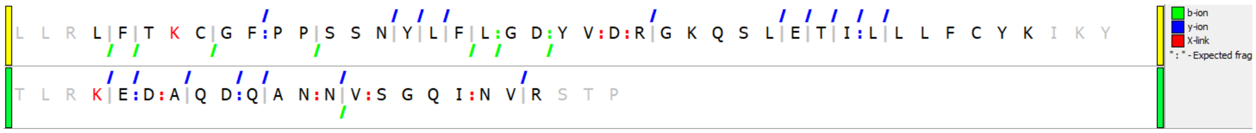
L L R L F T K C G F P P S S N Y L F L G D Y V D R G K Q S L E T I L L L F C Y K I K Y
T L R K E D A Q D Q A N N V S G Q I N V R S T P
b-ion
y-ion
X-link

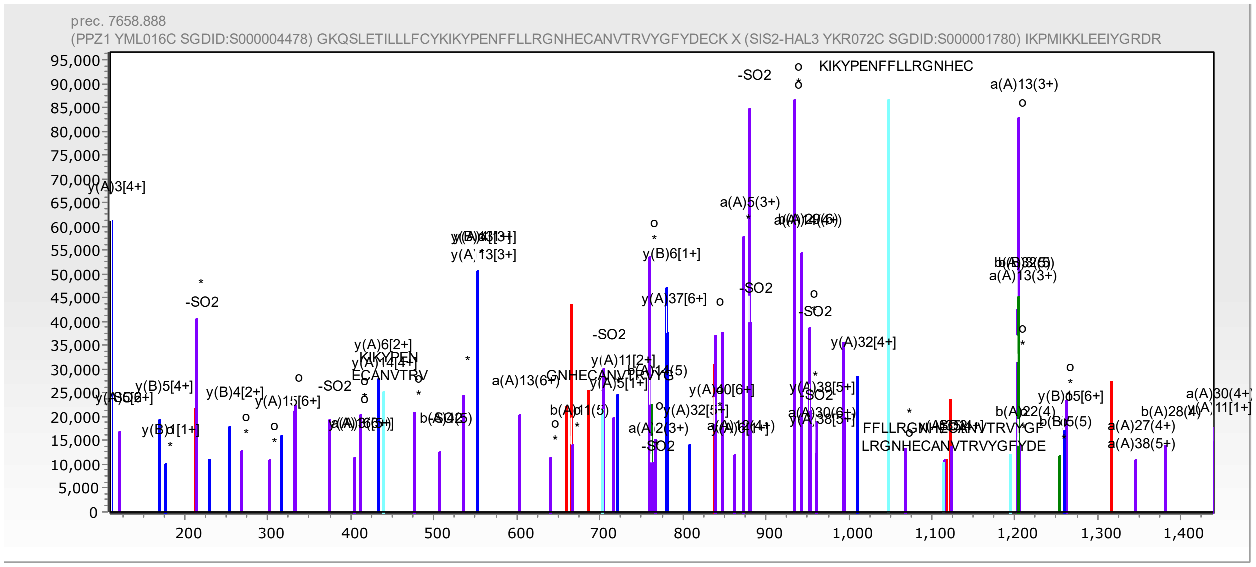
prec. 7658.888
(PPZ1 YML016C SGDID:S000004478) GKQSLETILLLFCYKIKYPENFFLLRGNHECANVTRVYGFYDECK X (SIS2-HAL3 YKR072C SGDID:S000001780) IKPMIKKLEEIYGRDR
95,000
90,000
85,000
80,000
75,000
70,000
65,000
60,000
55,000
50,000
45,000
40,000
35,000
30,000
25,000
20,000
15,000
10,000
5,000
0
200
300
400
500
600
700
800
900
1,000
1,100
1,200
1,300
1,400

453 x 280

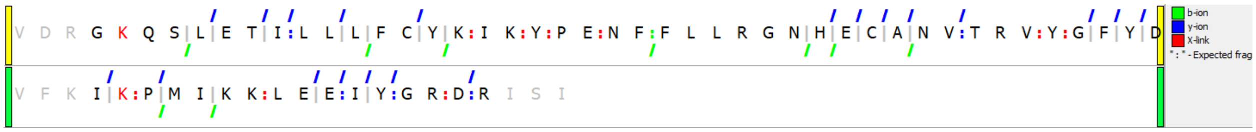
b-ion
y-ion
X-link
":" - Expected frag

468 x 201

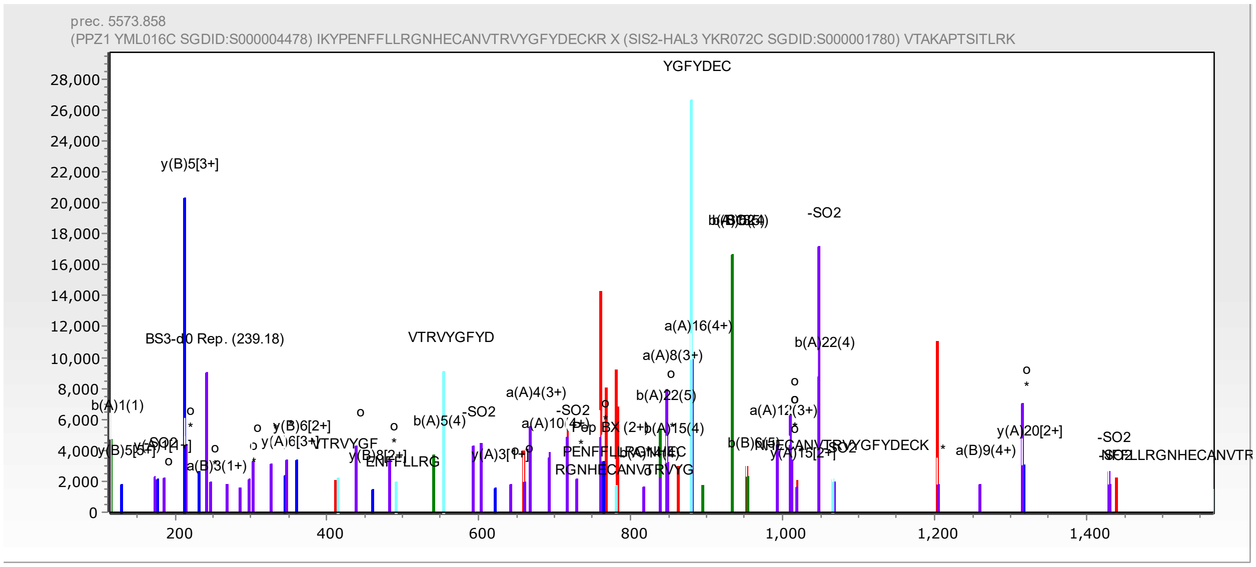
prec. 5573.858
(PPZ1 YML016C SGDID:S000004478) IKYPENFFLLRGNHECANVTRVYGFYDECKR X (SIS2-HAL3 YKR072C SGDID:S000001780) VTAKAPTSITLRK
YGFYDEC
y(B)5[3+]
-SO2
BS3-d0 Rep. (239.18)
VTRVYGFYD
a(A)16(4+)
a(A)8(3+)
b(A)22(4)
b(A)22(5)
a(A)4(3+)
a(A)12(3+)
y(A)20[2+]
b(A)5(4)
a(B)9(4+)
b(A)15(4)
a(B)3(1+)
y(B)6[2+]
VTRVYGF
ENFFLLRG
PENFFLLRGNHEC
RGNHECANVTRVYG
NHECANVTRVYGFYDECK
y(A)15[2+]
y(A)3[1+]
b(A)1(1)

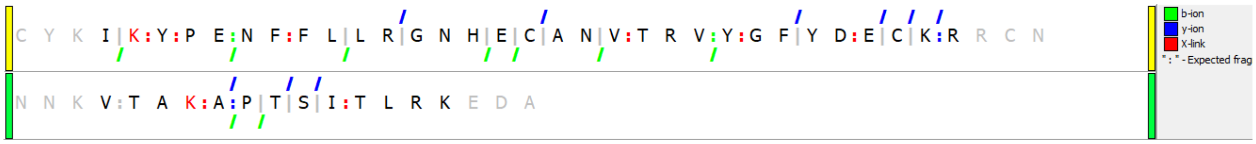
C Y K I K Y P E N F F L L R G N H E C A N V T R V Y G F Y D E C K R R C N
N N K V T A K A P T S I T L R K E D A
b-ion
y-ion
X-link
" : " - Expected frag

664 x 483

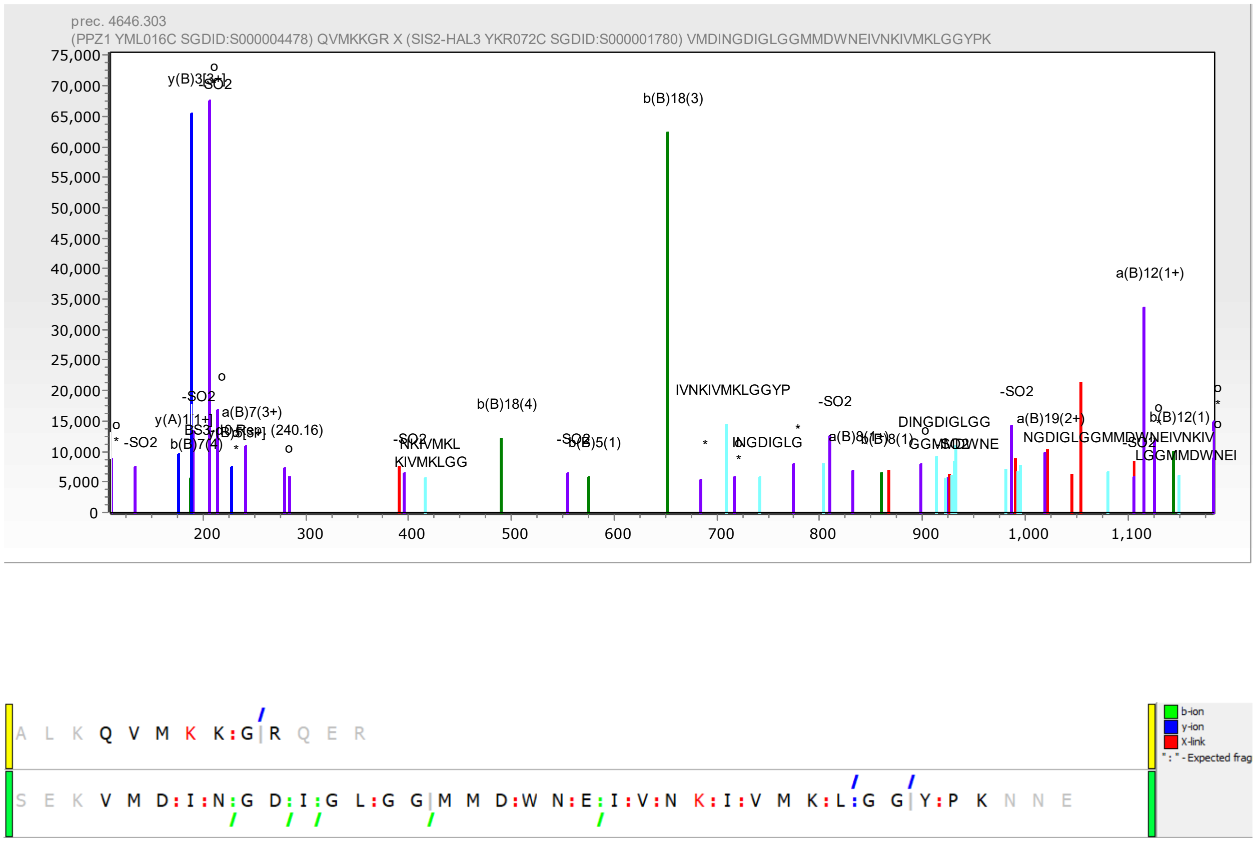

prec. 4646.303
(PPZ1 YML016C SGDID:S000004478) QVMKKGR X (SIS2-HAL3 YKR072C SGDID:S000001780) VMDINGDIGLGGMMDWNEIVNKIVMKLGGYPK
y(B)3[3+]
b(B)18(3)
b(B)18(4)
a(B)12(1+)
a(B)7(3+)
IVNKIVMKLGGYP
NGDIGLG
DINGDIGLGG
a(B)19(2+)
NGDIGLGGMMDWNEIVNKIV
LGGMMDWNEI
KIVMKLGG
NKIVMKL
b(B)5(1)
b(B)12(1)
-SO2
A L K Q V M K K:G R Q E R
S E K V M D:I:N:G D:I:G L:G G M M D:W N:E:I:V:N K:I:V M K:L:G G Y:P K N N E
b-ion
y-ion
X-link
":" - Expected frag

359 x 39

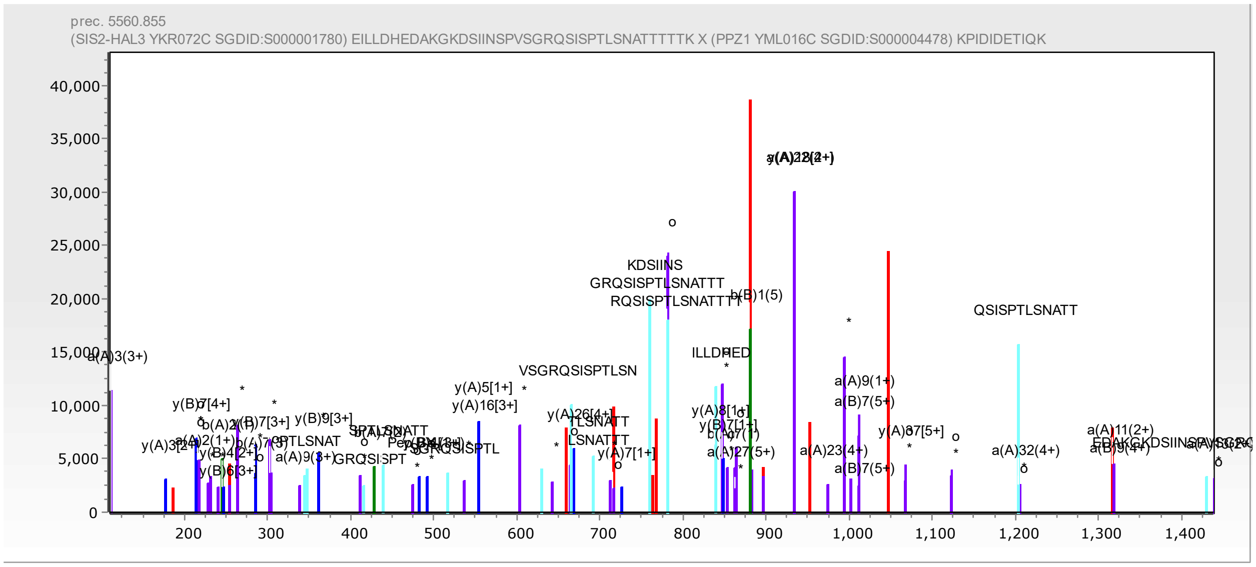

prec. 5560.855
(SIS2-HAL3 YKR072C SGDID:S000001780) EILLDHEDAKGKDSIINSPVSGRQSISPTLSNATTTTTK X (PPZ1 YML016C SGDID:S000004478) KPIDIDETIQK
40,000
35,000
30,000
25,000
20,000
15,000
10,000
5,000
0
200
300
400
500
600
700
800
900
1,000
1,100
1,200
1,300
1,400
KDSIINS
GRQSISPTLSNATTT
RQSISPTLSNATTTT
VSGRQSISPTLSN
QSISPTLSNATT
ILLDHED
y(A)5[1+]
y(A)16[3+]
a(A)9(1+)
a(B)7(5+)
a(A)23(4+)
a(A)32(4+)
a(A)11(2+)
y(A)7[1+]
TLSNATT
LSNATTT

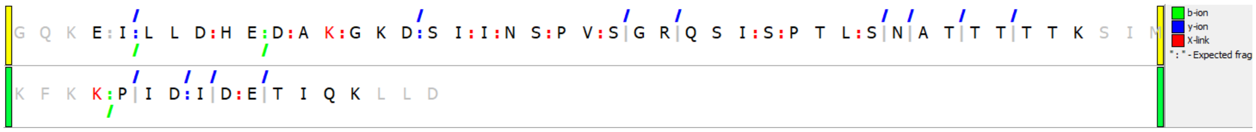

G Q K E I L L D H E D A K G K D S I I N S P V S G R Q S I S P T L S N A T T T T T K S I N
K F K K P I D I D E T I Q K L L D
b-ion
y-ion
X-link
":" - Expected frag

496 x 315

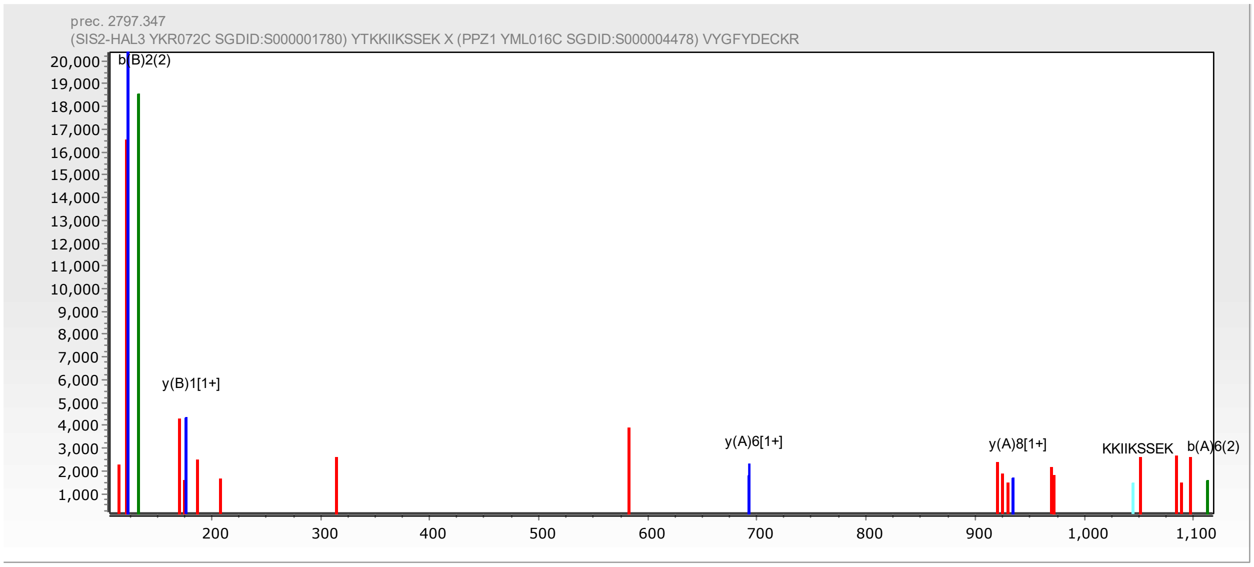

prec. 2797.347
(SIS2-HAL3 YKR072C SGDID:S000001780) YTKKIIKSSEK X (PPZ1 YML016C SGDID:S000004478) VYGFYDECKR
b(B)2(2)
y(B)1[1+]
y(A)6[1+]
y(A)8[1+]
KKIIKSSEK
b(A)6(2)

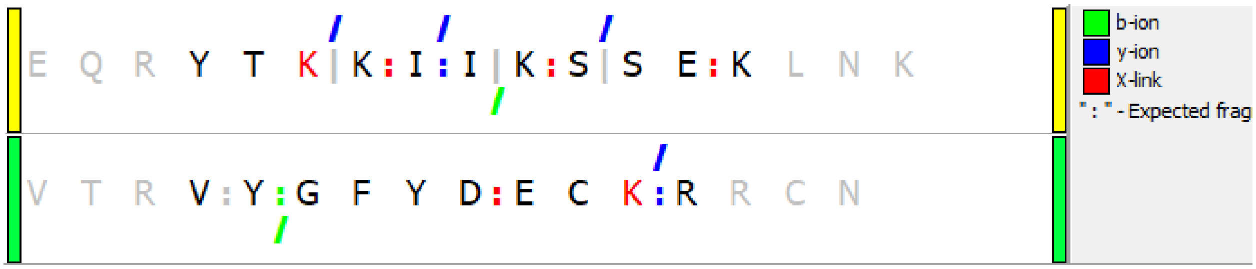

E Q R Y T K K I I K S S E K L N K
V T R V Y G F Y D E C K R R C N
b-ion
y-ion
X-link
":" - Expected frag

358 x 29

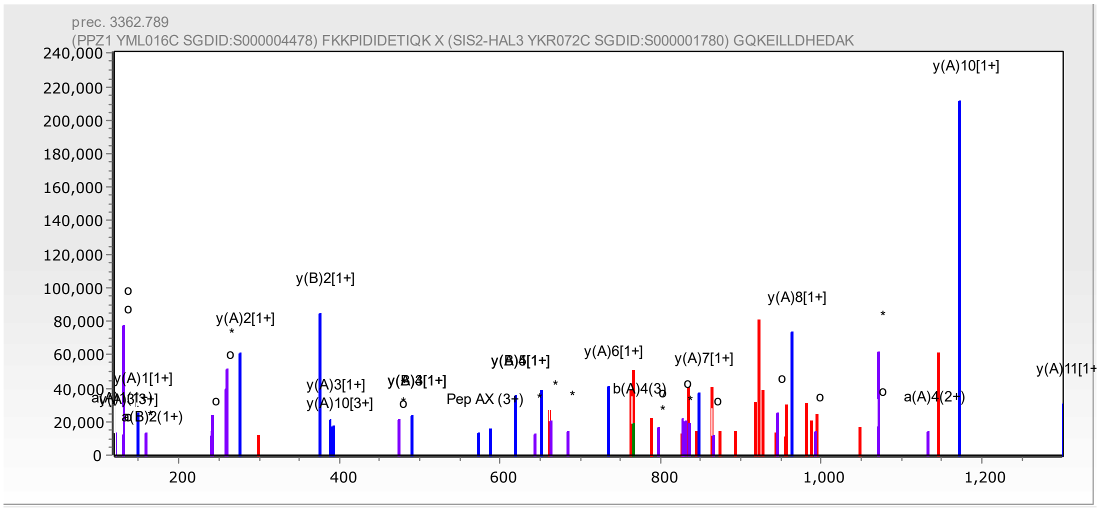
prec. 3362.789
(PPZ1 YML016C SGDID:S000004478) FKKPIDIDETIQK X (SIS2-HAL3 YKR072C SGDID:S000001780) GQKEILLDHEDAK
y(A)10[1+]
y(B)2[1+]
y(A)8[1+]
y(A)2[1+]
y(A)6[1+]
y(A)7[1+]
y(A)11[1+
y(A)1[1+]
y(A)3[1+]
y(A)10[3+]
Pep AX (3+)
a(A)4(2+)
a(B)2(1+)

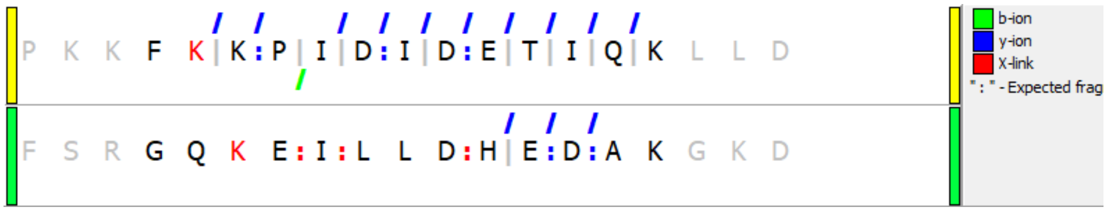
P K K F K | K : P | I | D : I | D : E | T | I | Q | K L L D
F S R G Q K E : I : L L D : H | E : D : A K G K D
b-ion
y-ion
X-link
" : " - Expected frag

358 x 315

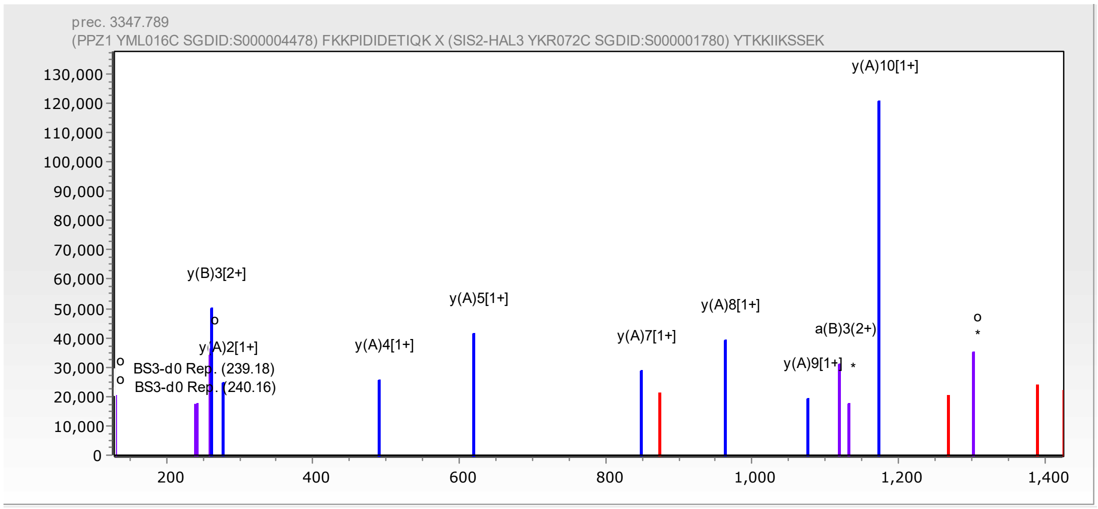

prec. 3347.789
(PPZ1 YML016C SGDID:S000004478) FKKPIDIDETIQK X (SIS2-HAL3 YKR072C SGDID:S000001780) YTKKIIKSSEK
y(A)10[1+]
y(B)3[2+]
y(A)5[1+]
y(A)8[1+]
y(A)7[1+]
a(B)3(2+)
y(A)4[1+]
y(A)9[1+]
BS3-d0 Rep. (239.18)
BS3-d0 Rep. (240.16)

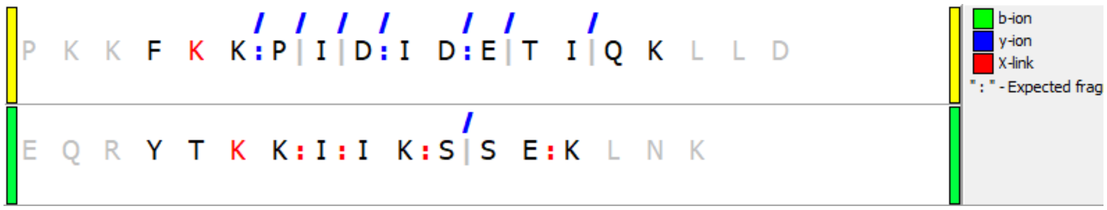

P K K F K K:P I D:I D:E T I Q K L L D
E Q R Y T K K:I:I K:S S E:K L N K
b-ion
y-ion
X-link
" : " - Expected frag

433 x 210

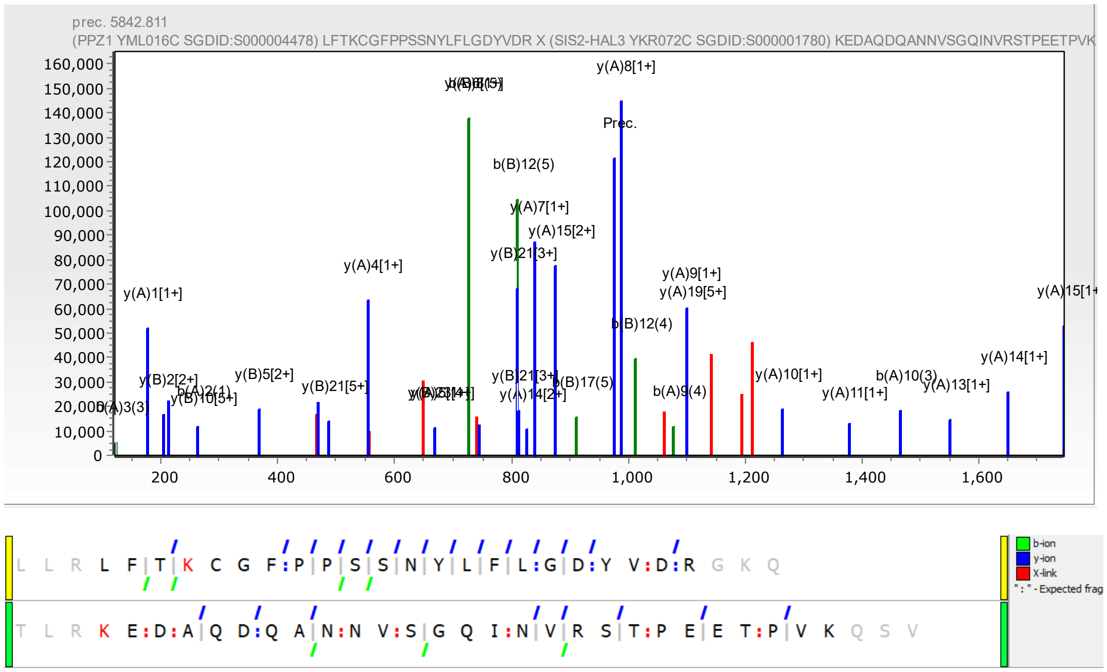
prec. 5842.811
(PPZ1 YML016C SGDID:S000004478) LFTKCGFPPSSNYLFLGDYVDR X (SIS2-HAL3 YKR072C SGDID:S000001780) KEDAQDQANNVSGQINVRSTPEETPVK
y(A)8[1+]
Prec.
b(B)12(5)
y(A)7[1+]
y(A)15[2+]
y(B)21[3+]
y(A)4[1+]
y(A)9[1+]
y(A)19[5+]
y(A)1[1+]
b(B)12(4)
y(A)14[1+]
y(B)5[2+]
y(B)21[5+]
y(A)10[1+]
b(A)10(3)
y(A)13[1+]
y(A)11[1+]
b(B)17(5)
y(A)14[2+]
b(A)9(4)
y(B)2[2+]
b(A)2(1)
y(B)10[5+]
b-ion
y-ion
X-link
" : " - Expected frag

584 x 90

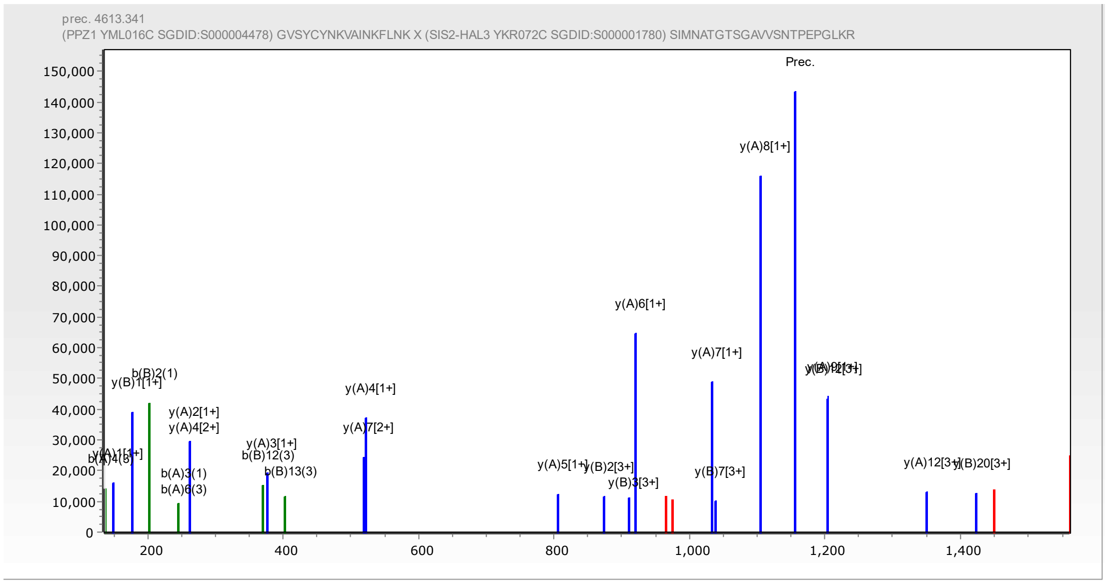

prec. 4613.341
(PPZ1 YML016C SGDID:S000004478) GVSYCYNKVAINKFLNK X (SIS2-HAL3 YKR072C SGDID:S000001780) SIMNATGTSGAVVSNTPEPGLKR
Prec.
y(A)8[1+]
y(A)6[1+]
y(A)7[1+]
y(A)4[1+]
y(A)2[1+]
y(A)4[2+]
y(A)3[1+]
b(B)12(3)
b(B)13(3)
y(A)5[1+]
y(B)2[3+]
y(B)3[3+]
y(B)7[3+]
y(A)12[3+]
y(B)20[3+]

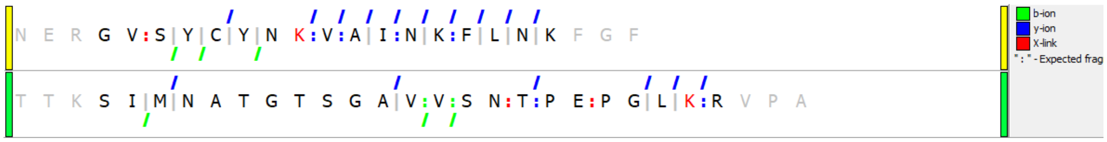

N E R G V S Y C Y N K V A I N K F L N K F G F
T T K S I M N A T G T S G A V V S N T P E P G L K R V P A
b-ion
y-ion
X-link
" : " - Expected frag

584 x 113

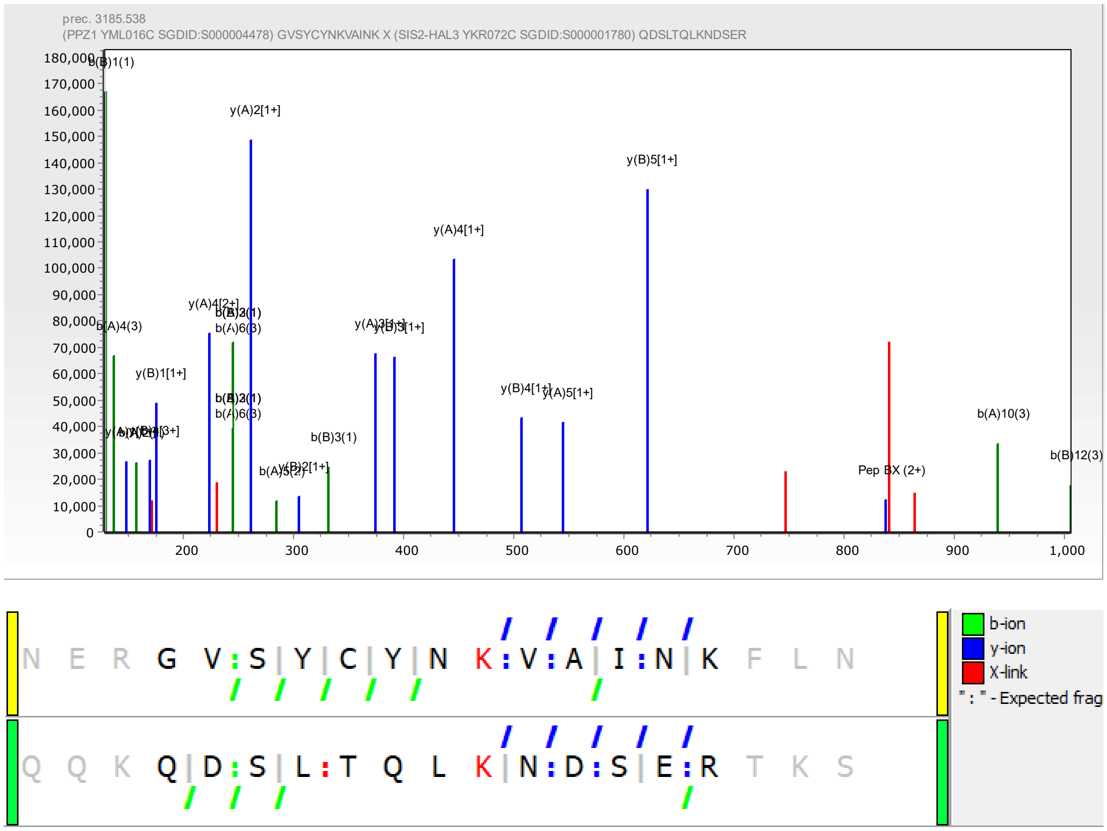

prec. 3185.538
(PPZ1 YML016C SGDID:S000004478) GVSYCYNKVAINK X (SIS2-HAL3 YKR072C SGDID:S000001780) QDSLTQLKNDSER
y(A)2[1+]
y(B)5[1+]
y(A)4[1+]
y(B)1[1+]
b(A)10(3)
b(B)12(3)
b(B)3(1)
Pep BX (2+)
b-ion
y-ion
X-link
" : " - Expected frag

584 x 323

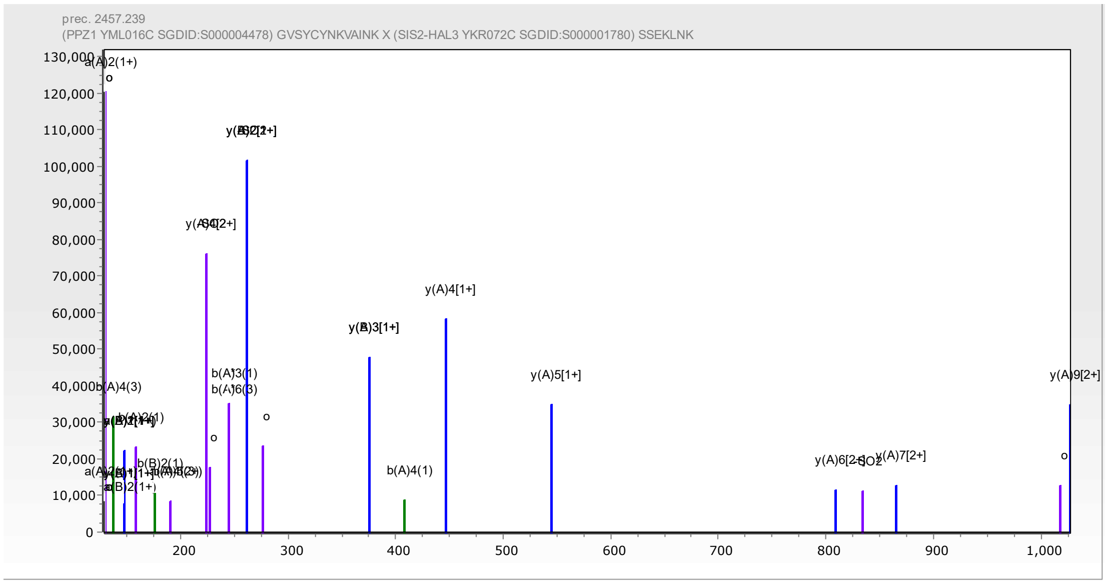
prec. 2457.239
(PPZ1 YML016C SGDID:S000004478) GVSYCYNKVAINK X (SIS2-HAL3 YKR072C SGDID:S000001780) SSEKLNK
a(A)2(1+)
y(A)4[1+]
y(A)5[1+]
y(A)9[2+]
y(A)7[2+]
b(A)4(1)
b(A)4(3)
b(A)3(1)
b(B)2(1)
a(B)2(1+)
130,000
120,000
110,000
100,000
90,000
80,000
70,000
60,000
50,000
40,000
30,000
20,000
10,000
0
200
300
400
500
600
700
800
900
1,000

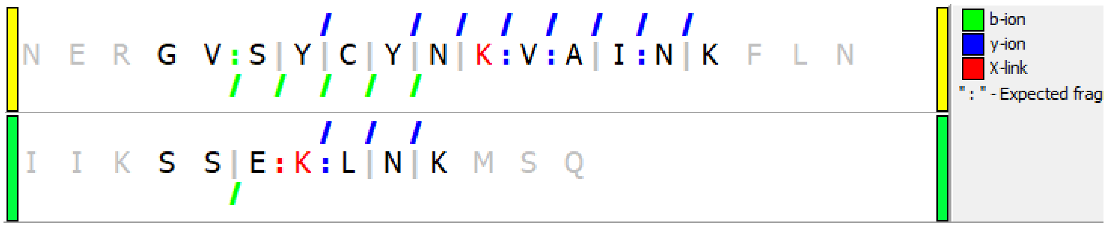
N E R G V : S | Y | C | Y | N | K : V : A | I : N | K F L N
I I K S S | E : K : L | N | K M S Q
b-ion
y-ion
X-link
" : " - Expected frag

389 x 90

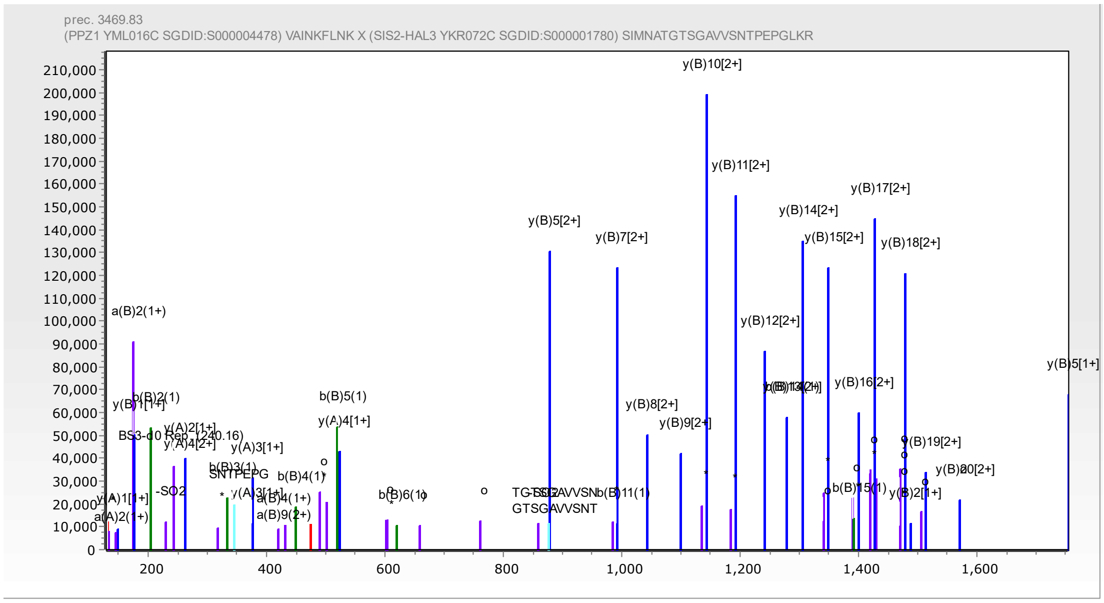

prec. 3469.83
(PPZ1 YML016C SGDID:S000004478) VAINKFLNK X (SIS2-HAL3 YKR072C SGDID:S000001780) SIMNATGTSGAVVSNTPEPGLKR
y(B)10[2+]
y(B)11[2+]
y(B)17[2+]
y(B)14[2+]
y(B)5[2+]
y(B)7[2+]
y(B)15[2+]
y(B)18[2+]
a(B)2(1+)
y(B)12[2+]
y(B)5[1+]
y(B)16[2+]
y(B)8[2+]
b(B)5(1)
y(A)4[1+]
y(B)9[2+]
y(B)19[2+]
-SO2
GTSGAVVSNT

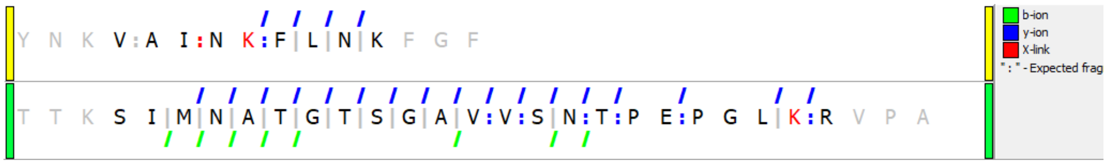

Y N K V : A I : N K : F L N K F G F
T T K S I M N A T G T S G A V : V : S N : T : P E : P G L K : R V P A
b-ion
y-ion
X-link
":" - Expected frag
